# A Catalytic Cross‐Olefination of Diazo Compounds with Sulfoxonium Ylides

**DOI:** 10.1002/anie.201809934

**Published:** 2018-11-08

**Authors:** James D. Neuhaus, Adriano Bauer, Alexandre Pinto, Nuno Maulide

**Affiliations:** ^1^ Institute of Organic Chemistry University of Vienna Währinger Straße 38 1090 Vienna Austria

**Keywords:** cross-olefination, diazo compounds, homogenous catalysis, ruthenium, sulfur ylides

## Abstract

A ruthenium‐catalysed cross‐olefination of diazo compounds and sulfoxonium ylides is presented. Our reaction design exploits the intrinsic difference in reactivity of diazo compounds and sulfoxonium ylides as both carbene precursors and nucleophiles, which results in a highly selective reaction.

The fascinating properties of alkenes have captured the imagination of chemists ever since in 1795 a team of Dutch researchers observed that the reaction between ethylene and chlorine forms a colourless liquid.^1^ This observation led the authors to name ethene “gaz huileux” (i.e., oil‐forming gas). Soon the term was changed to “gaz oléfiant”, and today, over 200 years later, it is still common practice to use the word “olefin” when referring to an alkene.

In the last 70 years, tremendous developments in the ability to form C=C double bonds have been recognized with Nobel prizes and have become textbook knowledge. Examples include the Wittig and related reactions^2^, ^3^ and olefin metathesis.^4^ A conceptually appealing but seldom realized retrosynthetic disconnection of alkenes relies on the union of two carbenes. Indeed, early work showed that the metal‐catalysed homocoupling of diazo compounds is a valuable alternative for the generation of symmetric alkenes.^5^ Later investigations into the intermolecular cross‐coupling of diazo compounds demonstrated that more effective couplings can be achieved when the nature of the two coupling partners is sufficiently different.^6^, ^7^ An early example by Zotto and co‐workers showed that acceptor‐substituted diazo compounds can be selectively cross‐coupled with TMS diazomethane with high stereoselectivity (Scheme 1).6a This concept was recently extended by Liu and co‐workers, whereby alkyl‐substituted diazo compounds were generated in situ and selectively cross‐coupled to acceptor‐substituted diazo compounds by silver catalysis.7e However, poor stereoselectivity, with 1:1 mixtures of *E*/*Z* olefin products, was observed. Earlier, Davies and co‐workers had shown that donor–acceptor diazo compounds can be cross‐coupled selectively to acceptor‐substituted diazo compounds by rhodium catalysis. Stereoselectivity was generally high in favour of the *E* olefin.7a Although the scope was extended by Sun and co‐workers, this method requires both an electron‐withdrawing and an electron‐donating group adjacent to the diazomethane moiety of at least one of the reaction partners.7a, 7c–7e An additional procedure by Wang and co‐workers relies on the use of cyclopropenes as carbene precursors, which are then coupled to diazo compounds.6d The same group published an interesting coupling of diazo compounds with in situ generated difluorocarbene.6e

**Scheme 1:**
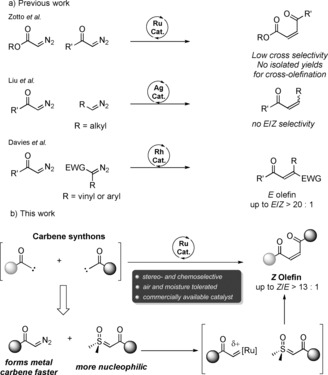
Previously reported cross‐olefinations and this work.

Sulfonium and sulfoxonium ylides have witnessed a renaissance in contemporary catalysis beyond the well‐known Johnson–Corey–Chaykovsky reactions,^8^ namely in C−H functionalisation,^9^ N−H insertion,^10^ and cycloisomerisation reactions.^11^ Their popularity is owed to the fact that they are easy to prepare, readily purified, and considerably safer to handle than their diazo counterparts.

Although sulfoxonium ylide dimerization has been observed indirectly, it has never been used for the effective synthesis of olefins. This is probably also due to the fact that the generated products (electron‐poor olefins) are also good substrates for Johnson–Corey–Chaykovsky cyclopropanation under the reaction conditions.^12^ Indeed, sulfoxonium ylides are generally better nucleophiles than their diazo analogues, but tend to undergo decomposition to the metal carbene at much lower rates than their diazo counterparts, rendering a hypothetical catalytic sulfoxonium ylide (cross)‐coupling a difficult prospect.^13^

This led us to speculate that the catalytic cross‐coupling of a diazo compound with a sulfoxonium ylide should be possible. We surmised that formation of a metal carbene should take place faster from the diazo compound precursor, and that the resulting electrophilic carbene would be attacked preferentially by the more nucleophilic sulfoxonium ylide. However, we were uncertain whether the coupling product (a Michael acceptor) would be prone to conjugate addition by the sulfoxonium ylide.

In our first trials, a range of iridium(I) and rhodium(II) catalysts were investigated for the cross‐olefination of diazoester **1 a** and sulfoxonium ylide **2 a**, owing to their well‐documented proficiency in metallocarbene formation.7a, 14 Those preliminary experiments (Table 1, entries 1 and 2; see the Supporting Information for further experiments) led to low but promising yields of the desired product, together with diethyl maleate/diethyl fumarate resulting from homodimerization of diazoester **1 a** as the main side product. Importantly, homodimerization of the sulfoxonium ylide **2 a** was virtually absent, corroborating our initial hypothesis. Unreacted sulfoxonium ylide could be removed completely along with the catalyst during workup, resulting in a clean and easy‐to‐analyse crude ^1^H NMR spectrum. Cyclopropanation side products were never observed.^15^ In the course of catalyst screening, we found that the cheap ruthenium complex [Ru(*p*‐cymene)Cl_2_]_2_ 16 displays the highest efficiency for this cross‐olefination. Further optimization of the conditions led to good isolated yields above 70 % with a *Z*/*E* ratio of 9:1 (Table 1, entry 5).

**Table 1:** Optimization of the cross‐olefination.^[a]^ 
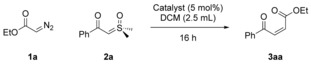

Entry	Catalyst	*T*	**3 aa** [%]^[c]^	*Z*/*E*
1	[Ir(COD)Cl]_2_	RT	13	1.0:1
2	Rh_2_(OAc)_4_	RT	20	1.9:1
3	[Ru(*p*‐cymene)Cl_2_]_2_	RT	32	1.9:1
4	[Ru(*p*‐cymene)Cl_2_]_2_	−78 °C to RT	53	7.8:1
*5* ^*[b]*^	*[Ru(p‐cymene)Cl_2_]_2_*	*−78 °C to RT*	*71* ^*[d]*^	*9.0:1*
6^[b]^	Rh_2_(Esp)_2_	−78 °C to RT	10	1:9.0
7^[b]^	Rh_2_(OPiv)_2_	−78 °C to RT	17	1:7.5

[a] All reactions were performed on 0.2 mmol scale (diazo compound) under air. [b] With 2.0 equiv of the sulfoxonium ylide. [c] Yields determined by ^1^H NMR spectroscopy using mesitylene as an internal standard. [d] Yield of isolated product.

With optimized conditions in hand, we examined the ylide scope (Scheme 2).^17^ Pleasingly, electron‐poor (**2 b**–**2 d**, **2 h**) sulfoxonium ylides afforded similar yields as well as *Z*/*E* selectivities. The *para*‐substituted substrates **2 b**/**2 h** gave particularly selective olefination, with *Z*/*E* ratios up to 13:1. Electron‐rich substrates (**2 e**/**2 i**) showed lower selectivity. Noteworthy, the aryl iodide **2 f** reacted smoothly without competing oxidative addition. The product **3 ai**, which has shown antimicrobial activity (*M. tuberculosis*), was prepared in a single step.^18^

**Scheme 2:**
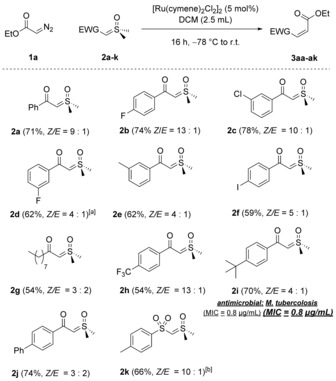
Sulfoxonium ylide substrate scope. The *Z*/*E* ratios were determined by ^1^H NMR analysis of the crude product mixture. All yields are for pure, isolated material unless indicated otherwise. [a] ^1^H NMR yield determined using mesitylene as an internal standard. [b] DMF was used as a cosolvent.

The ketone moiety on the ylide was not a prerequisite for successful cross‐olefination as sulfone **3 ak** afforded comparable yields and high selectivities.

At this juncture, the substrate scope for the diazo compound was investigated (Scheme 3). As depicted, the reaction is general for a range of diazoesters. Notably, several alkenes (**1 c**/**1 i**), a silane (**1 e**), and even an alkyne (**1 b**) were well tolerated, with *Z*/*E* ratios of up to 11:1. No traces of competing cyclopropanation of the unsaturated moieties were observed. Furthermore, esters of functionalized terpene alcohols such as cholesterol (**1 f**), citronellol (**1 l**), or a β‐pinene derivative (**1 j**) were smoothly converted into the desired olefins.^19^

**Scheme 3:**
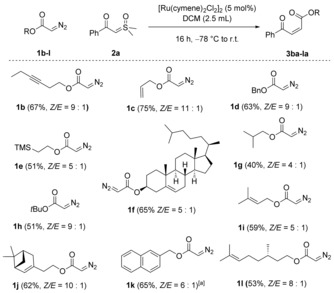
Diazo substrate scope. The *Z*/*E* ratios were determined by ^1^H NMR analysis of the crude product mixture. All yields refer to pure, isolated material unless indicated otherwise. [a] ^1^H NMR yield determined using mesitylene as an internal standard.

Under these conditions, donor–acceptor diazoesters (popularised by the elegant work of Davies^20^), such as methyl (*E*)‐2‐diazo‐4‐phenylbut‐3‐enoate (**4**), were typically recovered, suggesting that conversion into the metal carbene did not take place. Upon changing to rhodium(II) catalysis instead of ruthenium(II) (Scheme 4), the corresponding α‐ketoester **5** was observed (21 % and 49 % starting material, NMR yield). This suggests that our procedure is orthogonal to the method of Davies.7a

**Scheme 4:**
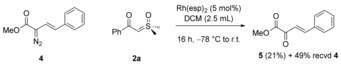
Unexpected reaction of a donor–acceptor diazo compound. ^1^H NMR yields determined using mesitylene as an internal standard.

A direct comparison of the cross‐olefination procedure reported herein with the cross‐olefination of two diazo compounds reveals that yield and selectivity are considerably higher when sulfoxonium ylides are employed (Scheme 5). Moreover, while the cross‐olefination of sulfoxonium ylides and diazoesters delivers the product of homodimerization of the ester moiety (product **3 ma**; see Scheme 5) as the only undesired side product in small amounts, cross‐olefination of two diazo compounds results in a mixture of all three possible coupling products with virtually no selectivity. (In the event, the desired cross‐coupled product **3 aa** is not even the major product.)

**Scheme 5:**
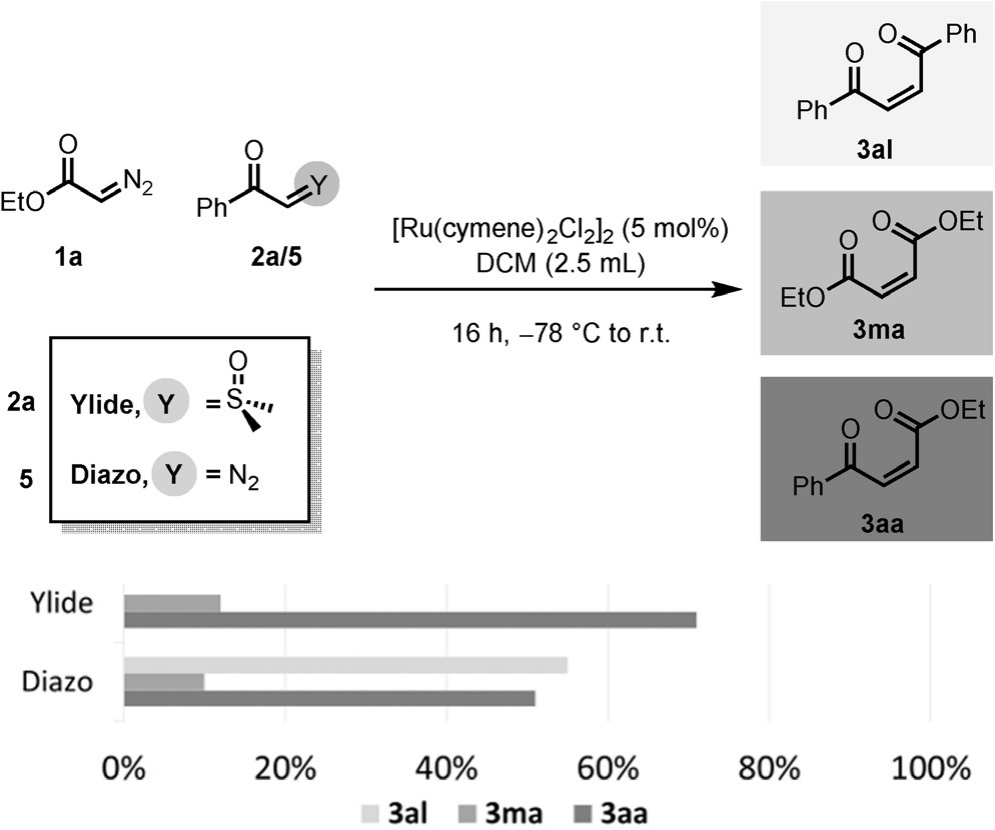
Direct comparison of the cross‐olefination procedure with the cross‐coupling of two different diazo compounds. Reaction conditions: EDA (1.0 equiv), acetophenone derivative (2.0 equiv); *Z*/*E* ratios determined by ^1^H NMR analysis of the crude product mixture; ^1^H NMR yields determined using mesitylene as an internal standard.

During the optimization studies, several quenching agents were investigated. While pyridine, pyrimidine, and dimethyl sulfide shut down the reaction, triphenylphosphine had an additional effect: *Z*/*E* diastereomeric mixtures were converted completely into the *E* isomer when substoichiometric amounts of PPh_3_ were added to the reaction mixture. Further studies showed that this isomerization^21^ takes place not only under the reaction conditions but also in solutions of isolated products (Scheme 6).

**Scheme 6:**
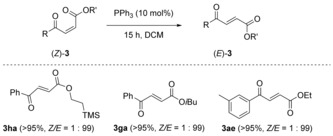
Isomerization to *E* olefins.

In conclusion, a novel ruthenium‐catalysed cross‐olefination of diazo compounds and sulfoxonium ylides has been presented. Our reaction design exploits the intrinsic difference in reactivity of diazo compounds and sulfoxonium ylides as both carbene precursors and nucleophiles, resulting in a highly selective reaction that nicely complements known, often less selective diazo–diazo coupling reactions. This results in the generation of olefin products with high *Z* selectivity.

## Conflict of interest

The authors declare no conflict of interest.

## Supporting information

As a service to our authors and readers, this journal provides supporting information supplied by the authors. Such materials are peer reviewed and may be re‐organized for online delivery, but are not copy‐edited or typeset. Technical support issues arising from supporting information (other than missing files) should be addressed to the authors.

Supplementary
