# Supplementary material for: A Catalytic Cross‐Olefination of Diazo Compounds with Sulfoxonium Ylides
Source: Angew Chem Int Ed Engl. 2018 Nov 8;57(49):16215–8. doi: 10.1002/anie.201809934 (PMC6283242; doi:10.1002/anie.201809934)
Supplement: Supplementary file 1 — Supplementary [file ANIE-57-16215-s001.pdf]

Angewandte Chemie
Eine Zeitschrift der Gesellschaft Deutscher Chemiker

Supporting Information

A Catalytic Cross-Olefination of Diazo Compounds with Sulfoxonium Ylides

*James D. Neuhaus+, Adriano Bauer+, Alexandre Pinto, and Nuno Maulide**

anie_201809934_sm_miscellaneous_information.pdf

Table of Contents

I. Preparation of starting material 2

a. Sulfoxonium ylide synthesis 2

b. Diazo compound synthesis 3

II. Cross olefination reaction 4

III. Isomerization with triphenylphosphine 6

IV. Characterization 7

V. Spectra 15

General information

Unless otherwise stated, all glassware was flame-dried before use and all reactions were performed under an atmosphere of argon. All solvents were distilled from appropriate drying agents prior to use. All reagents were used as received from commercial suppliers unless otherwise stated. Reaction progress was monitored by thin layer chromatography (TLC) performed on aluminium plates coated with silica gel F254 with 0.2 mm thickness. Chromatograms were visualized by fluorescence quenching with UV light at 254 nm or by staining using potassium permanganate. Flash column chromatography was performed using silica gel 60 (230-400 mesh, Merck and co.). Neat infrared spectra were recorded using a Perkin-Elmer Spectrum 100 FT-IR spectrometer. Wavenumbers (νmax) are reported in cm^{-1}. Mass spectra were obtained using a Finnigan MAT 8200 or (70 eV) or an Agilent 5973 (70 eV) spectrometer, using electrospray ionization (ESI). All ^{1}H NMR and ^{13}C NMR spectra were recorded using a Bruker AV-400 or AV-600 spectrometer at 300K. Chemical shifts were given in parts per million (ppm, δ), referenced to the solvent peak of CDCl3, defined at δ = 7.26 ppm (^{1}H-NMR) and δ = 77.16 (^{13}C NMR). Coupling constants are quoted in Hz (J). 1H NMR splitting patterns were designated as singlet (s), doublet (d), triplet (t), quartet (q). Splitting patterns that could not be interpreted or easily visualized were designated as multiplet (m) or broad (br).

I. Preparation of starting material

a. Sulfoxonium ylide synthesis

General procedure A

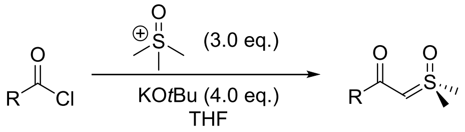

The reaction was carried out under air. Potassium *tert*-butoxide (4.0 eq.) was suspended in THF (1 mL/1 mmol KO*t*Bu) and trimethylsulfoxonium iodide (3.0 eq.) at once in a three-necked flasked fitted with a condenser. The mixture was refluxed for 2 hours resulting in a yellow cloudy suspension. The reaction was cooled down to 0 °C and acyl chloride was added dropwise as a solution in THF (1.0 eq. – 1 mol/L). After warming up to room temperature the mixture was stirred for 3 h. Afterwards volatiles were removed under reduced pressure and the residue was dissolved in equal amounts of EtOAc and Water. After extracting the aqueous phase with EtOAc (3 x the volume of the aqueous phase) the combined organic phase was dried over Na_2SO_4, filtered and the solvent was removed under reduced pressure. The crude was purified by column chromatography (typical eluent – acetone : heptanes 50 : 50 to 75 : 25 vol%).

General procedure B

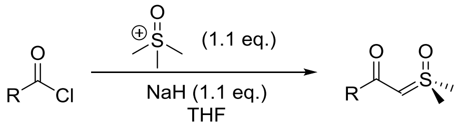

The reaction was carried out under air. Trimethylsulfoxonium iodide (1.1 eq.) was suspended in THF (4.0 mL/1 mmol Acylchloride) in a three-necked flasked fitted with a condenser and sodium hydride (1.1 eq.) was added portionwise. The mixture was refluxed for 2 hours resulting in a yellow cloudy suspension. The reaction was cooled down to 0 °C and acyl chloride was added dropwise as a solution in THF (1.0 eq. – 1 mol/L). After warming up to room temperature the mixture was stirred for 3 h. Afterwards volatiles were removed under reduced pressure and the residue was dissolved in equal amounts of EtOAc and Water. After extracting the aqueous phase with EtOAc (3 x the volume of the aqueous phase) the combined organic phase was dried over Na_2SO_4, filtered and the solvent was removed under reduced pressure. The crude was purified by column chromatography (typical eluent – acetone : heptanes 50 : 50 to 75 : 25 vol%).

General procedure C

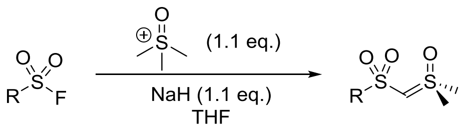

Trimethylsulfoxonium iodide (1.1 eq.) was suspended in THF (1.5 mL/1 mmol sulfonyl flouride) in a three-necked flasked fitted with a condenser and sodium hydride (1.1 eq.) was added portionwise. The mixture was stirred at room temperature for 30 minutes. The reaction was cooled down to 0 °C and sulfonyl fluoride was added dropwise as a solution in THF (1.0 eq. – 2 mol/L). After warming up to room temperature the mixture was stirred for 30 minutes. Then the mixture was stirred for further 3 h at 55 °C. Afterwards volatiles were removed under reduced pressure and the residue was dissolved in equal amounts of EtOAc and Water. After extracting the aqueous phase with EtOAc (3 x the volume of the aqueous phase) the combined organic phase was dried over Na_2SO_4, filtered and the solvent was removed under reduced pressure. The crude was purified by column chromatography (typical eluent – acetone : heptanes 50 : 50 to 75 : 25 vol%)

b. Diazo compound synthesis

General procedure D

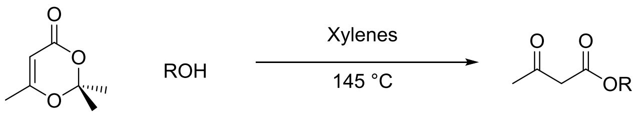

The reaction was carried out under air. 2,2,6-trimethyl-4H-1,3-dioxin-4-one (1.0 eq.) and the alcohol (1.0 eq.) was dissolved in xylenes (0.5 mL/mmol) in an Erlenmeyer flask. The mixture was stirred and heated to 145 °C. After 30 minutes the mixture was cooled down to room temperature and the solvent was removed under reduced pressure. The crude residue was purified by column chromatography (typical eluent - Et_2O : heptanes 10 : 90 to 40 : 60 vol%).

General procedure E

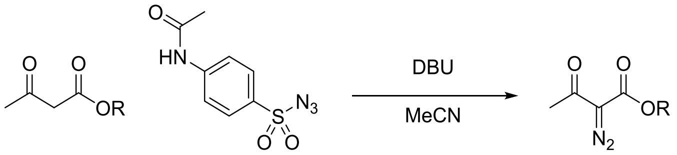

The reaction was carried out under air. The acetoacetate ester (1.0 eq.) and 4-acetamidobenzenesulfonyl azide (1.1 eq.) were dissolved in MeCN (3 mL/mmol) in a round bottom flask. The mixture was cooled to 0 °C and DBU (1.4 eq.) was added dropwise. The mixture was stirred until starting material disappeared (TLC). The solvent was evaporated after the mixture was filtered through celite®. Purification of the crude by column chromatography gave the desired product (typical eluent - EtOAc : heptanes 5 : 95 to 20 : 80 vol%).

General procedure F

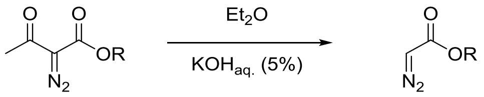

The reaction was carried out under air. The diazo acetoacetate ester was dissolved in Et_2O (5 mL /mmol) and H_2O (5 mL/mmol) was added. Under stirring LiOH (10.0 eq.) was added and the mixture was allowed to stir vigorously for 14 h at room temperature. The aqueous phase was extracted with Et_2O (3 x the volume of the aqueous phase), the combined organic layer was dried over Na_2SO_4 and the solvent was removed under reduced pressure. The crude product was purified by a short column chromatography (typical eluent - EtOAc : heptanes 10 : 90 to 30 : 70 vol%). or a silica plug. (Note: The α-diazoesters synthesized for this report are stable on silica)

General procedure G

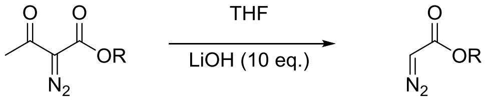

The reaction was carried out under air. The diazo acetoacetate ester was dissolved in THF (4 mL /mmol) and H_2O (6 mL/mmol) was added. Under stirring LiOH (10.0 eq.) was added and the mixture was allowed to stir vigorously for 14 h at room temperature. The aqueous phase was extracted with Et_2O (3 x the volume of the aqueous phase), the combined organic layer was dried over Na_2SO_4 and the solvent was removed under reduced pressure. The crude product was purified by a short column chromatography (typical eluent - EtOAc : heptanes 10 :90 to 30 : 70 vol%) or a silica plug. (Note: The α-diazoesters synthesized for this report are stable on silica)

II. Cross olefination reaction

General procedure H

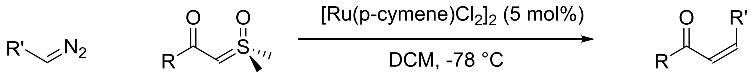

The reaction was carried out under air. In a microwave vial the sulfoxonium ylide (0.2 mmol) and the diazo compound (0.1 mmol) were dissolved in DCM (2 mL). In a separate microwave vial the ruthenium catalyst (5 μmol) was dissolved in DCM (0.5 mL). Both solutions were cooled to -78 °C in a dry ice bath for 5 - 10 minutes. Thereafter, the catalyst solution was added dropwise to the solution containing the ylide and the diazo compound. The vial was kept in the dry ice bath for 16 h and was allowed to warm up slowly to room temperature. Afterwards, the reaction mixture was filtered through a short silica plug, which was washed several times with DCM. The solvent was evaporated and the residue was purified by column chromatography (typical eluent - Et_2O : heptanes 5 : 95 to 20 : 80 vol%).

Diazo ketones and a diazo lactone were investigated with the following results:

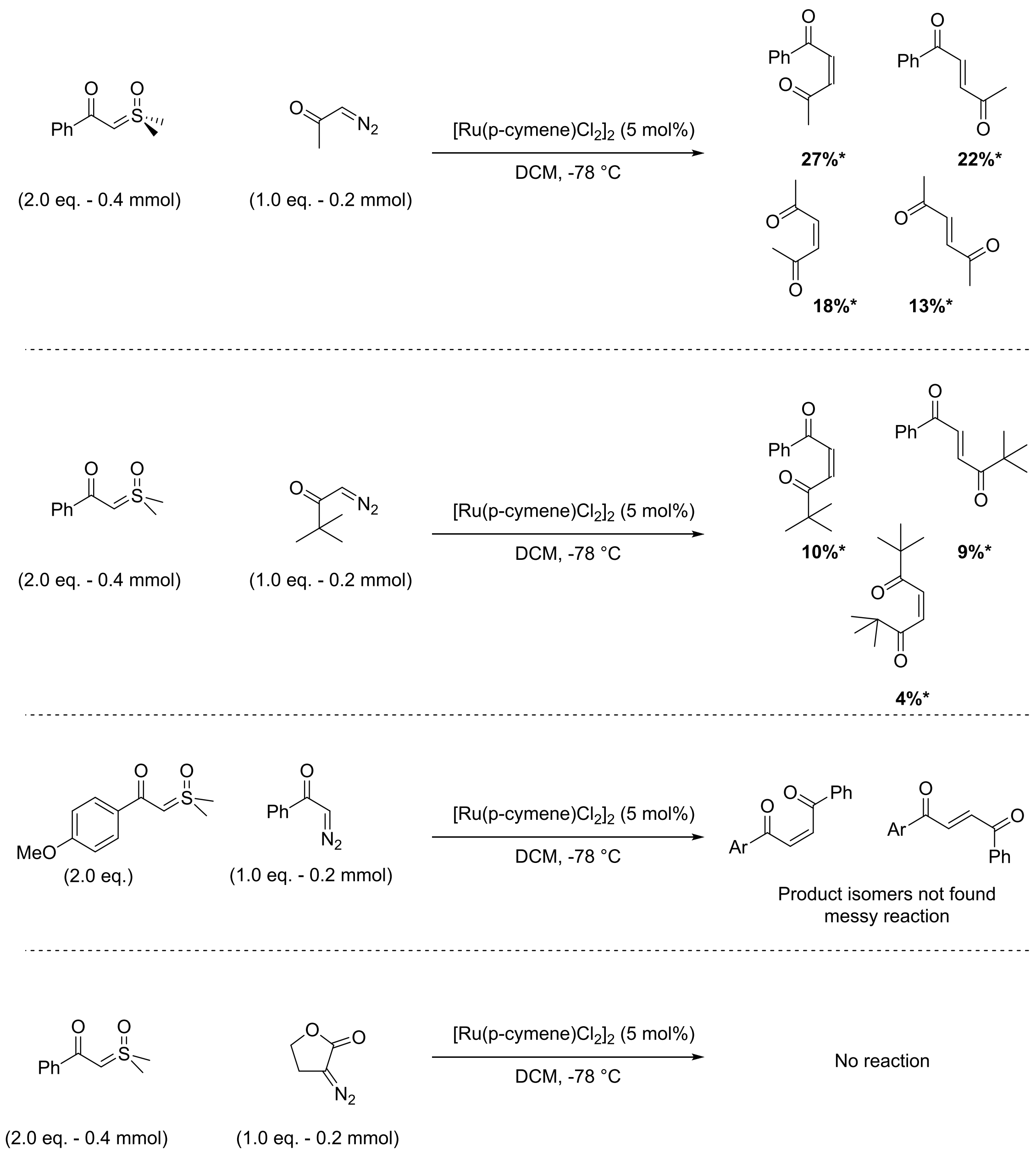

*^{1}H NMR yields with mesitylene as internal standard.

The reaction with sulfoxonium ylides has been investigated. The *in situ* generated dimethyl sulfide, quenches the catalysis efficiently. Indeed, these reactions show yields corresponded to approximately one catalyst turn-over (see below).

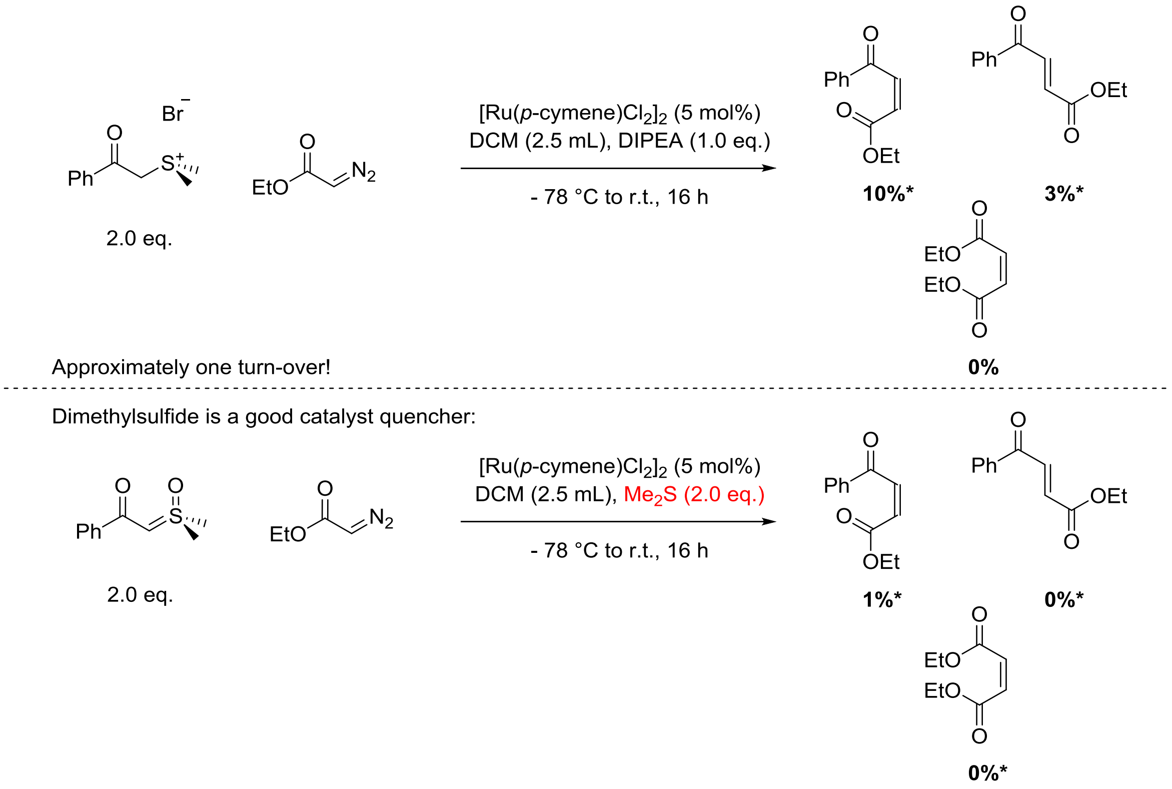

*^{1}H NMR yields with mesitylene as internal standard.

III. Isomerization with triphenylphosphine

General procedure I

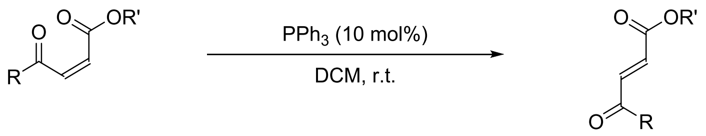

The reaction was carried out under air. The Z olefin was dissolved in DCM (10 mL/mmol) in a dram vial and stirred for 15 h at room temperature. The solvent was evaporated under reduced pressure and the residue was purified by column chromatography (typical eluent - Et_2O : heptanes 5 : 95 to 20 : 80 vol%).

IV. Characterization

Ethyl 2-diazoacetate (**1a**)
1a was purchased by Sigma-Aldrich.

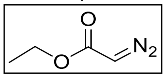

Hex-3-yn-1-yl 2-diazoacetate (**1b**)
1b was prepared by following the general procedures D, E and F (37% overall yield – yellow oil).

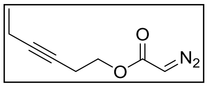

^{1}**H-NMR** (700 MHz, $CDCl_3$): δ 4.76 (br s, 1H), 4.21 (t, *J* = 7.0 Hz, 2H), 2.49 (tt, *J* = 7.0 Hz 2.1 Hz, 2H), 2.15 (qt, *J* = 7.0, 2.1 Hz, 2H), 2.15 (t, *J* = 7.0 Hz, 3H). ^{13}**C-NMR** (176 MHz, $CDCl_3$) δ 83.5, 74.6, 63.1, 19.4, 14.1, 12.3; **HRMS-ESI** (decomposes on ionization); **ATR-FTIR** (cm^{-1}): 3112, 2974, 2106 (diazo stretch), 1685, 1456, 1392, 1356, 1341, 1233, 1176, 1156, 999, 793, 778, 738.

Allyl 2-diazoacetate (**1c**)
1c was prepared by following the general procedures E and F (83% over 2 steps).

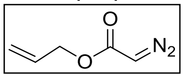

Spectroscopic data matches that previously reported. (Synlett **2014**, 25, 221 – 224)

Benzyl 2-diazoacetate (**1d**)
1d was purchased.

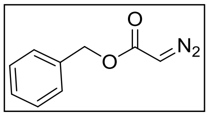

2-(trimethylsilyl)ethyl 2-diazoacetate (**1e**)
1e was prepared by following the general procedures D, E and G (51% over 3 steps).

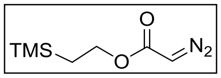

Spectroscopic data matches that previously reported. (Journal of Organic Chemistry **2017**, 82, 9291 – 9304)

Cholesteryl diazoacetate (**1f**)
1f was prepared by following the general procedures D, E and G (78% over 3 steps).

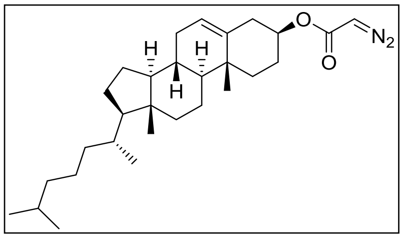

Spectroscopic data matches that previously reported. (Synthesis **2000**, 13,1936 – 1943)

Isobutyl 2-diazoacetate (**1g**)
1g was prepared by following the general procedures E and F (90% over 2 steps).

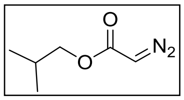

Spectroscopic data matches that previously reported. (Journal of Organic Chemistry **1995**, 60, 3035 – 3038)

Tert-butyl 2-diazoacetate (**1h**)
1h was prepared purchased.

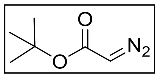

3-methylbut-2-en-1-yl 2-diazoacetate (**1i**)
1i was prepared by following the general procedures D, E and F.

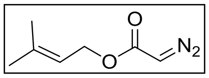

Spectroscopic data matches that previously reported. (Synlett **2014**, 25, 221 – 224)

2-((1*S*,5*R*)-6,6-dimethylbicyclo[3.1.1]hept-2-en-3-yl)ethyl 2-diazoacetate (**1j**)
1j was prepared by following the general procedures D, E and G (49% over 3 steps).

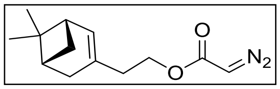

^{1}**H NMR** (400 MHz, CDCl$_3$) δ 5.29 (dq, *J* = 4.2, 1.4 Hz, 1H), 4.70 (s, 1H), 4.25 – 4.09 (m, 2H), 2.36 (dt, *J* = 8.5, 5.6 Hz, 1H), 2.33 – 2.26 (m, 2H), 2.25 – 2.14 (m, 2H), 2.10 – 2.02 (m, 2H), 1.27 (s, 3H), 1.14 (d, *J* = 8.5 Hz, 1H), 0.82 (s, 3H). ^{13}**C NMR** (101 MHz, CDCl$_3$) δ 144.2, 119.1, 63.3, 45.8, 40.9, 38.1, 36.2, 31.8, 31.5, 26.4, 21.2. **HRMS** (ESI) m/z calculated for [M+Na]$^+$ 217.1266 found 217.1254. **ATR-FTIR** (cm^{-1}): 2914, 2833, 2105, 1691, 1469, 1469, 1393, 1355, 1237, 1179, 1027, 958, 886, 789, 739.

Naphthalen-2-ylmethyl 2-diazoacetate (**1k**)
1k was prepared by following the general procedures D, E and F (37% over 3 steps – yellow oil)

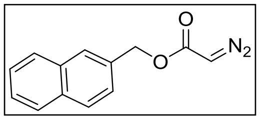

^{1}**H NMR** (700 MHz, CDCl3) δ 58.02 (d, *J* = 8.4 Hz, 1H), 7.89 (d, *J* = 8.4 Hz, 1H), 7.86 (d, *J* = 8.4 Hz, 1H), 7.58 – 7.52 (m, 3H), 7.46 (dd, *J* = 8.4, 7.0 Hz, 1H), 5.67 (s, 2H), 4.80 (br s, 1H). ^{13}**C NMR** (176 MHz, CDCl$_3$) δ 133.7, 131.6, 131.3, 129.4, 128.7, 127.5, 126.6, 126.0, 125.3, 123.5, 64.8. **HRMS** (ESI) m/z calculated for [M+Na]$^+$ 249.0634 found: 249.0631. **ATR-FTIR** (cm^{-1}): 3112, 2104 (diazo stretch), 1680, 1511, 1387, 1356, 1341, 1228, 1171, 1153, 996, 964, 792, 773, 735.

(*S*)-3,7-dimethyloct-6-en-1-yl 2-diazoacetate (**1l**)
1g was prepared by following the general procedures E and F (95% over 2 steps).

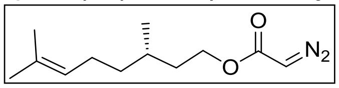

Spectroscopic data matches that previously reported. (Synthesis **2013**, 45, 903 – 912)

2-(dimethyl(oxo)-λ^6-sulfanylidene)-1-phenylethan-1-one (**2a**)
2a was prepared by following the general procedure A (75%).

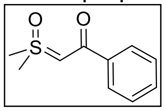

Spectroscopic data matches that previously reported. (Journal of Organic Chemistry **2016**, 81, 4158 – 4169)

2-(dimethyl(oxo)- λ^6-sulfanylidene)-1-(4-fluorophenyl)ethan-1-one (**2b**)
2b was prepared by following the general procedures A.

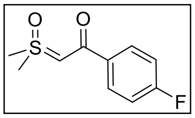

Spectroscopic data matches that previously reported. (Chemistry - A European Journal **2017**, 23, 16980 – 16984)

2-(dimethyl(oxo)- λ^6-sulfanylidene)-1-(3-chlorophenyl)ethan-1-one (**2c**)
2c was prepared by following the general procedures A (52% yield).

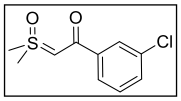

^{1}**H NMR** (600 MHz, CDCl$_3$) δ 7.78 (t, *J* = 1.8 Hz, 1H), 7.74 (dt, *J* = 7.8, 1.8 Hz, 1H), 7.41 – 7.39 (m, 1H), 7.32 (t, *J* = 7.8 Hz, 1H), 4.95 (s, 1H), 3.52 (s, 6H).. ^{13}**C NMR** (151 MHz, CDCl$_3$) δ 180.6, 140.7, 134.3, 130.6, 129.5, 126.9, 124.7, 68.8, 42.4. **HRMS** (ESI) m/z calculated for [M+H]$^+$ 231.0247 found: 231.0233. **ATR-FTIR** (cm^{-1}): 3089, 3068, 3013, 2922, 2855, 1703, 1580, 1535, 1425, 1375, 1303, 1177, 1069, 1026, 877, 856, 801, 740.

<u>2-(dimethyl(oxo)- λ⁶-sulfanylidene)-1-(3-fluorophenyl)ethan-1-one</u> (**2d**)

2d was prepared by following the general procedures A (73% yield).

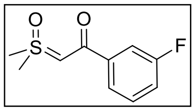

^{1}H NMR (600 MHz, $CDCl_3$) δ 7.57 – 7.54 (m, 1H), 7.50 (ddd, *J* = 9.8, 2.5, 1.6 Hz, 1H), 7.35 (td, *J* = 8.0, 5.7 Hz, 1H), 7.12 (tdd, *J* = 8.3, 2.6, 0.9 Hz, 1H), 4.95 (s, 1H), 3.52 (s, 6H). **^{13}C NMR** (151 MHz, $CDCl_3$) δ 180.6, 163.6, 141.3, 129.7, 122.2, 117.6, 113.6, 68.6, 42.4. **HRMS** (ESI) m/z calculated for $[M+H]^+$ 215.0542; found 215.0539. **ATR-FTIR** (cm^{-1}): 3082, 3018, 2920, 1532, 1444, 1391, 1244, 1177, 1038, 926, 868, 746.

<u>2-(dimethyl(oxo)- λ⁶-sulfanylidene)-1-(*m*-tolyl)ethan-1-one</u> (**2e**)

2e was prepared by following the general procedure A.

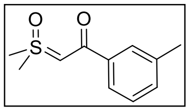

Spectroscopic data matches that previously reported. (Organic Letters **2018**, 20, 2464 – 2467)

<u>2-(dimethyl(oxo)- λ⁶-sulfanylidene)-1-(4-fluorophenyl)ethan-1-one</u> (**2f**)

2f was prepared by following the procedure A using the corresponding ester instead of the acyl chloride. (23%)

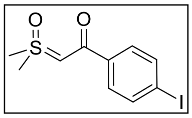

^{1}H NMR (600 MHz, $CDCl_3$) δ 7.73 (d, *J* = 8.4 Hz, 2H), 7.51 (d, *J* = 8.4 Hz, 2H), 4.94 (s, 1H), 3.50 (s, 6H). **^{13}C NMR** (151 MHz, $CDCl_3$) δ 181.3, 138.4, 137.5, 128.4, 97.7, 68.7, 42.6. **HRMS** (ESI) m/z calculated for $[M+Na]^+$ 344.9422 found 344.9414. **ATR-FTIR** (cm^{-1}): 3007, 2921, 1698, 1580, 1481, 1395, 1267, 1177, 1142, 1057, 1005, 950, 830, 787, 757.

<u>1-(dimethyl(oxo)- λ⁶-sulfanylidene)decan-2-one</u> (**2g**)

2g was prepared by following the general procedure A.

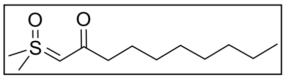

^{1}H NMR (400 MHz, $CDCl_3$) δ 4.34 (s, 1H), 3.37 (s, 6H), 2.22 – 2.08 (m, 2H), 1.61 – 1.50 (m, 2H), 1.38 – 1.19 (m, 10H), 0.86 (t, *J* = 6.9 Hz, 3H). **^{13}C NMR** (101 MHz, $CDCl_3$) δ 191.5, 68.79, 68.8, 42.5, 41.2, 32.0, 29.6, 29.3, 26.3, 22.7, 14.2. **HRMS** (ESI) m/z calculated for $[M+Na]^+$ 255.1395 found 255.1392. **ATR-FTIR** (cm^{-1}): 3389, 3063, 3026, 2957, 2920, 2852, 1554, 1466, 1391, 1309, 1174, 1032, 856, 761, 617.

<u>2-(dimethyl(oxo)- λ⁶-sulfanylidene)-1-(4-(trifluoromethyl)phenyl)ethan-1-one</u> (**2h**)

2h was prepared by following the general procedure A. (84%)

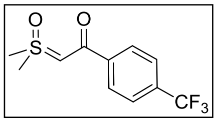

^{1}H NMR (600 MHz, $CDCl_3$) δ 7.88 (d, *J* = 8.4 Hz, 2H), 7.65 (d, *J* = 8.4 Hz, 2H), 5.00 (s, 1H), 3.53 (s, 6H). **^{13}C NMR** (151 MHz, $CDCl_3$) δ 180.5, 142.1, 126.9, 125.2 (q, *J* = 3.8 Hz), 62.2, 42.4. **HRMS** (ESI) m/z calculated for $[M+H]^+$ 265.0505 found 265.0503. **ATR-FTIR** (cm^{-1}): 3021, 2920, 1590, 1535, 1416, 1387, 1333, 1168, 1120, 1084, 1070, 1032, 1015, 903, 857, 767, 699.

<u>1-(4-(tert-butyl)phenyl)-2-(dimethyl(oxo)- λ⁶-sulfanylidene)ethan-1-one</u> (**2i**)

2i was prepared by following the general procedure A.

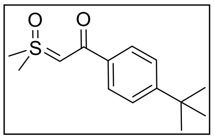

Spectroscopic data matches that previously reported. (Journal of Organic Chemistry **2016**, 81, 4158 – 416)

<u>1-([1,1'-biphenyl]-4-yl)-2-(dimethyl(oxo)- λ⁶-sulfanylidene)ethan-1-one</u> (**2j**)

2j was prepared by following the general procedure B. (50%)

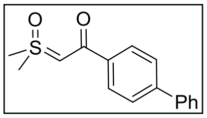

^{1}H NMR (400 MHz, $CDCl_3$) δ 7.91 – 7.84 (m, 2H), 7.65 – 7.59 (m, 4H), 7.48 – 7.41 (m, 2H), 7.40 – 7.33 (m, 1H), 5.02 (s, 1H), 3.53 (s, 7H). **^{13}C NMR** (151 MHz, $CDCl_3$) δ 182.0, 143.6, 140.54, 137.8, 129.0, 128.0, 127.3, 127.2, 127.0, 68.4, 42.7. **HRMS** (ESI) m/z calculated for $[M+Na]^+$ 295.0769 found 295.0762. **ATR-FTIR** (cm^{-1}): 3078, 3054, 3020, 2994, 2964, 1602, 1567, 1518, 1405, 1380, 1304, 1163, 1145, 1084, 1031, 1005, 993, 865, 846, 737, 690, 639, 568, 501, 443.

<u>1-(((dimethyl(oxo)- λ^6-sulfanylidene)methyl)sulfonyl)-4-methylbenzene</u> (**2k**)

2k was prepared by following the general procedure C. (41%)

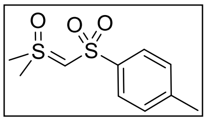

Spectroscopic data matches that previously reported. (Tetrahedron Letters **1966**, 3681 – 3687)

<u>Ethyl (*Z*)-4-oxo-4-phenylbut-2-enoate</u> (**3aa**)

3aa was prepared by following the general procedure H. (71% - *Z*/*E* = 9 : 1)

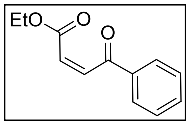

Spectroscopic data matches that previously reported. (European Journal of Organic Chemistry **2000**, 2795 – 280)

<u>Ethyl (*Z*)-4-(4-fluorophenyl)-4-oxobut-2-enoate</u> (**3ab**)

3ab was prepared by following the general procedure H. (74% - *Z*/*E* = 13 : 1)

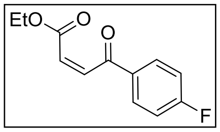

^{1}H NMR (700 MHz, $CDCl_3$) δ 7.98 – 7.95 (m, 2H), 7.16 – 7.13 (m, 2H), 6.84 (d, *J* = 11.9 Hz, 1H), 6.27 (d, *J* = 11.9 Hz, 1H), 4.05 (q, *J* = 7.0 Hz, 2H), 1.09 (t, *J* = 7.0 Hz, 2H). **^{13}C NMR** (100 MHz, $CDCl_3$) δ 192.6, 167.3, 164.8 (d, *J* = 14.1 Hz), 140.7, 132.4, 131.3 (d, *J* = 9.4 Hz), 126.1, 115.9 (d, *J* = 21.9 Hz), 61.1, 13.7. **HRMS** (ESI) m/z calculated for [M+Na]$^+$ 245.0584 found: 245.0579. **ATR-FTIR** (cm^{-1}): 2983, 1719, 1671, 1596, 1506, 1410, 1382, 1231, 1211, 1169, 1152, 1098, 1020, 1010, 828, 815.

<u>Ethyl (*Z*)-4-(3-chlorophenyl)-4-oxobut-2-enoate</u> (**3ac**)

3ac was prepared by following the general procedure H. (78% - *Z*/*E* = 10 : 1)

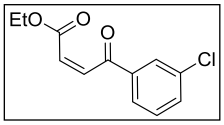

^{1}H NMR (400 MHz, $CDCl_3$) δ 7.84 (t, *J* = 1.6 Hz, 1H), 7.74 (dt, *J* = 6.4, 1.6 Hz, 1H), 7.50 – 7.47 (m, 1H), 7.35 (t, *J* = 4.0 Hz, 1H), 6.76 (d, *J* = 12.0 Hz, 1H), 6.23 (d, *J* = 12.0 Hz, 1H), 4.00 (q, *J* = 7.2 Hz, 2H), 1.04 (t, *J* = 7.2 Hz, 3H). **^{13}C NMR** (176 MHz, $CDCl_3$) δ 193.0, 164.6, 140.5, 137.4, 135.1, 133.6, 130.7, 128.7, 126.8, 126.6, 61.3, 13.7. **HRMS** (ESI) m/z calculated for [M+Na]$^+$ 261.0289 found: 261.0293. **ATR-FTIR** (cm^{-1}): 2983, 2926, 1719, 1675, 1571, 1470, 1424, 1382, 1287, 1208, 1162, 1096, 1075, 1022, 951, 844, 788, 768.

<u>Ethyl (*Z*)-4-(3-fluorophenyl)-4-oxobut-2-enoate</u> (**3ad**)

3ad was prepared by following the general procedure H. (62% - *Z*/*E* = 4 : 1)

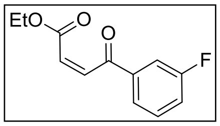

^{1}H NMR (600 MHz, $CDCl_3$) δ 7.84 (dt, *J* = 6.0, 1.2 Hz, 1H), 7.65 – 7.63 (m, 1H), 7.48 – 7.44 (m, 1H), 7.28 (ddd, *J* = 7.8, 2.4, 0.6, 1H), 6.84 (d, *J* = 12.0 Hz, 1H), 6.30 (d, *J* = 12.0 Hz, 1H), 4.06 (q, *J* = 7.2 Hz, 2H), 1.10 (t, *J* = 7.2 Hz, 3H). **^{13}C NMR** (151 MHz, $CDCl_3$) δ 193.0, 164.6, 140.5, 135.7, 130.4 (d, *J* = 7.7 Hz), 126.5, 124.6 (d, *J* = 3.0 Hz), 120.6 (d, *J* = 21.6 Hz), 115.2 (d, *J* = 21.6 Hz), 61.5, 13.7. **HRMS** (ESI) m/z calculated for [M+Na]$^+$ 245.0590 found: 245.0582. **ATR-FTIR** (cm^{-1}): 2992, 2925, 2189, 2170, 1723, 1679, 1589, 1484, 1444, 1383, 1297, 1264, 1218, 1164, 1149, 1025, 872, 832, 804, 784. *Product contains minor amounts of inseparable homo-coupling product.*

<u>Ethyl (*Z*)-4-oxo-4-(m-tolyl)but-2-enoate</u> (**3ae**)

3ae was prepared by following the general procedure H. (62% - *Z*/*E* = 4 : 1)

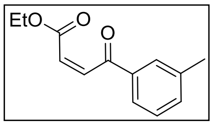

^{1}H NMR (400 MHz, $CDCl_3$) δ 7.77 (d, *J* = 1.3 Hz, 1H), 7.74 – 7.70 (m, 1H), 7.42 – 7.32 (m, 2H), 6.87 (d, *J* = 12.2 Hz, 1H), 6.26 (d, *J* = 12.2 Hz, 1H), 4.05 (q, *J* = 7.1 Hz, 2H), 1.08 (t, *J* = 7.1 Hz, 3H). **^{13}C NMR** (101 MHz, $CDCl_3$) δ 194.4, 165.0, 141.3, 138.7, 136.1, 134.6, 129.2, 128.7, 126.3, 126.1, 61.2, 21.5, 13.9. **HRMS** (ESI) m/z calculated for [M+Na]$^+$ 241.0841 found 241.0835. **ATR-FTIR** (cm^{-1}): 3039, 2982, 2929, 2908, 2869, 1719, 1670, 1602, 1585, 1396, 1382, 1286, 1264, 1208, 1154, 1022, 952, 832, 773, 694, 668, 550, 457.

Ethyl (*E*)-4-oxo-4-(m-tolyl)but-2-enoate ((*E*)-**3ae**)

(*E*)-**3ae** was prepared by following the general procedure I. (>95% - *Z*/*E* = 1 : 99)

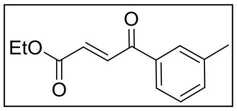

^{1}H NMR (600 MHz, $CDCl_3$) δ 7.90 (d, *J* = 15.6 Hz, 1H), 7.80 (d, *J* = 12.5 Hz, 2H), 7.43 (d, *J* = 7.5 Hz, 1H), 7.40 (t, *J* = 7.5 Hz, 1H), 6.88 (d, *J* = 15.6 Hz, 1H), 4.31 (q, *J* = 7.1 Hz, 2H), 2.44 (s, 3H), 1.35 (t, *J* = 7.1 Hz, 3H). **^{13}C NMR** (151 MHz, $CDCl_3$) δ 189.9, 165.8, 139.0, 136.8, 136.8, 134.8, 132.6, 129.5, 129.0, 128.9, 126.3, 61.5, 21.5, 14.3. **HRMS** (ESI) m/z calculated for $[M+Na]^+$ 241.0841 found 241.0833.

ATR-FTIR (cm^{-1}): 2983, 2929, 2359, 2179, 1723, 1673, 1604, 1586, 1447, 1383, 1296, 1266, 1214, 1159, 1095, 1027, 782, 615, 590.

Ethyl (*Z*)-4-(4-iodophenyl)-4-oxobut-2-enoate (**3af**)

3af was prepared by following the general procedure H. (59% - *Z*/*E* = 5 : 1)

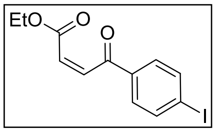

^{1}H NMR (400 MHz, $CDCl_3$) δ 7.87 – 7.83 (m, 2H), 7.67 – 7.62 (m, 2H), 6.82 (d, *J* = 12.1 Hz, 1H), 6.28 (d, *J* = 12.1 Hz, 1H), 4.06 (q, *J* = 7.1 Hz, 2H), 1.11 (t, *J* = 7.1 Hz, 3H). **^{13}C NMR** (151 MHz, $CDCl_3$) δ 193.7, 164.8, 140.7, 138.2, 135.3, 130.1, 126.5, 102.0, 61.4, 29.9, 13.9. **HRMS** (ESI) m/z calculated for $[M+Na]^+$ 352.9651 found 352.9641. **ATR-FTIR** (cm^{-1}): 3078, 3054, 3020, 2994, 2964, 2927, 2915, 2890, 1602, 1567, 1518, 1405, 1380, 1304, 1163, 1145, 1084, 1031, 1005, 993, 865, 846, 737, 690, 639, 568, 501, 443.

Ethyl (*Z*)-4-oxododec-2-enoate (**3ag**)

3ag was prepared by following the general procedure H. (54% - *Z*/*E* = 3 : 2)

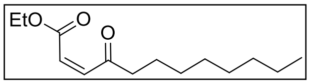

^{1}H NMR (400 MHz, $CDCl_3$) δ 6.47 (d, *J* = 12.1 Hz, 1H), 6.00 (d, *J* = 12.1 Hz, 1H), 4.21 (q, *J* = 7.1 Hz, 2H), 2.59 (dd, *J* = 9.6, 5.3 Hz, 2H), 1.70 – 1.59 (m, 2H), 1.37 – 1.22 (m, 13H), 0.88 (t, *J* = 6.9 Hz, 3H). **^{13}C NMR** (101 MHz, $CDCl_3$) δ 204.0, 166.0, 141.7, 124.9, 61.3, 42.9, 32.0, 29.5, 29.3, 29.3, 23.5, 22.8, 14.2, 14.2. **HRMS** (ESI) m/z calculated for $[M+Na]^+$ 263.1623 found 263.1616. **ATR-FTIR** (cm^{-1}): 3443, 3334, 3059, 3028, 2982, 2913, 2832, 1719, 1670, 1598, 1581, 1448, 1396, 1382, 1286, 1200, 1161, 1004, 810, 755, 729, 704, 688, 543.

Ethyl (*E*)-4-oxododec-2-enoate ((*E*)-**3ag**)

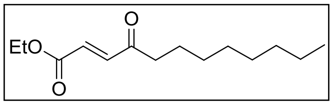

^{1}H NMR (400 MHz, $CDCl_3$) δ 7.06 (d, *J* = 16.0 Hz, 1H), 6.66 (d, *J* = 16.0 Hz, 1H), 4.27 (q, *J* = 7.1 Hz, 2H), 2.62 (t, *J* = 7.4 Hz, 2H), 1.69 – 1.59 (m, 2H), 1.31 – 1.23 (m, 13H), 0.90 – 0.82 (m, 3H). **^{13}C NMR** (151 MHz, $CDCl_3$) δ 200, 165.8, 139.5, 130.8, 61.5, 41.7, 31.9, 29.5, 29.3, 29.3, 23.9, 22.8, 14.3, 14.2. **HRMS** (ESI) m/z calculated for $[M+Na]^+$ 263.1623 found 263.1618. **ATR-FTIR** (cm^{-1}): 2924, 2855, 1727, 1702, 1464, 1369, 1278, 1181, 1031, 981, 750.

Ethyl (*Z*)-4-oxo-4-(4-(trifluoromethyl)phenyl)but-2-enoate (**3ah**)

3ah was prepared by following the general procedure H. (54% - *Z*/*E* = 13 : 1)

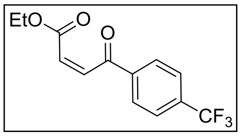

^{1}H NMR (600 MHz, $CDCl_3$) δ 8.05 (d, *J* = 8.1 Hz, 2H), 7.74 (d, *J* = 8.2 Hz, 2H), 6.89 – 6.83 (m, 1H), 6.36 – 6.30 (m, 1H), 4.06 (q, *J* = 7.1 Hz, 2H), 1.11 (t, *J* = 7.1 Hz, 3H). **^{13}C NMR** (151 MHz, $CDCl_3$) δ 193.3, 164.6, 140.5, 138.5, 134.7 (q, *J* = 32.6 Hz), 129.0, 126.8, 125.8 (q, *J* = 3.6 Hz), 61.3, 13.8. **HRMS** (ESI) m/z calculated for $[M+H]^+$ 273.0738 found: 273.0729. **ATR-FTIR** (cm^{-1}): 2986, 1720, 1680, 1412, 1383, 1323, 1214, 1164, 1125, 1107, 1065, 1024, 1010, 947, 858, 825, 781, 705. *Product contains minor amounts of inseparable homo-coupling product.*

Ethyl (*Z*)-4-(4-(tert-butyl)phenyl)-4-oxobut-2-enoate (**3ai**)

3ai was prepared by following the general procedure H. (62% - *Z*/*E* = 4 : 1)

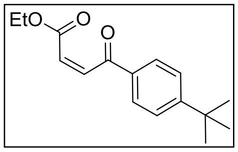

Spectroscopic data matches that previously reported. (Bioorganic and Medicinal Chemistry **2013**, *21*, 7830 – 7840)

Ethyl (*Z*)-4-([1,1'-biphenyl]-4-yl)-4-oxobut-2-enoate (**3aj**)

3aj was prepared by following the general procedure H. (70% - *Z*/*E* = 3 : 2)

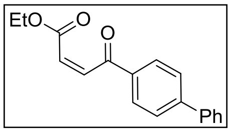

^{1}H NMR (400 MHz, $CDCl_3$) δ 8.05 – 7.99 (m, 2H), 7.74 – 7.68 (m, 2H), 7.68 – 7.61 (m, 2H), 7.51 – 7.45 (m, 2H), 7.44 – 7.38 (m, 1H), 6.91 (d, *J* = 12.2 Hz, 1H), 6.30 (d, *J* = 12.2 Hz, 1H), 4.07 (q, *J* = 7.1 Hz, 2H), 1.10 (t, *J* = 7.1 Hz, 3H). **^{13}C NMR** (176 MHz, $CDCl_3$) δ 194.0, 165.0, 146.5, 141.3, 140.0, 134.7, 129.5, 129.1, 128.5, 127.6, 127.5, 126.2, 61.3, 13.9. **HRMS** (ESI) m/z calculated for $[M+Na]^+$ 303.0997 found 303.0987. **ATR-FTIR** (cm^{-1}): 3055, 3032, 2977, 2956, 2937, 2902, 2873, 2853, 1715, 1664, 1602, 1560, 1400, 1382, 1363, 1252, 1218, 1167, 1022, 1005, 942, 823, 756, 691, 559, 499, 457.

Ethyl (*E*)-4-([1,1'-biphenyl]-4-yl)-4-oxobut-2-enoate ((*E*)-**3aj**)

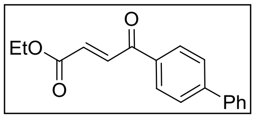

Spectroscopic data matches that previously reported. (RSC Advances **2017**, *7*, 4763 – 4775)

Ethyl (Z)-3-tosylacrylate (**3ak**)

3ak was prepared by following the general procedure H. (66% - *Z*/*E* = 10 : 1)

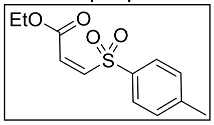

Spectroscopic data matches that previously reported. (Tetrahedron **2016**, 72, 7895 – 7900)

Hex-3-yn-1-yl (Z)-4-oxo-4-phenylbut-2-enoate (**3ba**)

3ba was prepared by following the general procedure H. (67% - *Z*/*E* = 9 : 1)

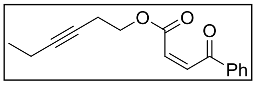

^{1}H NMR (700 MHz, $CDCl_3$) δ 7.93 (d, *J* = 7.0 Hz, 2H), 7.59 (t, *J* = 7.0 Hz, 1H), 7.48 (t, *J* = 7.0 Hz, 2H), 6.90 (d, *J* = 11.9 Hz, 1H), 6.30 (d, *J* = 11.9 Hz, 1H), 4.05 (t, *J* = 7.0 Hz, 1H), 2.30 – 2.27 (m, 2H), 2.12 – 2.08 (m, 2H), 1.07 (t, *J* = 7.0 Hz, 1H). **^{13}C NMR** (176 MHz, $CDCl_3$) δ 194.0, 164.5, 141.5, 135.7, 133.7, 128.7, 125.7, 83.5, 74.3, 63.4, 18.8, 14.0, 12.3. **HRMS** (ESI) m/z calculated for $[M+H]^+$ 279.0997 found 279.0993. **ATR-FTIR** (cm^{-1}): 2974, 1723, 1672, 1597, 1581, 1449, 1397, 1372, 1201, 1163, 1069, 1005, 812, 757, 729.

Allyl (Z)-4-oxo-4-phenylbut-2-enoate (**3ca**)

3ca was prepared by following the general procedure H. (75% - *Z*/*E* = 11 : 1)

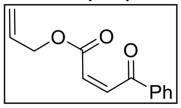

^{1}H NMR (400 MHz, $CDCl_3$) δ 7.98 – 7.88 (m, 2H), 7.63 – 7.54 (m, 1H), 7.52 – 7.44 (m, 2H), 6.91 (d, *J* = 12.2 Hz, 1H), 6.31 (d, *J* = 12.2 Hz, 1H), 5.82 – 5.64 (m, 1H), 5.23 – 5.11 (m, 2H), 4.50 (t, *J* = 1.3 Hz, 1H), 4.48 (t, *J* = 1.3 Hz, 1H). **^{13}C NMR** (101 MHz, $CDCl_3$) δ 194.1, 164.6, 141.5, 136.0, 133.8, 131.5, 128.9, 128.9, 125.9, 118.9, 65.9. **HRMS** (ESI) m/z calculated for $[M+H]^+$ 217.0865 found 217.0860. **ATR-FTIR** (cm^{-1}): 3084, 3059, 3028, 2982, 2944, 2886, 1719, 1670, 1598, 1581, 1448, 1391, 1235, 1200, 1161, 983, 929, 810, 755, 729, 704, 488, 543.

Benzyl (Z)-4-oxo-4-phenylbut-2-enoate (**3da**)

3da was prepared by following the general procedure H. (63% - *Z*/*E* = 9 : 1)

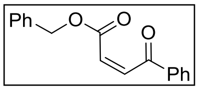

^{1}H NMR (700 MHz, $CDCl_3$) δ 7.90 (d, *J* = 7.7 Hz, 2H), 7.58 (t, *J* = 7.7 Hz, 1H), 7.45 (t, *J* = 7.7 Hz, 2H), 7.29 – 7.28 (m, 3H), 7.17 (t, *J* = 3.5 Hz, 2H), 6.92 (d, *J* = 12.2 Hz, 1H), 6.31 (d, *J* = 12.2 Hz, 1H), 5.03 (s, 2H). **^{13}C NMR** (101 MHz, $CDCl_3$) δ 194.0, 164.6, 141.6, 135.8, 135.0, 133.6, 128.8, 128.7, 128.5, 128.4, 128.3, 125.7, 67.0. **HRMS** (ESI) m/z calculated for $[M+Na]^+$ 289.0835 found: 289.0840. **ATR-FTIR** (cm^{-1}): 3031, 1717, 1668, 1596, 1579, 1449, 1398, 1361, 1204, 1167, 1016, 961, 824, 759, 738.

Benzyl (Z)-4-oxo-4-phenylbut-2-enoate (**3ea**)

3ea was prepared by following the general procedure H. (51% - *Z*/*E* = 5 : 1)

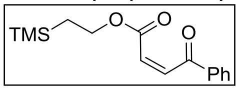

^{1}H NMR (400 MHz, $CDCl_3$) δ 7.99 – 7.91 (m, 2H), 7.58 (ddd, *J* = 8.7, 4.7, 2.0 Hz, 1H), 7.52 – 7.41 (m, 2H), 6.86 (d, *J* = 12.1 Hz, 1H), 6.27 (d, *J* = 12.1 Hz, 1H), 4.11 – 4.04 (m, 2H), 0.86 – 0.75 (m, 2H), -0.03 (s, 9H). **^{13}C NMR** (101 MHz, $CDCl_3$) δ 194.3, 165.1, 140.9, 136.1, 133.7, 128.9, 128.8, 126.5, 63.6, 17.1, -1.5. **HRMS** (ESI) m/z calculated for $[M+Na]^+$ 299.1079 found 299.1077.

ATR-FTIR (cm^{-1}): 3442, 3334, 3084, 3059, 3028, 2953, 2898, 1719, 1670, 1598, 1581, 1448, 1391, 1248, 1218, 1200, 1161, 1039, 1015, 967, 934, 834, 755, 729, 704, 688, 547.

Benzyl (*E*)-4-oxo-4-phenylbut-2-enoate (**3ea**)
3ea was prepared by following the general procedure I. (>95% - *Z*/*E* = 1 : 99)

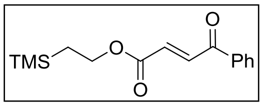

^{1}H NMR (400 MHz, $CDCl_3$) δ 8.00 (dt, *J* = 8.5, 1.6 Hz, 2H), 7.90 (d, *J* = 15.6 Hz, 1H), 7.66 – 7.57 (m, 1H), 7.55 – 7.49 (m, 2H), 6.87 (d, *J* = 15.6 Hz, 1H), 4.38 – 4.29 (m, 2H), 1.12 – 1.05 (m, 2H), 0.08 (s, 9H). **^{13}C NMR** (101 MHz, $CDCl_3$) δ 189.8, 165.9, 136.8, 136.4, 134.0, 133.0, 129.0, 129.0, 63.9, 17.5, -1.3. **HRMS** (ESI) m/z calculated for $[M+Na]^+$ 299.1079 found 299.1081. **ATR-FTIR** (cm^{-1}): 2954, 1722, 1675, 1598, 1450, 1396, 1250, 1220, 1166, 1063, 1041, 1016, 937, 860, 838, 758, 691.

(3*S*,8*S*,9*S*,10*R*,13*R*,14*S*,17*R*)-10,13-dimethyl-17-((*R*)-6-methylheptan-2-yl)-2,3,4,7,8,9,10,11,12,13,14,15,16,17-tetradecahydro-1H-cyclopenta[a]phenanthren-3-yl (*Z*)-4-oxo-4-phenylbut-2-enoate (**3fa**)
3fa was prepared by following the general procedure H. (65% - *Z*/*E* = 5 : 1)

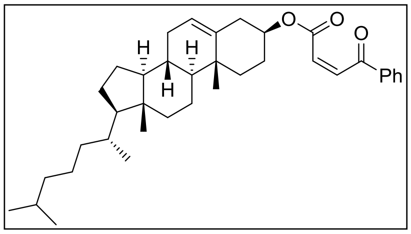

^{1}H NMR (400 MHz, $CDCl_3$) δ 7.89 – 7.83 (m, 2H), 7.52 – 7.44 (m, 1H), 7.43 – 7.33 (m, 2H), 6.75 (d, *J* = 12.2 Hz, 1H), 6.15 (d, *J* = 12.2 Hz, 1H), 5.19 – 5.13 (m, 1H), 4.45 – 4.36 (m, 1H), 2.01 (ddd, *J* = 13.0, 5.0, 2.2 Hz, 1H), 1.94 – 1.78 (m, 3H), 1.78 – 1.69 (m, 1H), 1.69 – 1.61 (m, 1H), 1.59 – 1.51 (m, 1H), 1.46 – 0.84 (m, 21H), 0.83 – 0.72 (m, 12H), 0.55 (s, 3H). **^{13}C NMR** (101 MHz, $CDCl_3$) δ 194.3, 164.2, 140.7, 139.5, 136.2, 133.8, 129.0, 128.9, 126.6, 122.9, 75.3, 56.8, 56.3, 50.1, 42.4, 39.8, 39.7, 37.6, 36.9, 36.6, 36.3, 35.9, 32.0, 32.0, 28.6, 28.2, 27.3, 24.4, 24.0 23.0, 21.0, 21.1, 19.3, 18.9, 12.0. **HRMS** (ESI) m/z calculated for $[M+Na]^+$ 567.3795 found 567.3814. **ATR-FTIR** (cm^{-1}): 2935, 2903, 2889, 2866, 2850, 1719, 1670, 1598, 1581, 1448, 1396, 1375, 1310, 1286, 1212, 1168, 1162, 1039, 1004, 958, 755, 729, 704, 688, 546.

Isobutyl (*Z*)-4-oxo-4-phenylbut-2-enoate (**3ga**)
3ga was prepared by following the general procedure H. (40% - *Z*/*E* = 4 : 1)

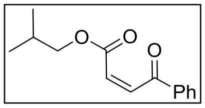

^{1}H NMR (400 MHz, $CDCl_3$) δ 7.98 – 7.91 (m, 2H), 7.63 – 7.55 (m, 1H), 7.51 – 7.42 (m, 2H), 6.88 (d, *J* = 12.2 Hz, 1H), 6.28 (d, *J* = 12.2 Hz, 1H), 3.77 (d, *J* = 6.6 Hz, 2H), 1.86 – 1.66 (m, 1H), 0.77 (d, *J* = 6.7 Hz, 6H). **^{13}C NMR** (101 MHz, $CDCl_3$) δ 194.2, 165.0, 141.2, 135.9, 133.8, 129.0, 128.9, 126.1, 71.5, 27.6, 19.1. **HRMS** (ESI) m/z calculated for $[M+H]^+$ 233.1178 found 233.1173. **ATR-FTIR** (cm^{-1}): 3084, 3059, 3028, 2961, 2935, 2894, 2875, 1719, 1670, 1598, 1581, 1469, 1448, 1401, 1385, 1369, 1285, 1208, 1161, 1017, 993, 810, 755, 729, 704, 688, 529.

Isobutyl (*E*)-4-oxo-4-phenylbut-2-enoate ((*E*)-**3ga**)
3ga was prepared by following the general procedure I. (>95% - *Z*/*E* = 1 : 99).

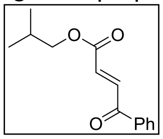

Spectroscopic data matches that previously reported. (Tetrahedron **2014**, *70*, 5834 – 5842)

Tert-butyl (*Z*)-4-oxo-4-phenylbut-2-enoate (**3ha**)
3ha was prepared by following the general procedure H. (51% - *Z*/*E* = 9 : 1)

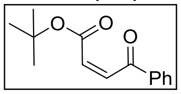

^{1}H NMR (700 MHz, $CDCl_3$) δ 7.96 – 7.95 (m, 2H), 7.59 (t, *J* = 7.7 Hz, 1H), 7.48 (t, *J* = 7.7 Hz, 2H), 6.75 (d, *J* = 12.6 Hz, 1H), 6.19 (d, *J* = 12.6 Hz, 1H), 1.22 (s, 9H). **^{13}C NMR** (151 MHz, $CDCl_3$) δ 194.1, 163.9, 139.2, 136.0, 135.9, 133.6, 129.0, 128.7, 127.9, 82.2, 27.6. **HRMS** (ESI) m/z calculated for $[M+Na]^+$ 255.0992 found: 255.0997. **ATR-FTIR** (cm^{-1}): 3004, 2933, 1719, 1674, 1598, 1479, 1449, 1393, 1368, 1299, 1229, 1151, 1013, 952, 837, 689.

3-methylbut-2-en-1-yl (*Z*)-4-oxo-4-phenylbut-2-enoate (**3ia**)

3ia was prepared by following the general procedure H. (59% - *Z*/*E* = 5 : 1)

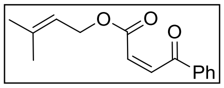

^{1}H NMR (600 MHz, $CDCl_3$) δ 7.94 – 7.93 (m, 2H), 7.60 – 7.57 (m, 1H), 7.47 (t, *J* = 7.2 Hz, 2H), 6.87 (d, *J* = 12.0 Hz, 1H), 6.28 (d, *J* = 12.0 Hz, 1H), 5.13 – 5.10 (m, 1H), 4.48 (d *J* = 7.8 Hz, 2H), 1.67 (s, 3H), 1.57 (s, 3H). **^{13}C NMR** (151 MHz, $CDCl_3$) δ 194.1, 164.8, 141.0, 139.7, 135.9, 133.6, 128.8, 128.7, 126.0, 117.7, 61.9, 25.7, 17.9. **HRMS** (ESI) m/z calculated for $[M+Na]^+$ 267.0997 found 267.0988. **ATR-FTIR** (cm^{-1}): 2919, 1719, 1672, 1598, 1581, 1448, 1395, 1281, 1208, 1163, 1034, 1015, 943, 813, 755.

2-((1*S*,5*R*)-6,6-dimethylbicyclo[3.1.1]hept-2-en-3-yl)ethyl (*Z*)-4-oxo-4-phenylbut-2-enoate (**3ja**)

3ja was prepared by following the general procedure H. (62% - *Z*/*E* = 10 : 1)

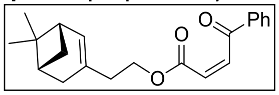

^{1}H NMR (400 MHz, $CDCl_3$) δ 7.94 (t, *J* = 5.7 Hz, 2H), 7.62 – 7.54 (m, 1H), 7.51 – 7.44 (m, 2H), 6.87 (d, *J* = 12.1 Hz, 1H), 6.26 (d, *J* = 12.1 Hz, 1H), 5.20 – 5.11 (m, 1H), 4.05 – 3.95 (m, 2H), 2.34 – 2.27 (m, 1H), 2.24 – 2.16 (m, 1H), 2.16 – 2.08 (m, 3H), 2.07 – 2.02 (m, 1H), 1.93 (td, *J* = 5.6, 1.5 Hz, 1H), 1.23 (s, 3H), 1.07 (d, *J* = 8.6 Hz, 1H), 0.76 (s, 3H). **^{13}C NMR** (101 MHz, $CDCl_3$) δ 194.2, 164.9, 143.8, 141.3, 136.0, 133.8, 128.9, 128.9, 126.1, 119.0, 63.5, 45.7, 40.8, 38.1, 35.6, 31.7, 31.4, 26.4, 21.2. **HRMS** (ESI) m/z calculated for $[M+Na]^+$ 347.1623 found 347.1617. **ATR-FTIR** (cm^{-1}): 3442, 3334, 3059, 3028, 2982, 2913, 2832, 1719, 1670, 1598, 1581, 1448, 1396, 1382, 1286, 1200, 1161, 1004, 810, 755, 729, 704, 688, 543.

Naphthalen-2-ylmethyl (*Z*)-4-oxo-4-phenylbut-2-enoate (**3ka**)

3ka was prepared by following the general procedure H. (65% - *Z*/*E* = 6 : 1)

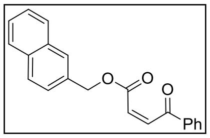

^{1}H NMR (600 MHz, $CDCl_3$) δ 7.86 – 7.81 (m, 5H), 7.51 – 7.48 (m, 4H), 7.39 – 7.34 (m, 3H), 6.90 (d, *J* = 12.0 Hz, 1H), 6.31 (d, *J* = 12.0 Hz, 1H), 5.50 (s, 2H).**^{13}C NMR** (151 MHz, $CDCl_3$) δ 193.9, 164.7, 141.6, 135.6, 133.6, 131.6, 130.5, 129.5, 128.7, 128.6, 127.8, 126.6, 125.9, 125.7, 125.2, 123.5, 65.2. **HRMS** (ESI) m/z calculated for $[M+Na]^+$ 339.0997 found 339.0993. **ATR-FTIR** (cm^{-1}): 3051, 1720, 1670, 1597, 1511, 1448, 1395, 1234, 1197, 1158, 1080, 1061, 1033, 1014, 955, 858, 793, 775, 734. *Product contains minor amounts of inseparable homo-coupling product.*

(*S*)-3,7-dimethyloct-6-en-1-yl (*Z*)-4-oxo-4-phenylbut-2-enoate (**3la**)

3la was prepared by following the general procedure H. (53% - *Z*/*E* = 8 : 1)

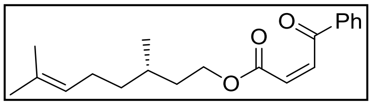

^{1}H NMR (400 MHz, $CDCl_3$) δ 7.98 – 7.91 (m, 2H), 7.58 (ddd, *J* = 8.7, 4.7, 2.0 Hz, 1H), 7.51 – 7.44 (m, 2H), 6.87 (d, *J* = 12.2 Hz, 1H), 6.27 (d, *J* = 12.2 Hz, 1H), 5.09 – 5.00 (m, 1H), 4.09 – 3.94 (m, 2H), 1.97 – 1.80 (m, 2H), 1.68 (d, *J* = 1.0 Hz, 3H), 1.58 (s, 3H), 1.50 – 1.41 (m, 1H), 1.40 – 1.31 (m, 1H), 1.27 – 1.18 (m, 2H), 1.13 – 1.01 (m, 1H), 0.79 (d, *J* = 6.6 Hz, 3H).**^{13}C NMR** (101 MHz, $CDCl_3$) δ 194.2, 165.0, 141.1, 136.00, 133.8, 131.4, 128.9, 128.9, 126.2, 124.7, 77.5, 77.2, 76.8, 63.8, 37.0, 35.1, 29.4, 25.8, 25.4, 19.3, 17.8. **HRMS** (ESI) m/z calculated for $[M+Na]^+$ 337.1780 found 337.1765. **ATR-FTIR** (cm^{-1}): 3028, 2960, 2912, 2872, 2853, 1719, 1670, 1598, 1581, 1448, 1399, 1375, 1285, 1230, 1205, 1163, 1015, 813, 755, 729, 704, 688, 547.

V. Spectra

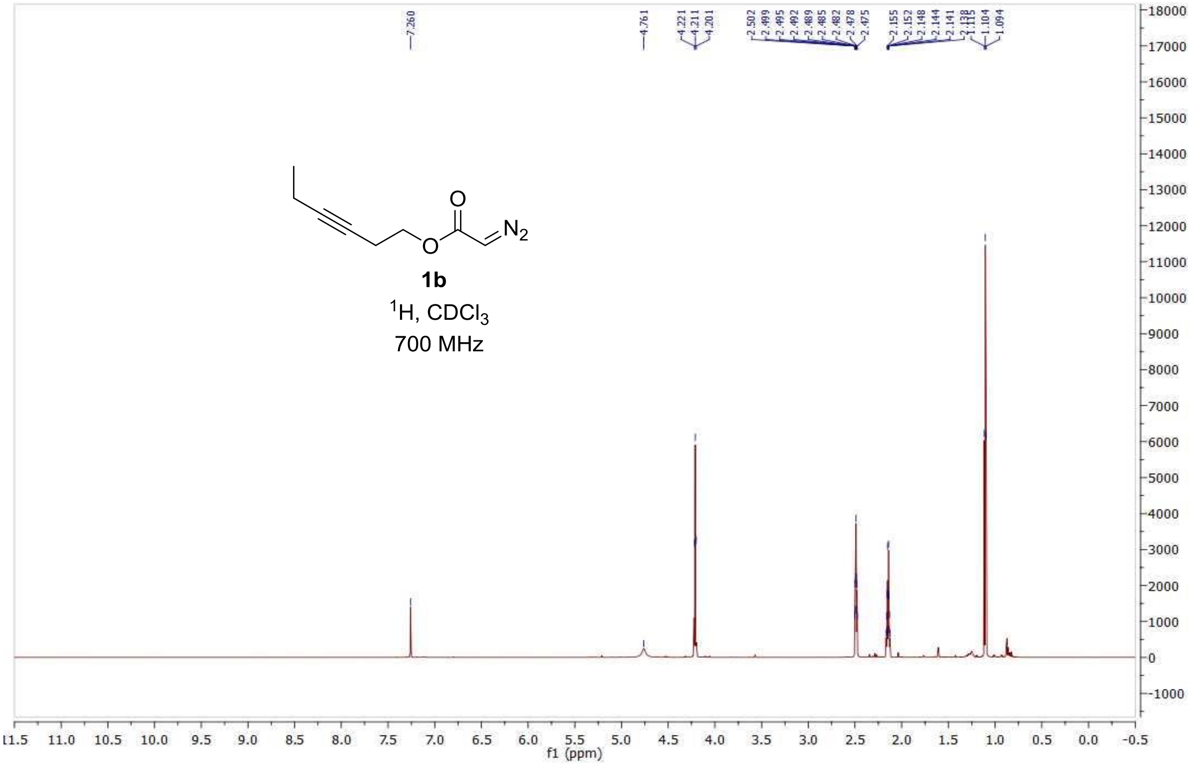
7.260
4.761
4.221
4.211
4.201
2.502
2.499
2.495
2.492
2.489
2.485
2.482
2.478
2.475
2.155
2.152
2.148
2.144
2.141
1.104
1.094
O
O
N_2
1b
^1H, $CDCl_3$
700 MHz
f1 (ppm)

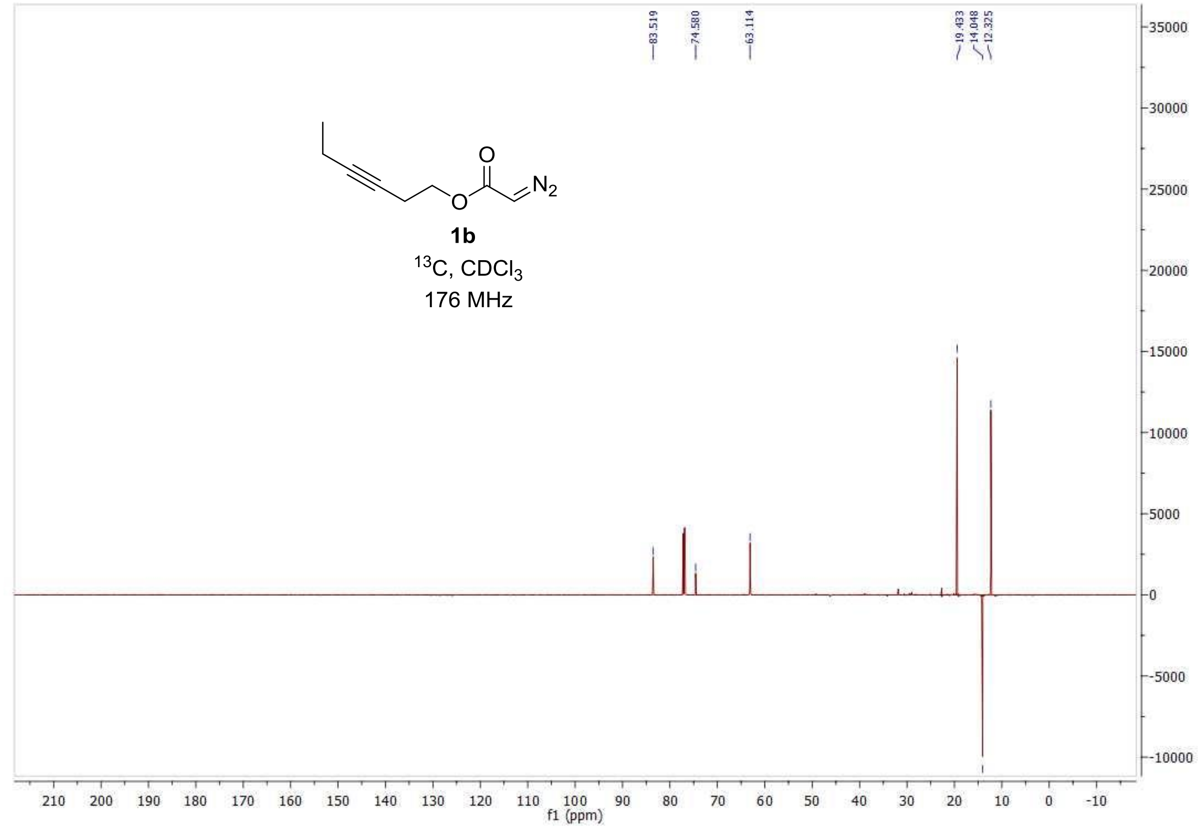
83.519
74.580
63.114
19.433
14.048
12.325
O
O
N_2
1b
^{13}C, $CDCl_3$
176 MHz
f1 (ppm)

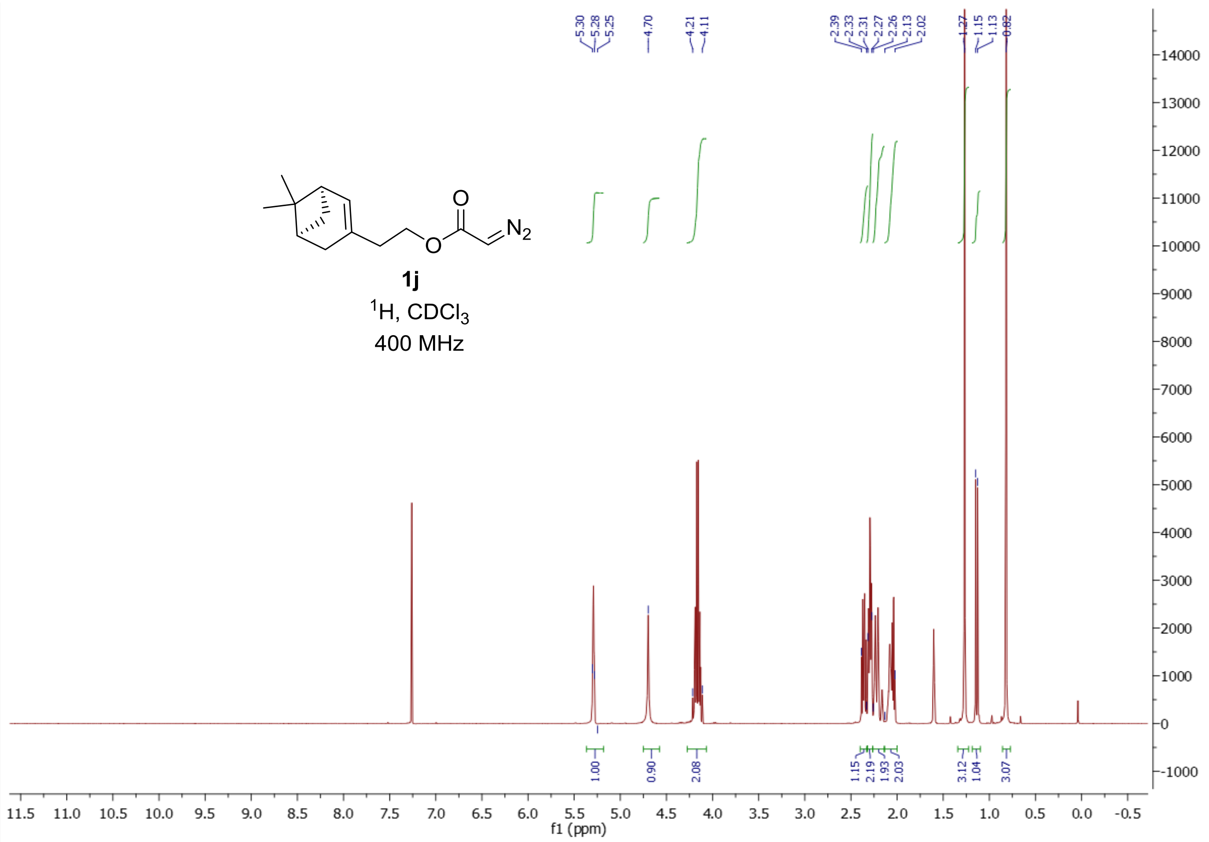
O
N2
O
1j
1H, CDCl3
400 MHz
f1 (ppm)

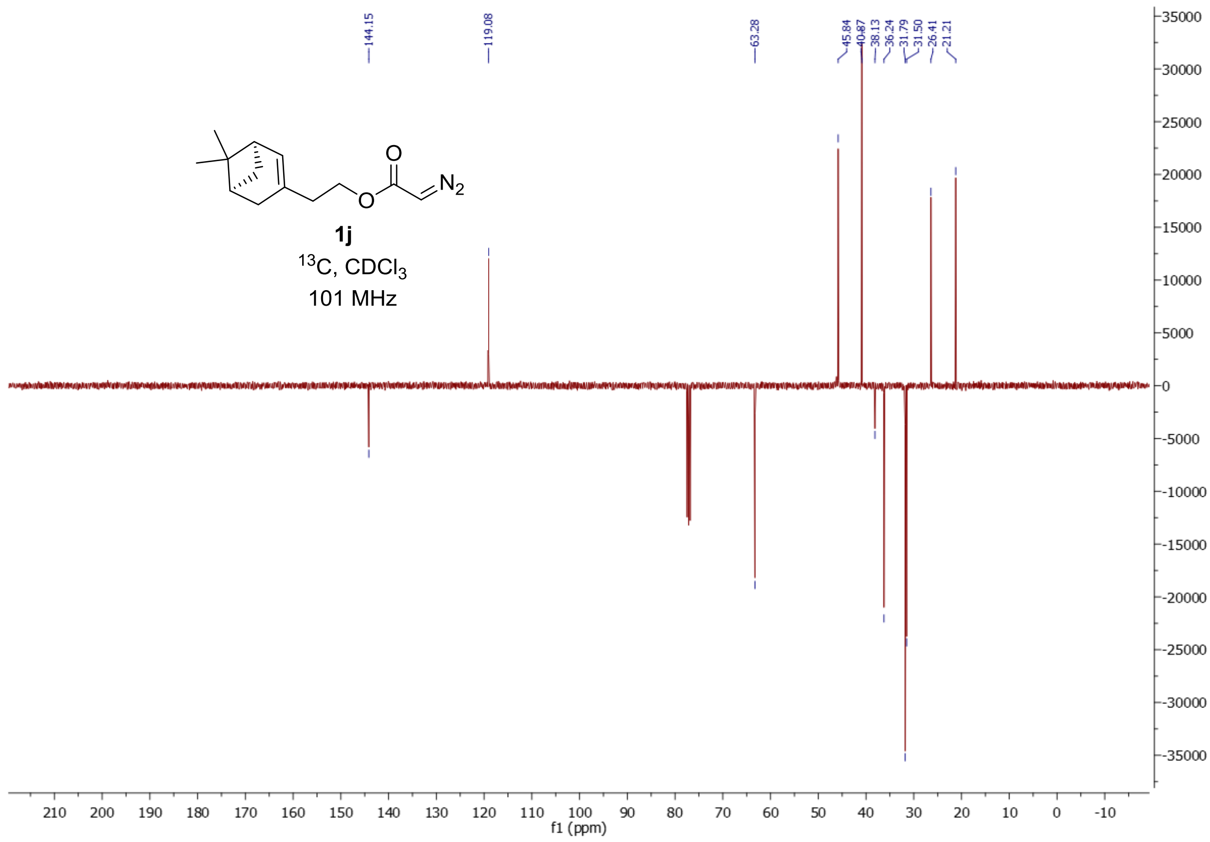
O
N2
O
1j
13C, CDCl3
101 MHz
f1 (ppm)

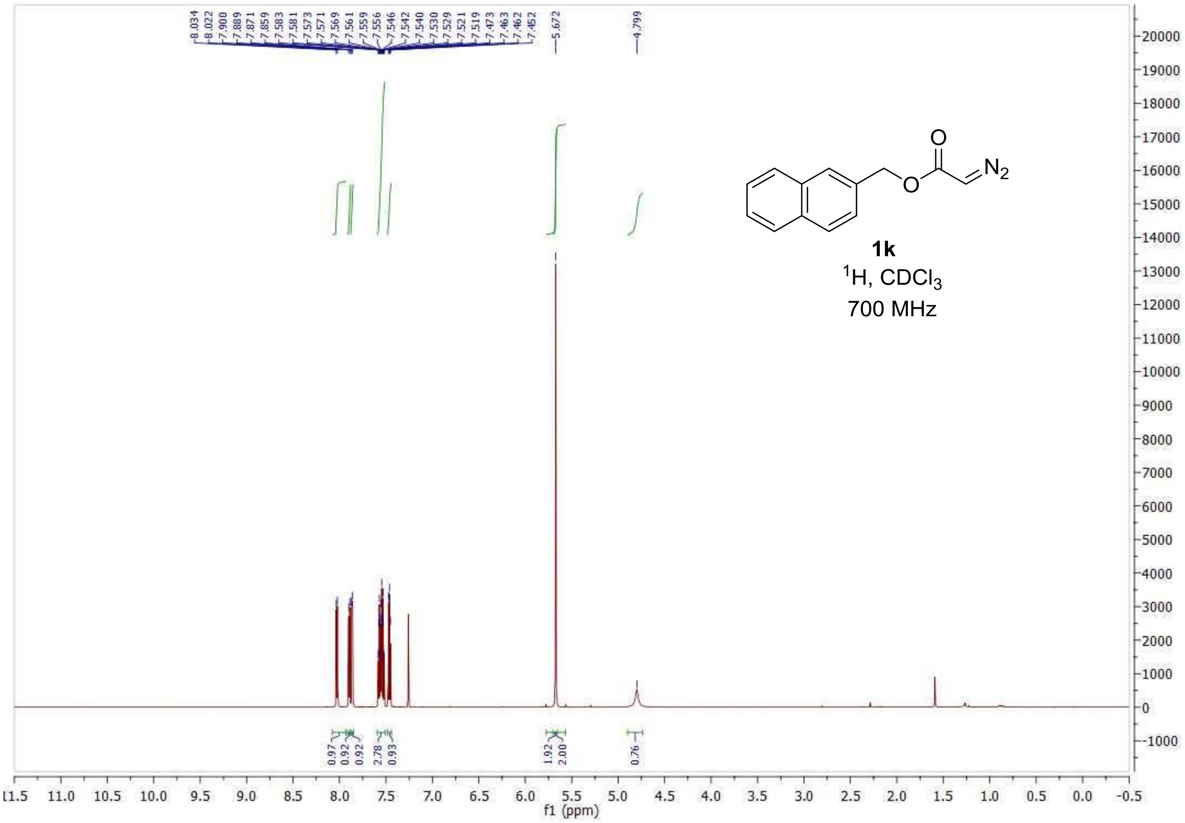
1k
1H, CDCl3
700 MHz
f1 (ppm)

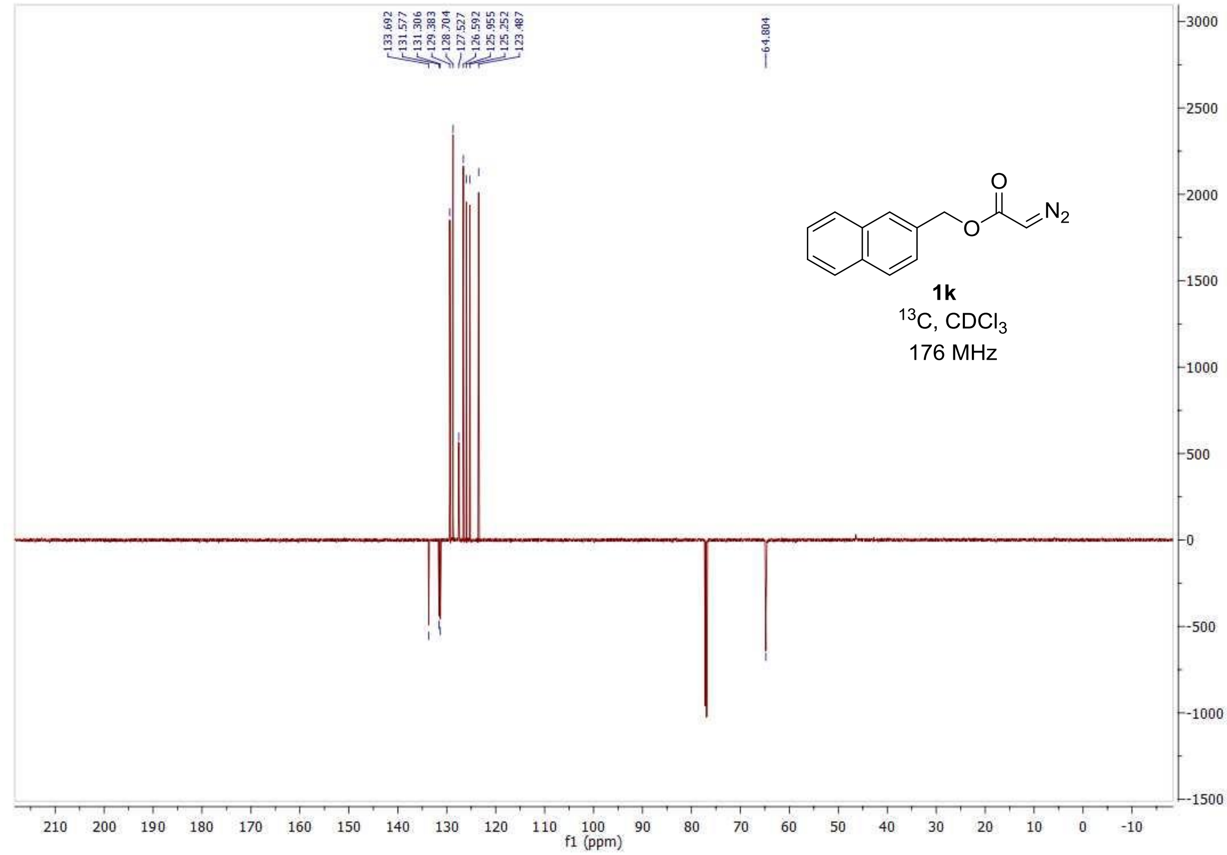
1k
13C, CDCl3
176 MHz
f1 (ppm)

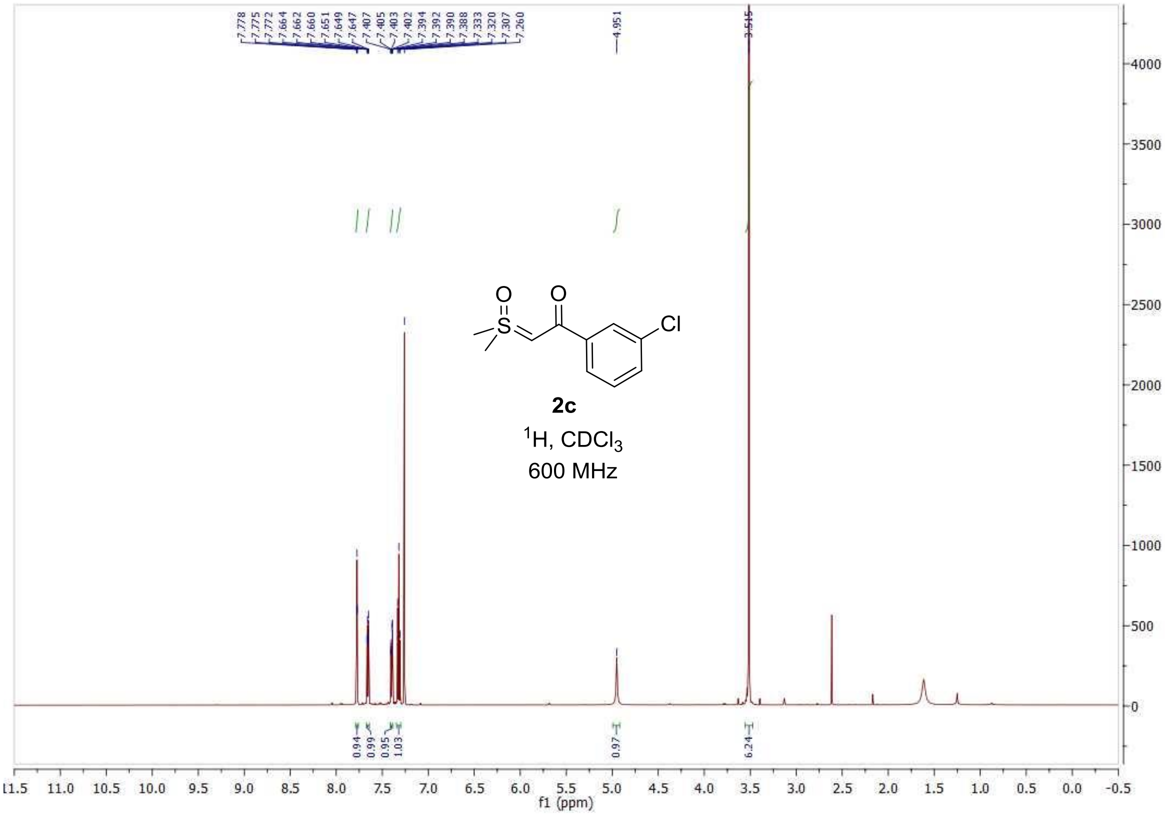
2c
^{1}H, $CDCl_3$
600 MHz
f1 (ppm)

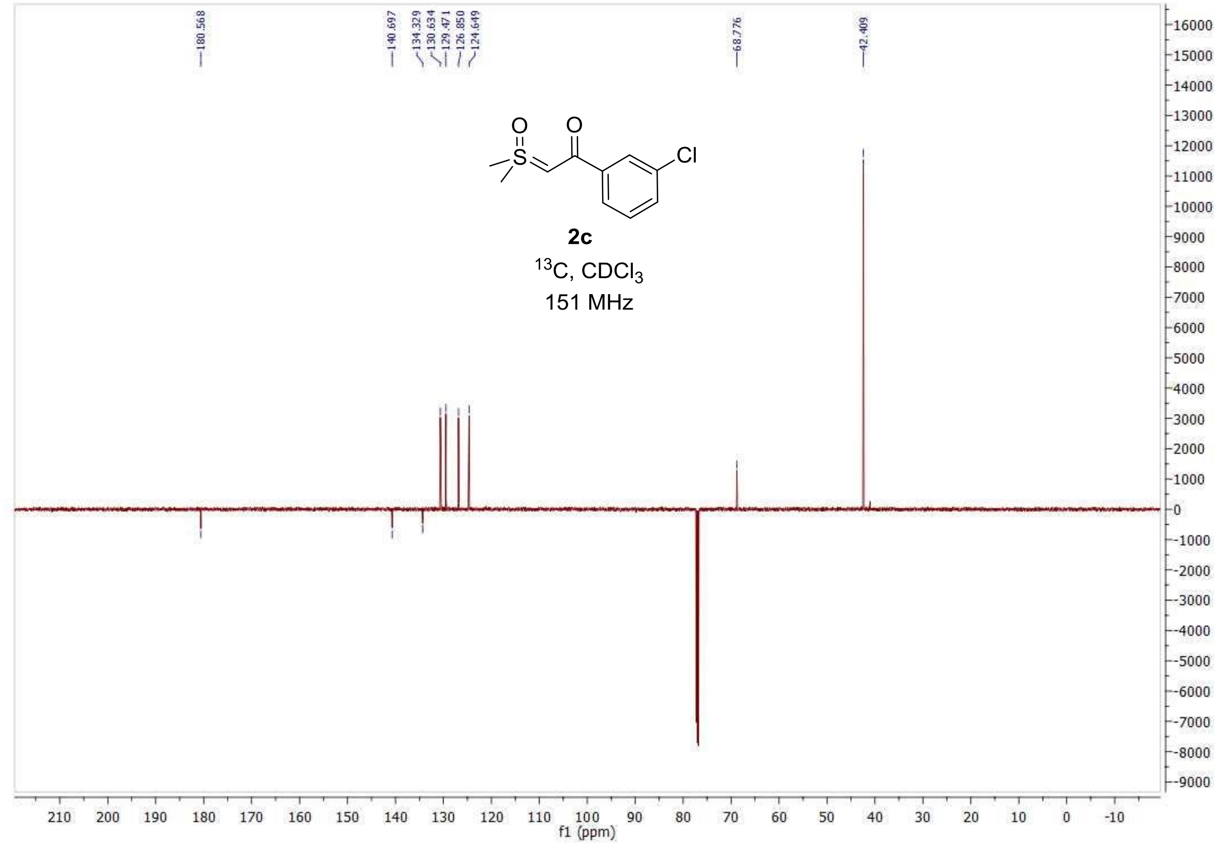
180.568
140.697
134.329
130.634
129.471
126.850
124.649
68.776
42.409
2c
^{13}C, $CDCl_3$
151 MHz
f1 (ppm)

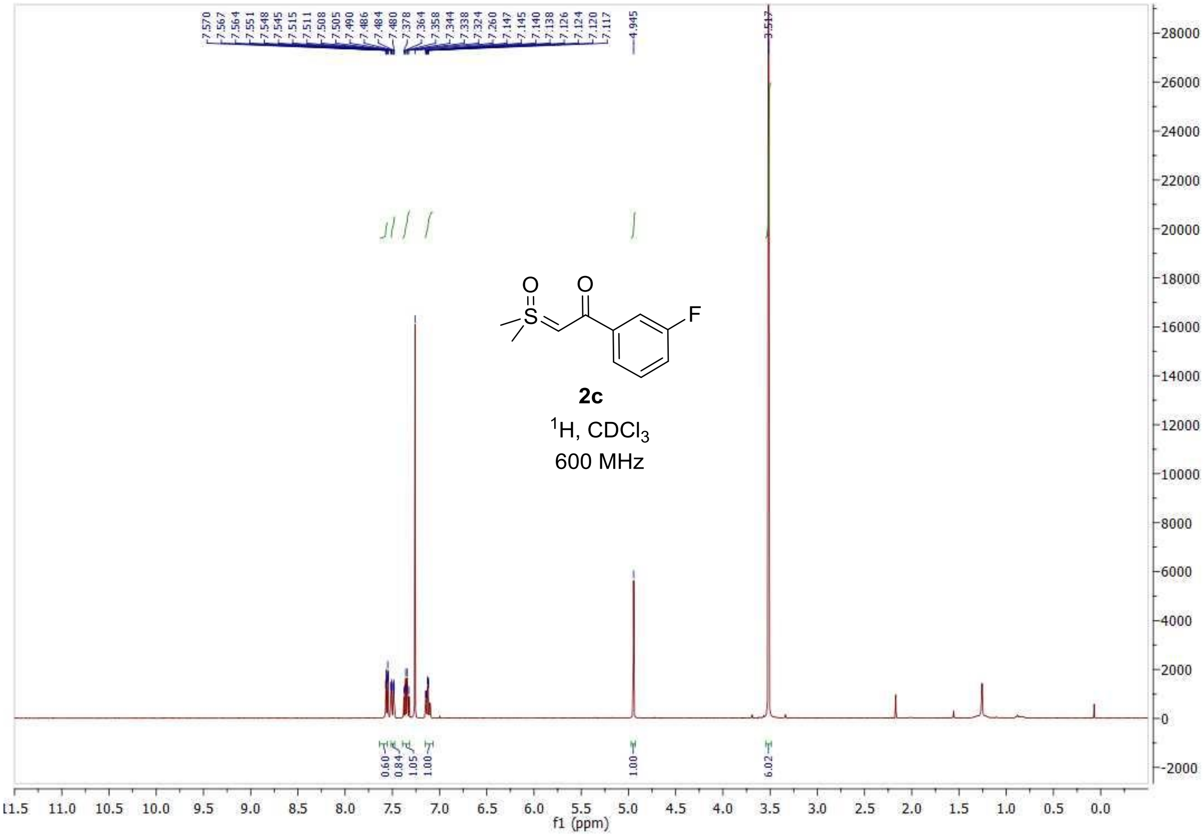
2c
^{1}H, CDCl$_3$
600 MHz
f1 (ppm)

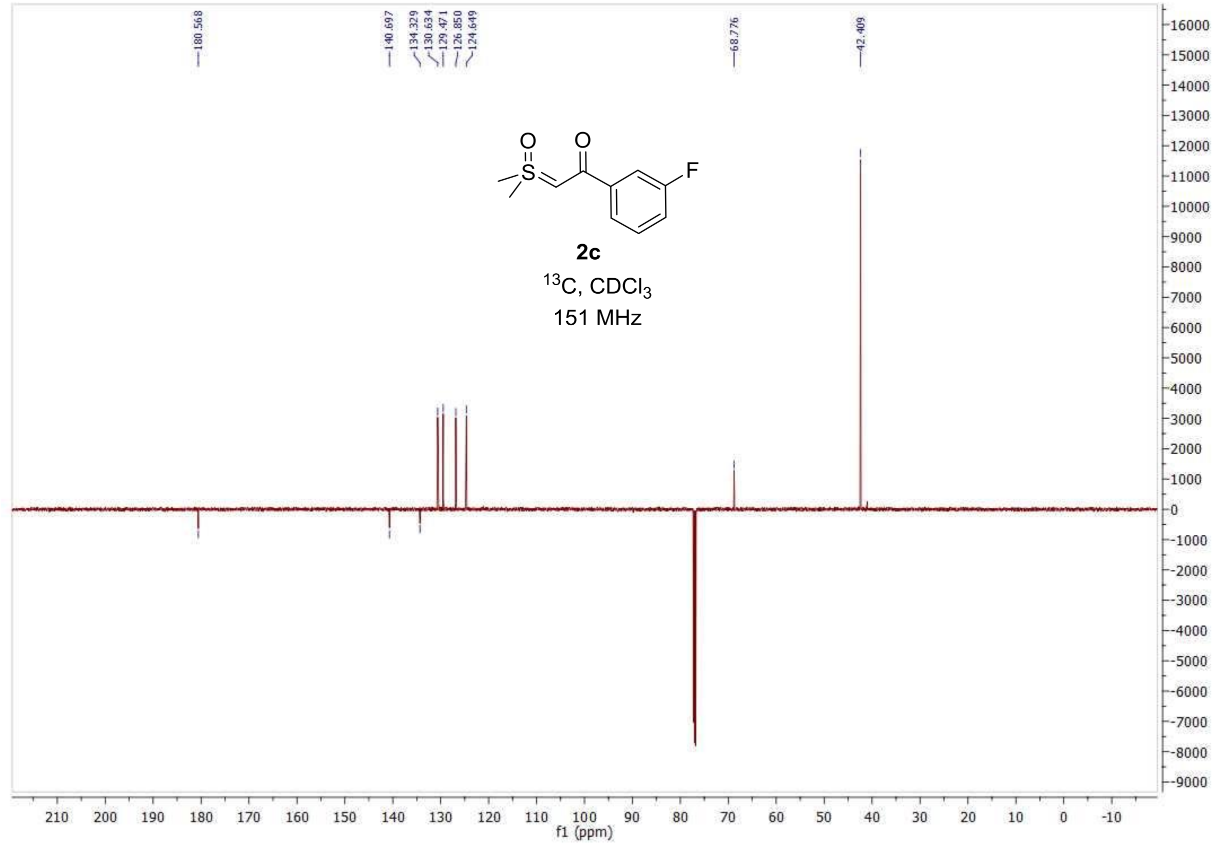
2c
^{13}C, CDCl$_3$
151 MHz
f1 (ppm)

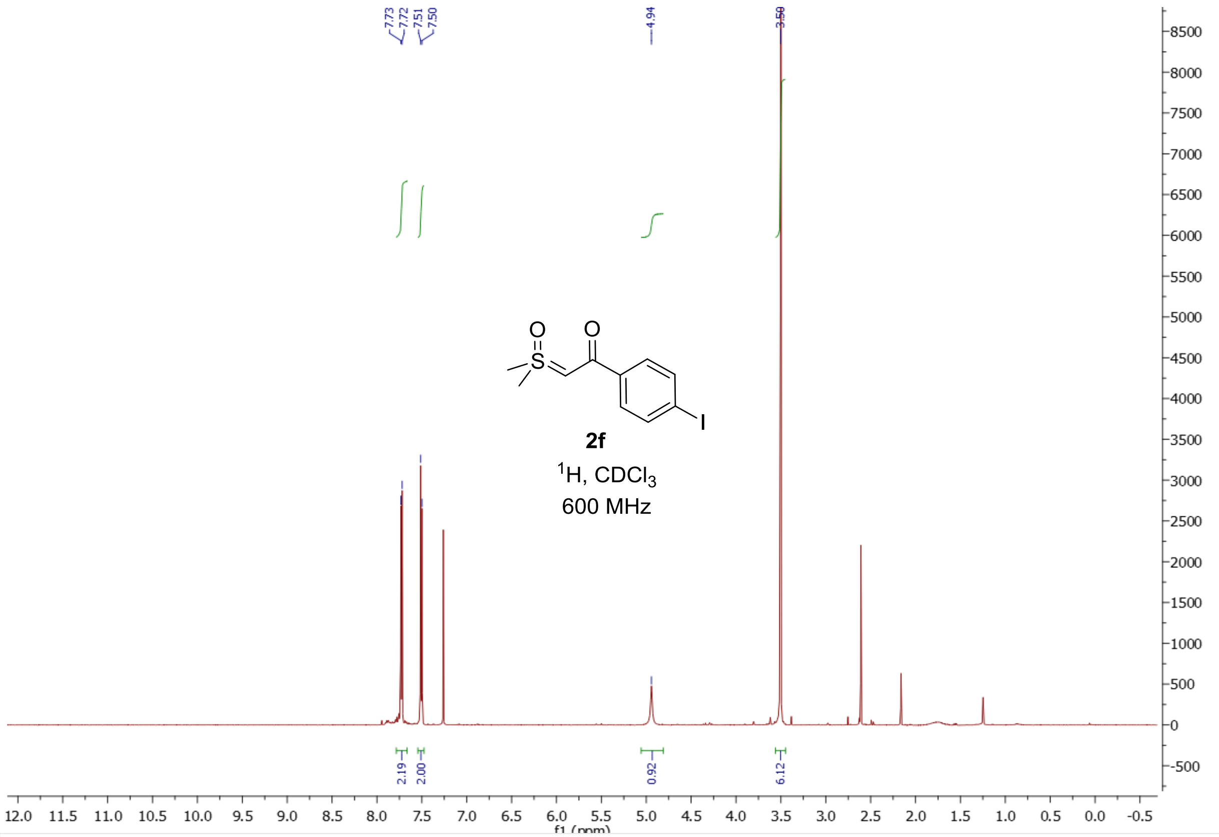
2f
^{1}H, CDCl$_3$
600 MHz

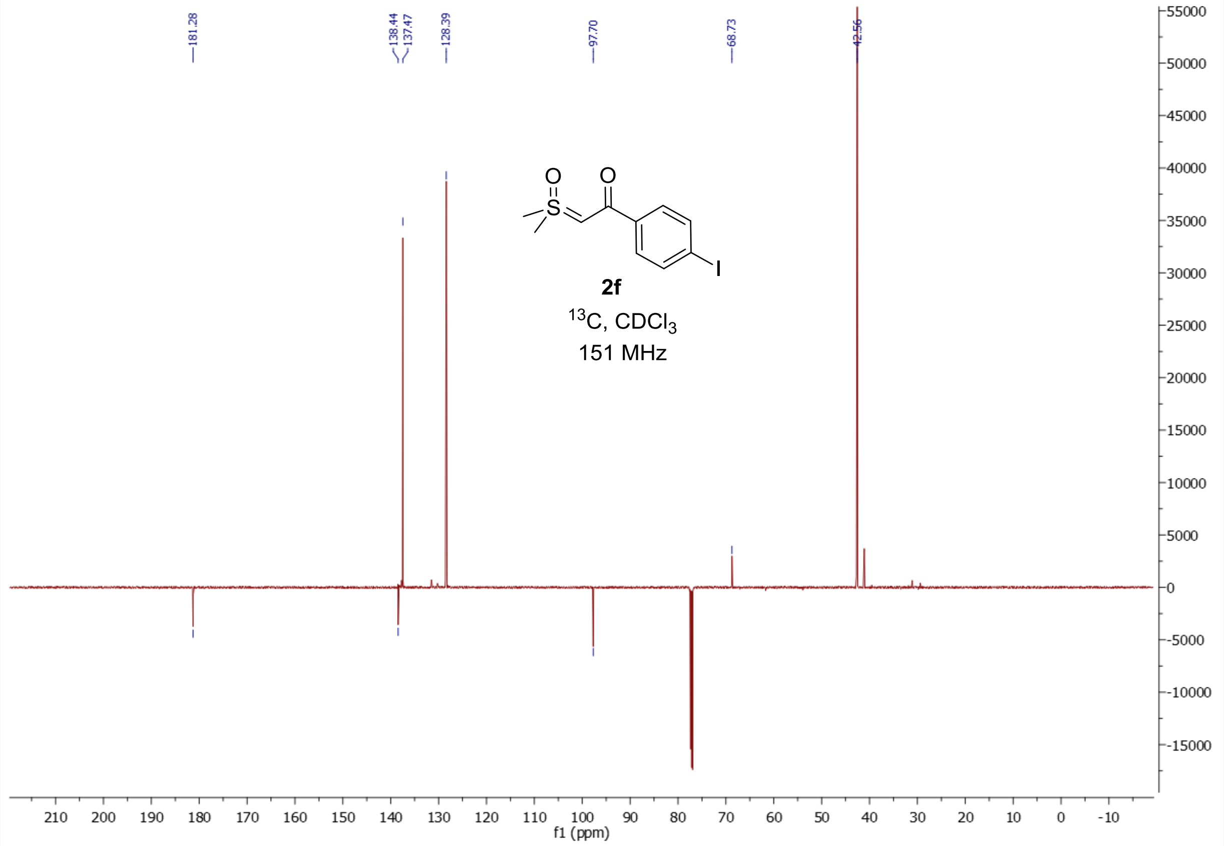
2f
^{13}C, CDCl$_3$
151 MHz

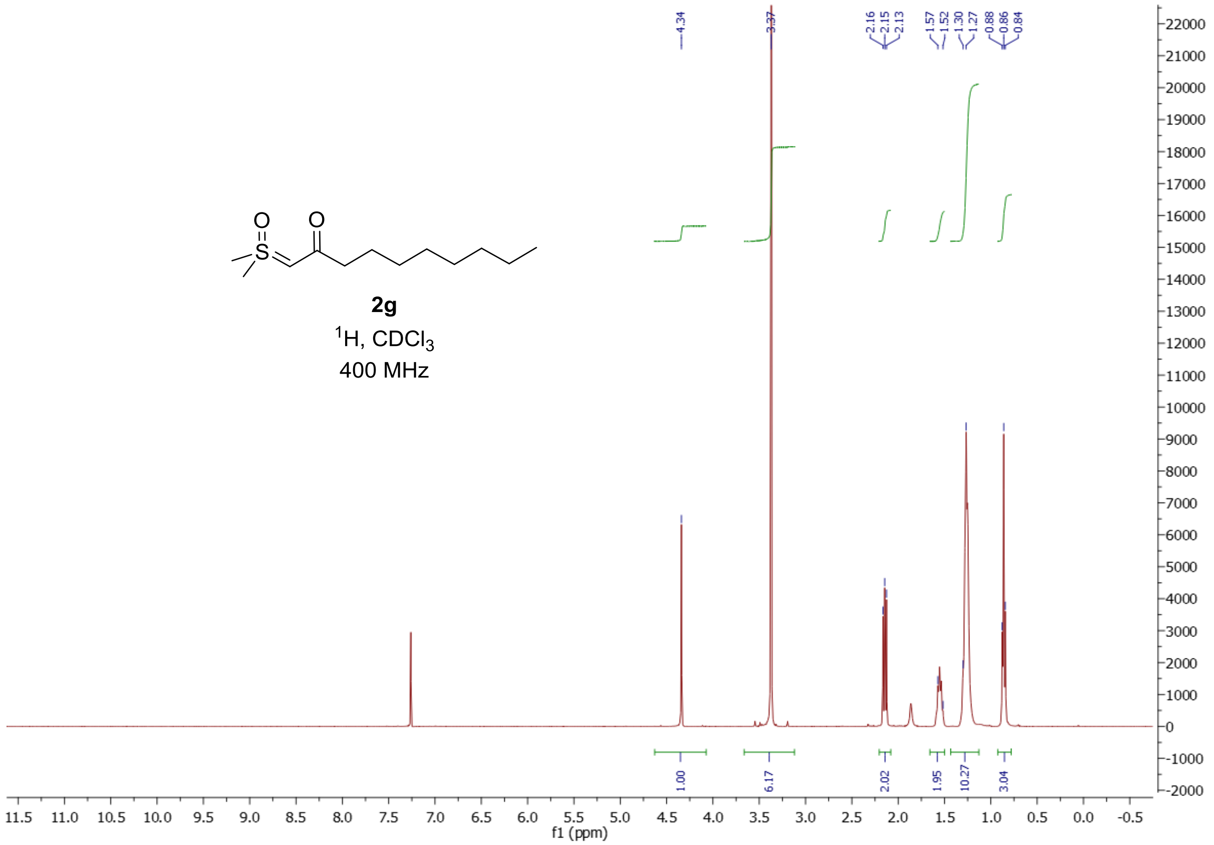
2g
^{1}H, CDCl$_3$
400 MHz
4.34
3.37
2.16
2.15
2.13
1.57
1.52
1.30
1.27
0.88
0.86
0.84
1.00
6.17
2.02
1.95
10.27
3.04
f1 (ppm)

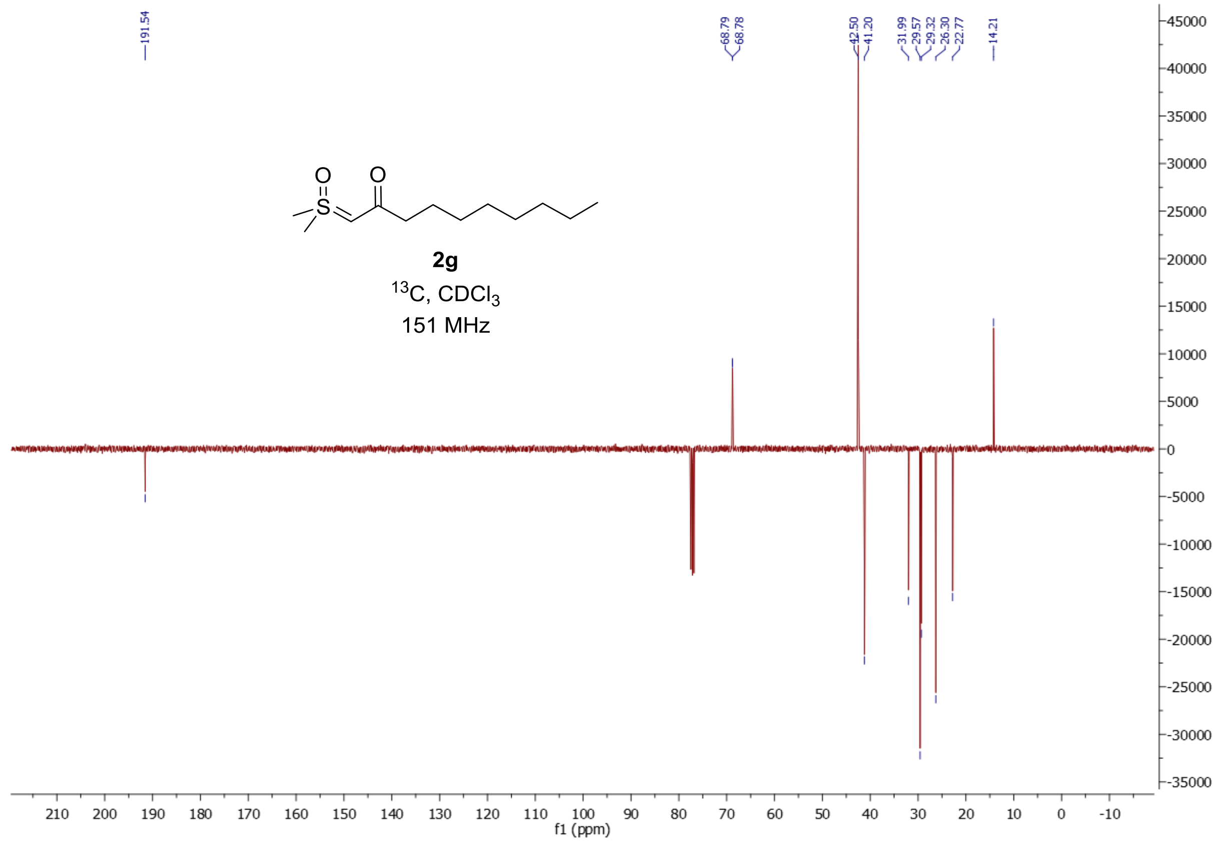
191.54
68.79
68.78
42.50
41.20
31.99
29.57
29.32
26.30
22.77
14.21
2g
^{13}C, CDCl$_3$
151 MHz
f1 (ppm)

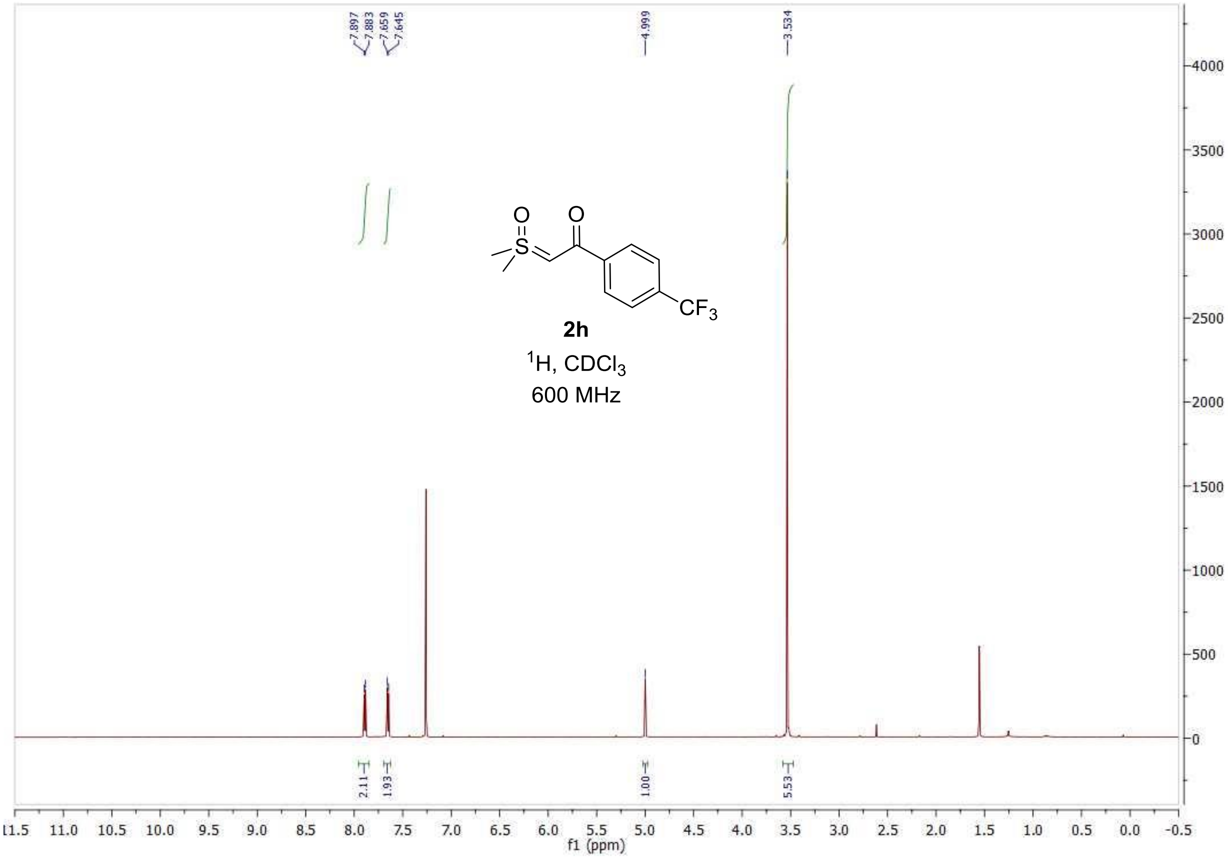
7.897
7.883
7.659
7.645
4.999
3.534
2h
^{1}H, CDCl$_3$
600 MHz
2.11
1.93
1.00
5.53
f1 (ppm)

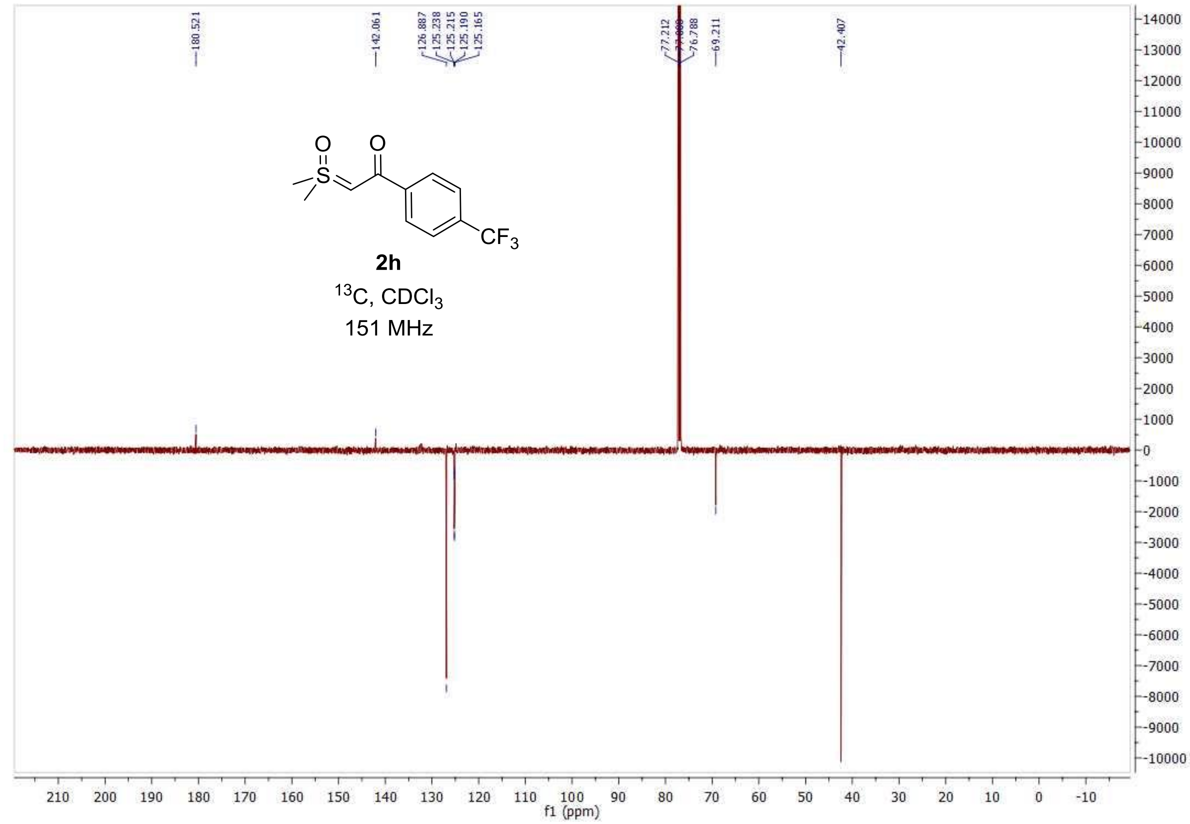
180.521
142.061
126.887
125.238
125.215
125.190
125.165
77.212
76.788
69.211
42.407
2h
^{13}C, CDCl$_3$
151 MHz
f1 (ppm)

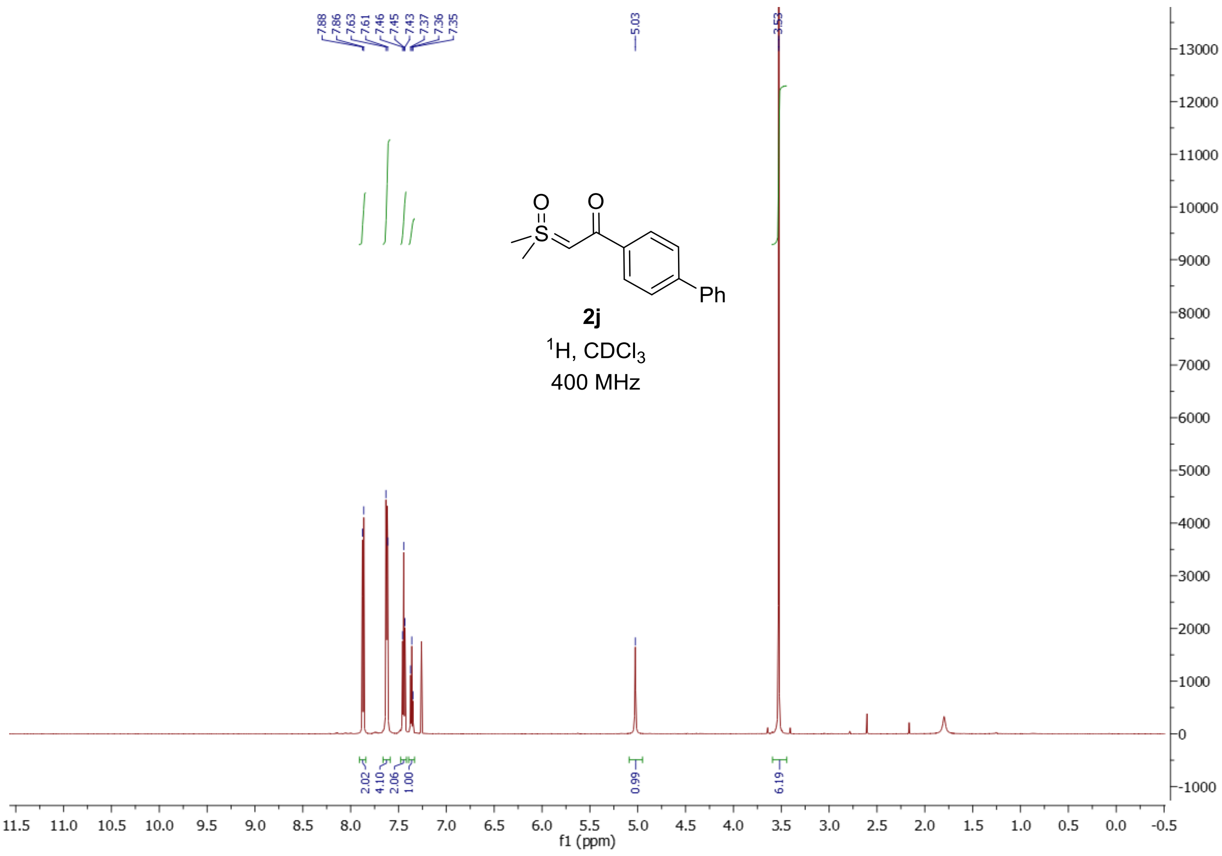

7.88
7.86
7.63
7.61
7.46
7.45
7.43
7.37
7.36
7.35
5.03
3.53
2j
^{1}H, CDCl$_3$
400 MHz
Ph
2.02
4.10
2.06
1.00
0.99
6.19
f1 (ppm)

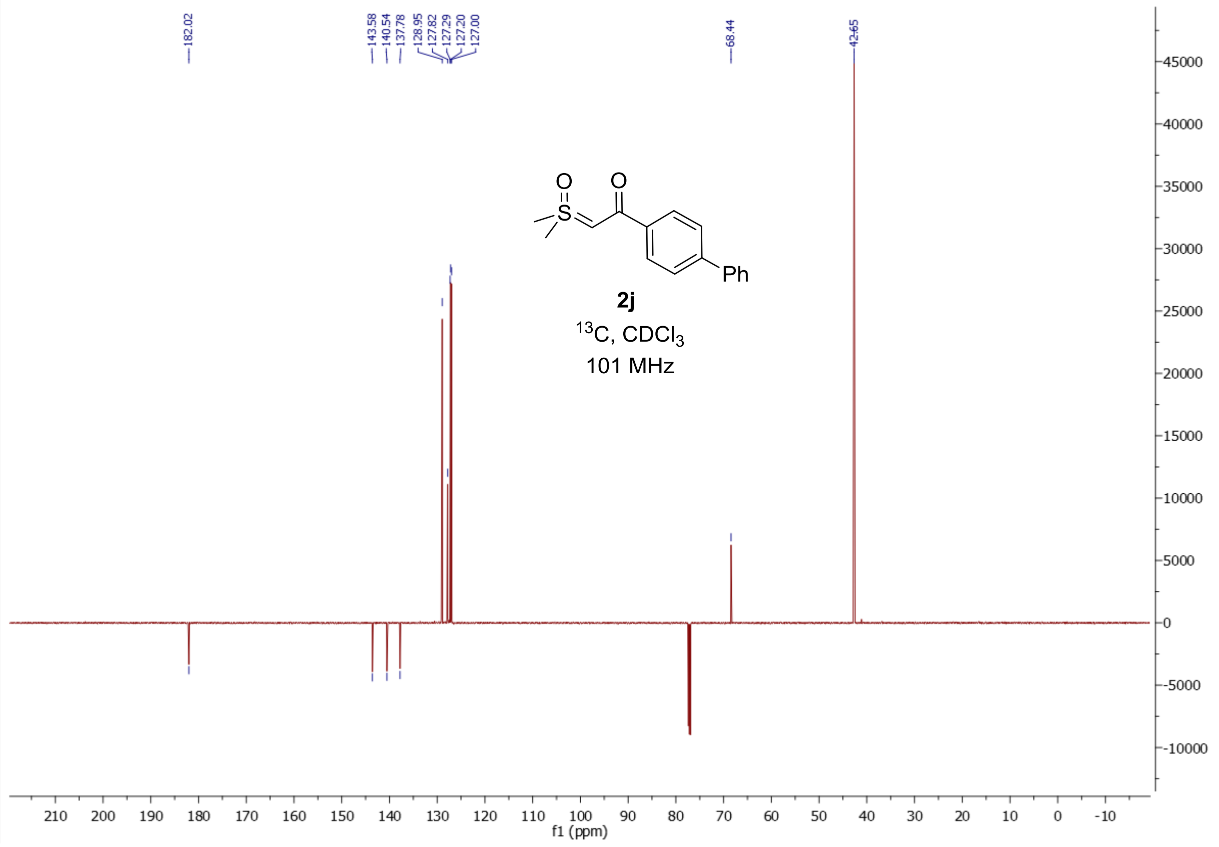

182.02
143.58
140.54
137.78
128.95
127.82
127.29
127.20
127.00
68.44
42.65
2j
^{13}C, CDCl$_3$
101 MHz
Ph
f1 (ppm)

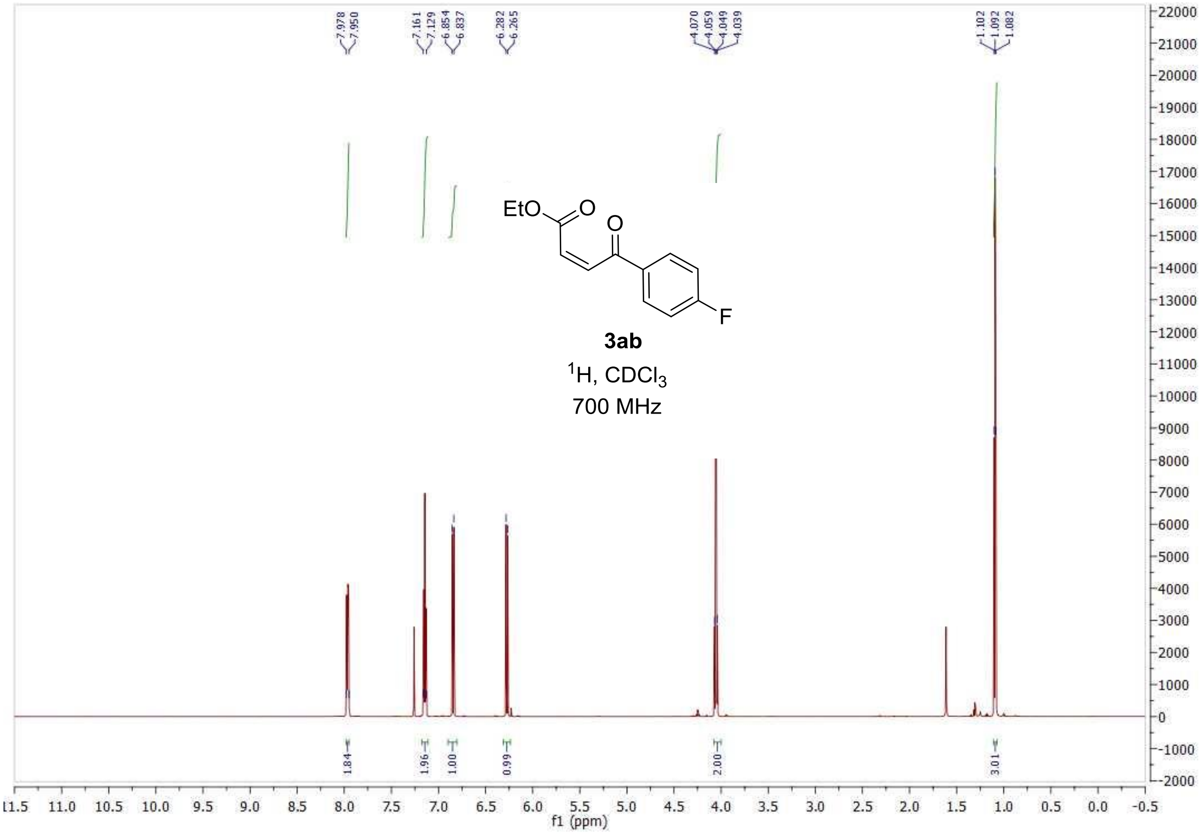
EtO
O
O
F
3ab
1H, CDCl3
700 MHz
f1 (ppm)

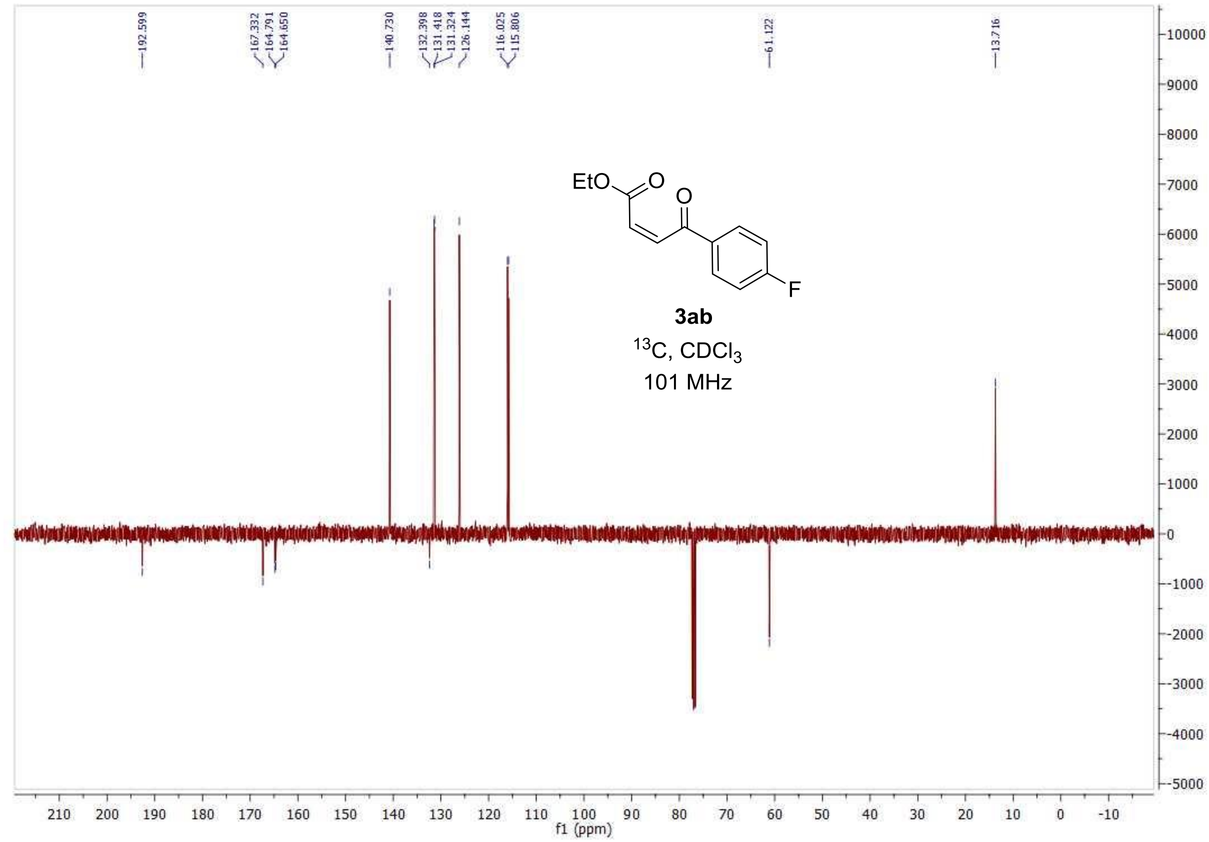
EtO
O
O
F
3ab
13C, CDCl3
101 MHz
f1 (ppm)

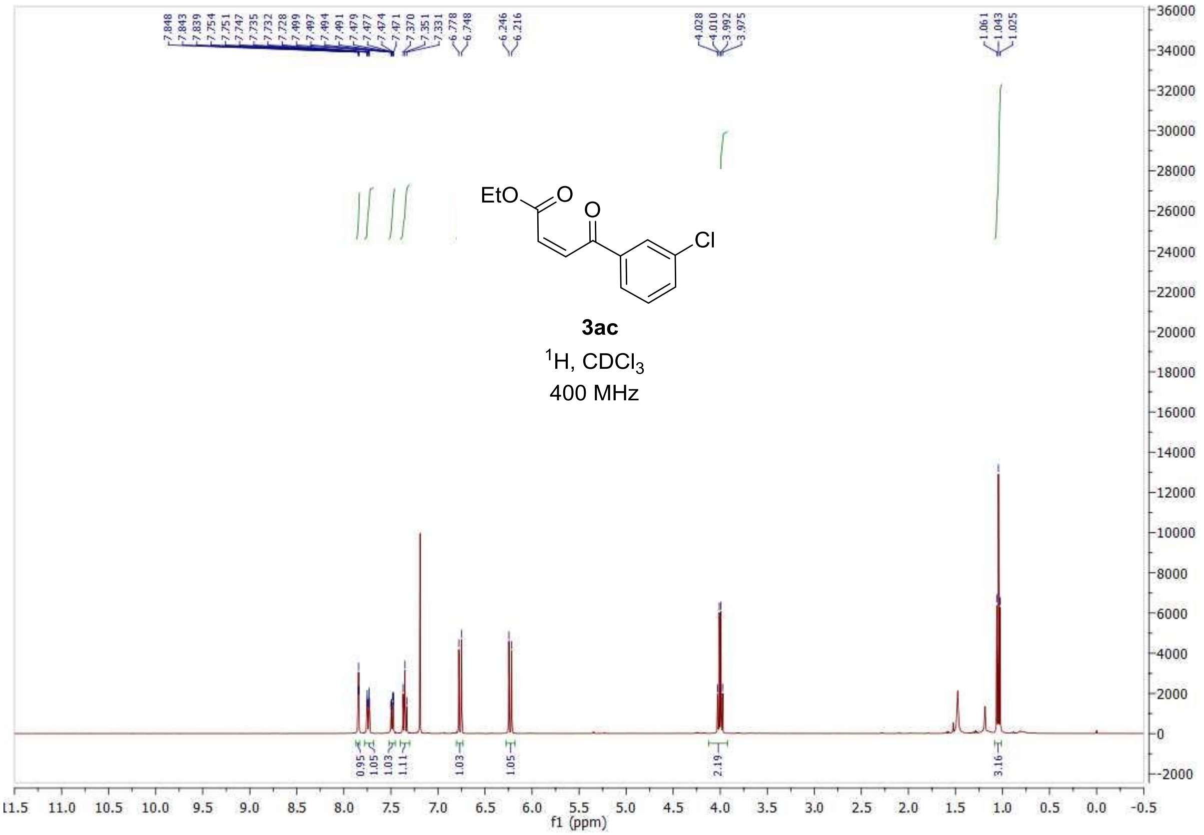

3ac
^{1}H, CDCl$_3$
400 MHz
EtO
O
O
Cl
f1 (ppm)

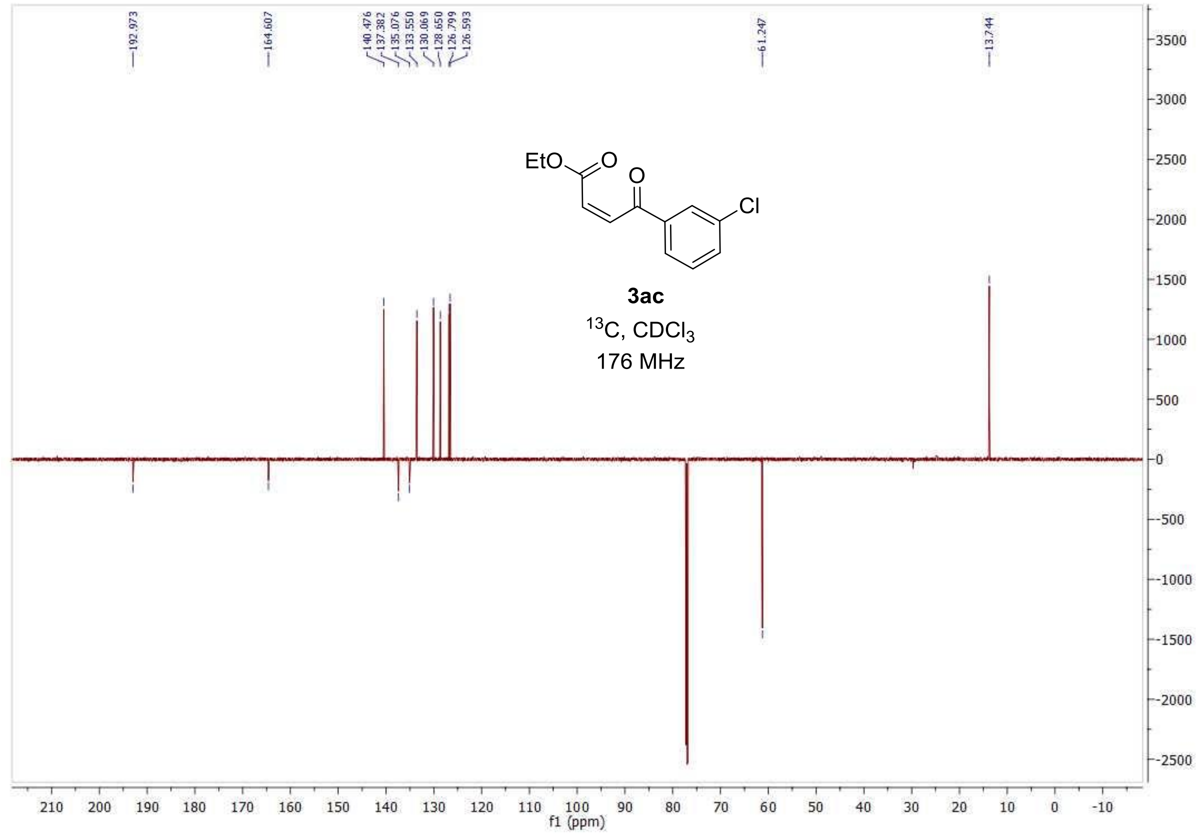

3ac
^{13}C, CDCl$_3$
176 MHz
EtO
O
O
Cl
f1 (ppm)

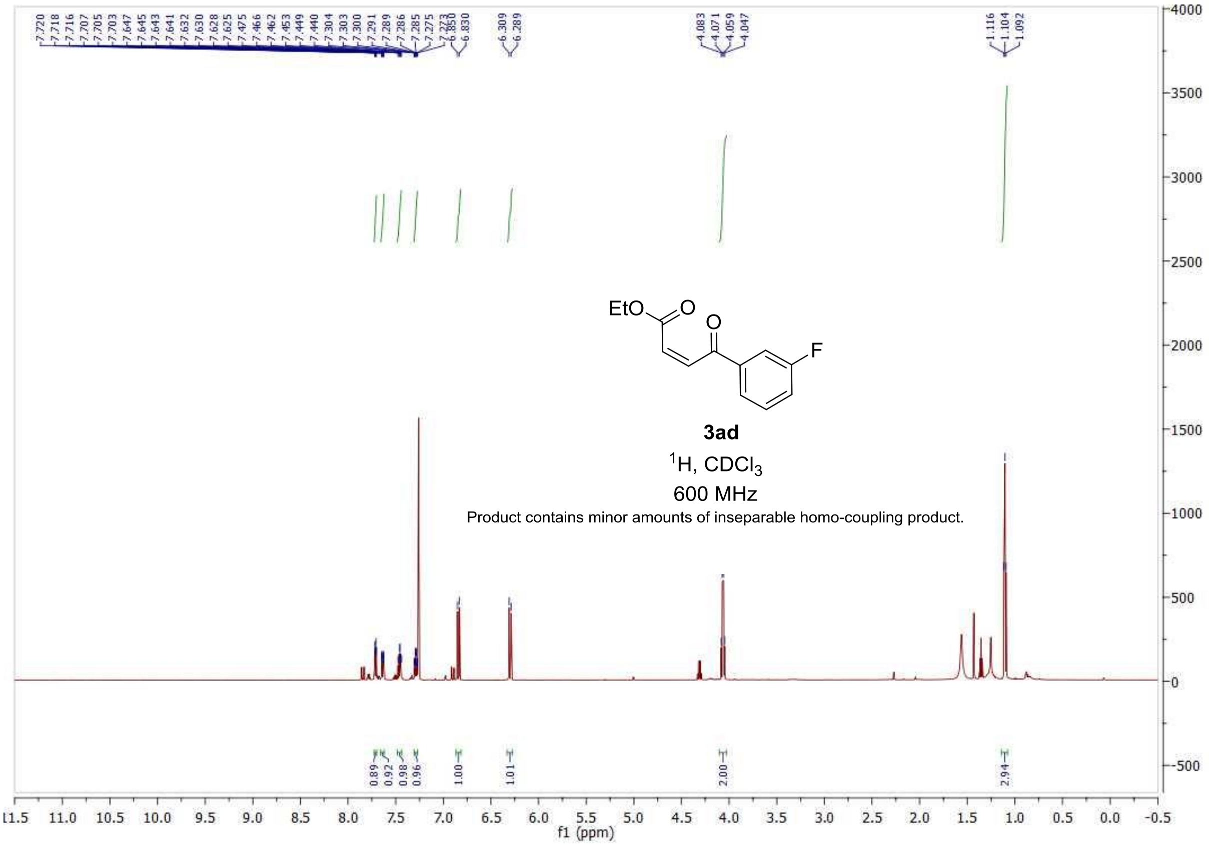
EtO
O
O
F
3ad
1H, CDCl3
600 MHz
Product contains minor amounts of inseparable homo-coupling product.
f1 (ppm)

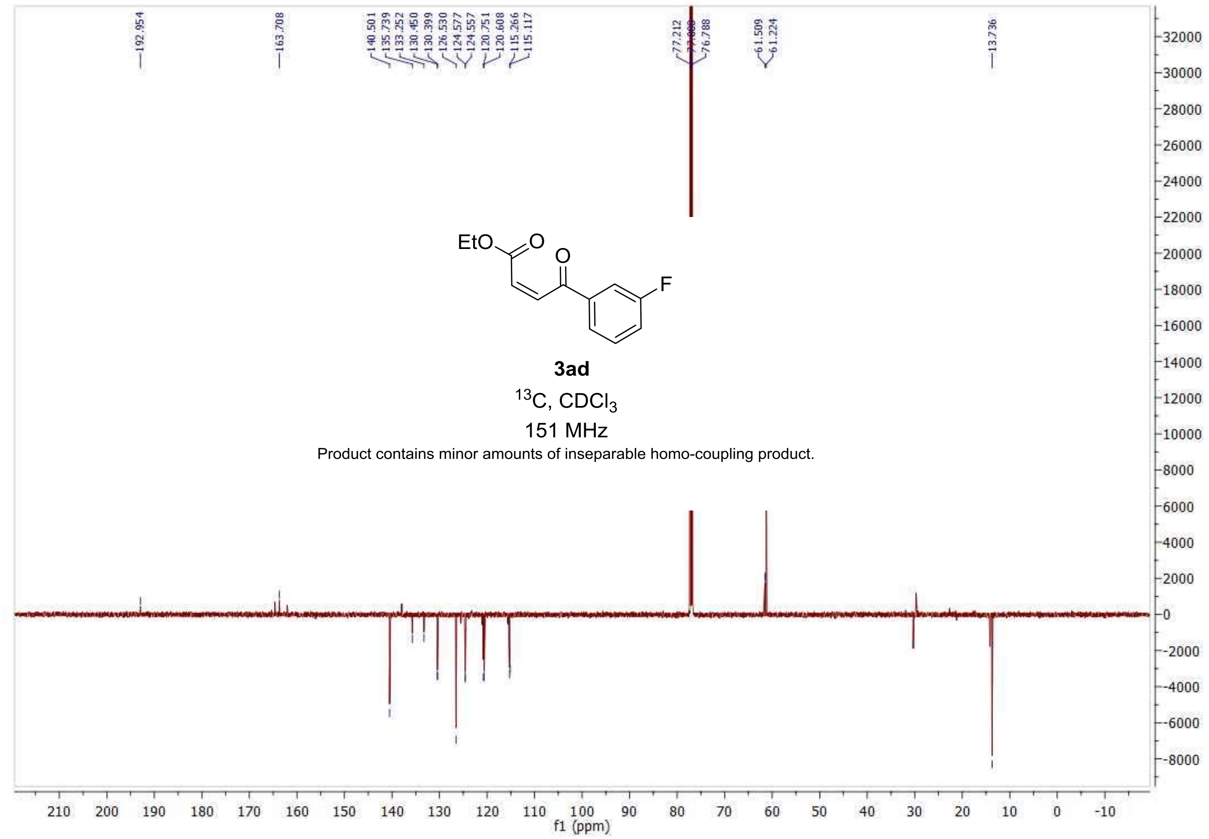
EtO
O
O
F
3ad
13C, CDCl3
151 MHz
Product contains minor amounts of inseparable homo-coupling product.
f1 (ppm)

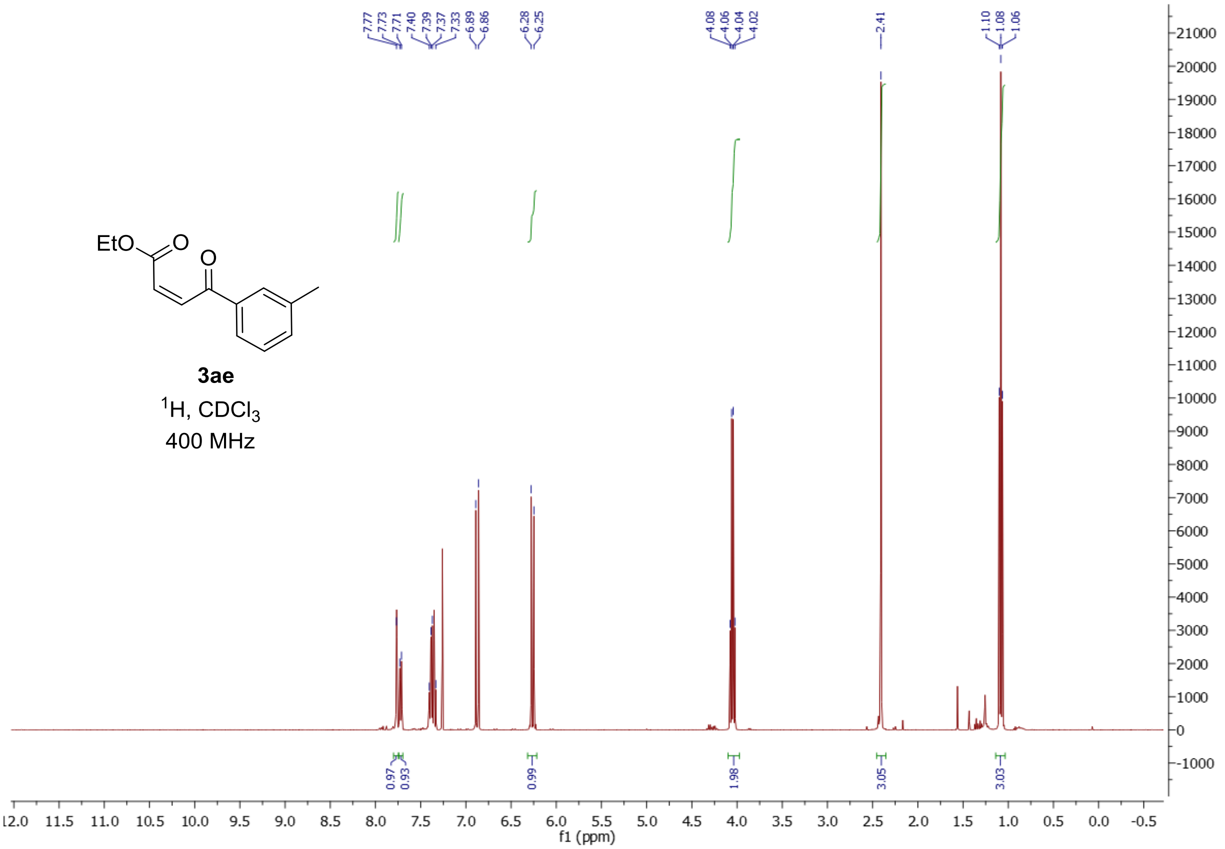
7.77 7.73 7.71 7.40 7.39 7.37 7.33 6.89 6.86 6.28 6.25 4.08 4.06 4.04 4.02 2.41 1.10 1.08 1.06
EtO
O
O
3ae
1H, CDCl3
400 MHz
0.97 0.93 0.99 1.98 3.05 3.03
f1 (ppm)

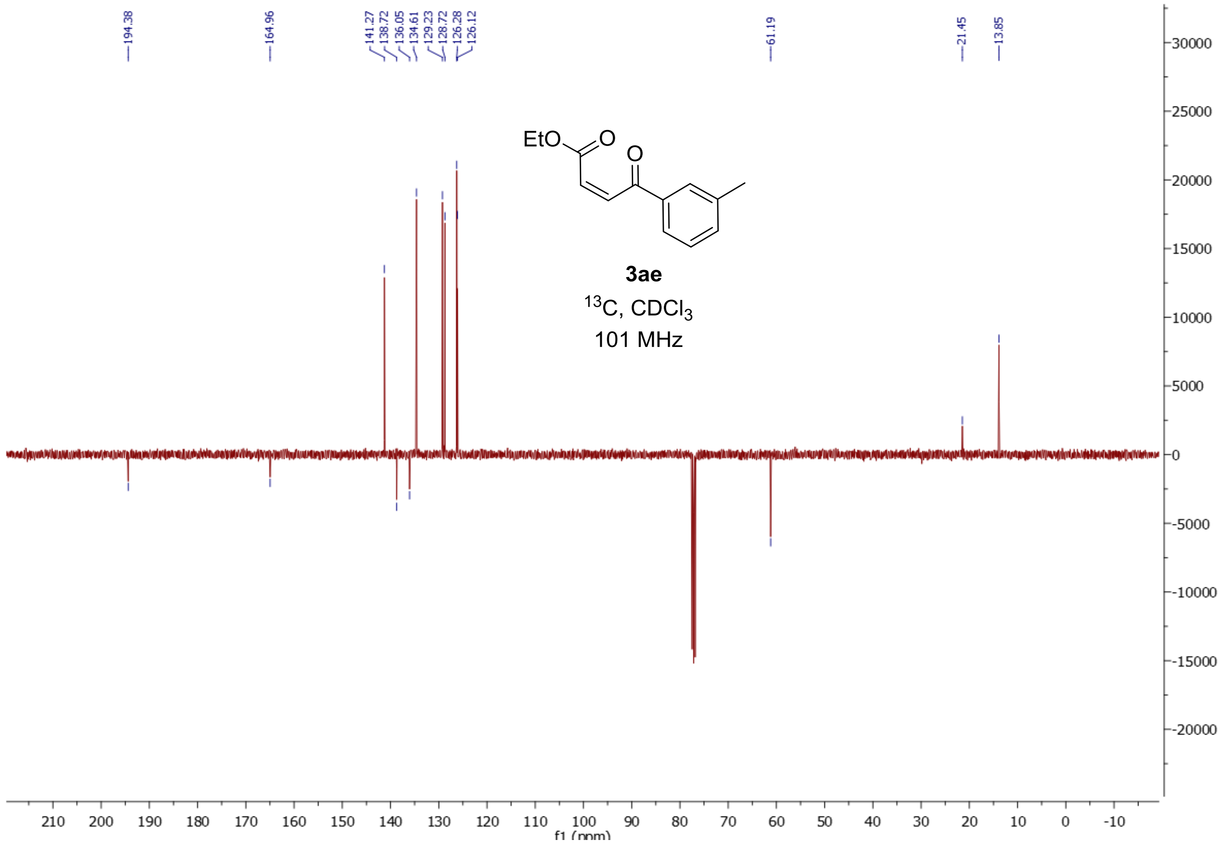
194.38 164.96 141.27 138.72 136.05 134.61 129.23 128.72 126.28 126.12 61.19 21.45 13.85
EtO
O
O
3ae
13C, CDCl3
101 MHz
f1 (ppm)

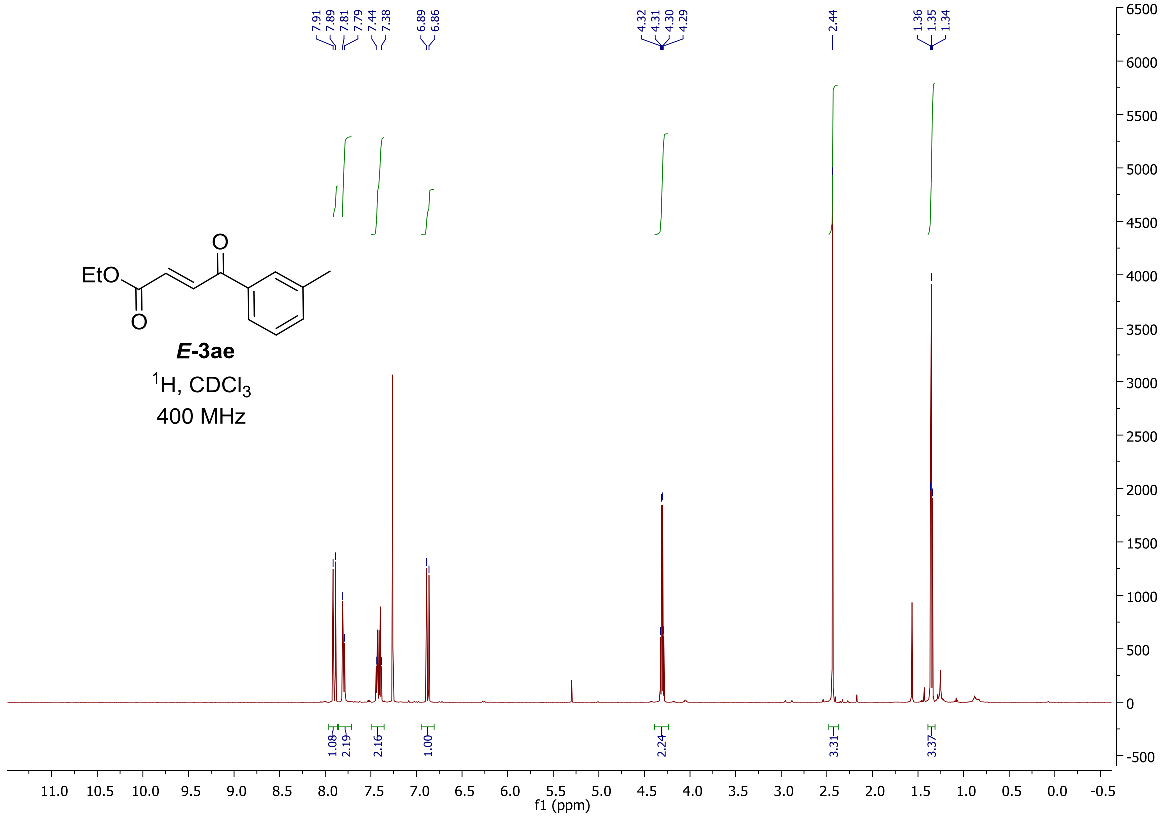
EtO
O
O
E-3ae
^{1}H, CDCl$_3$
400 MHz
7.91
7.89
7.81
7.79
7.44
7.38
6.89
6.86
4.32
4.31
4.30
4.29
2.44
1.36
1.35
1.34
1.08
2.19
2.16
1.00
2.24
3.31
3.37
f1 (ppm)

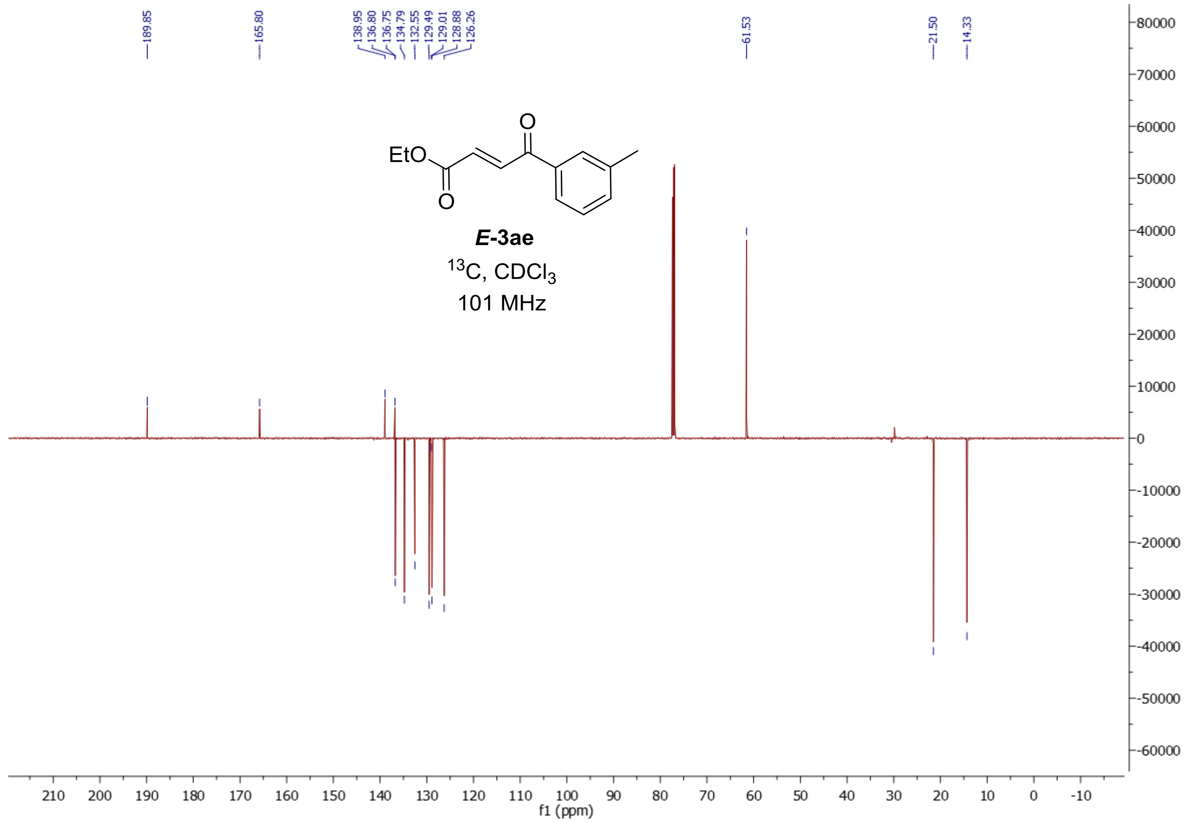
189.85
165.80
138.95
136.80
136.75
134.79
132.55
129.49
129.01
128.88
126.26
61.53
21.50
14.33
EtO
O
O
E-3ae
^{13}C, CDCl$_3$
101 MHz
f1 (ppm)

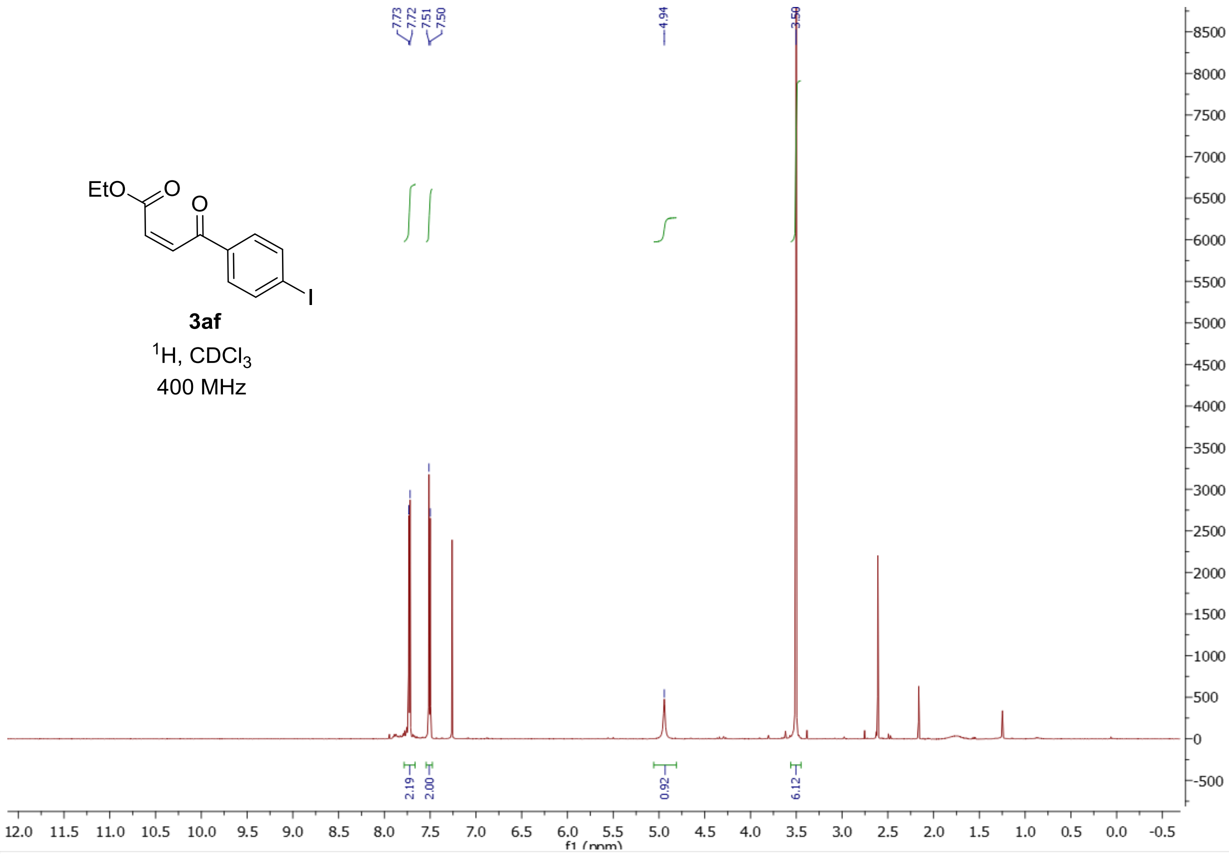
7.73
7.72
7.51
7.50
4.94
3.50
3af
^{1}H, $CDCl_3$
400 MHz
2.19
2.00
0.92
6.12
f1 (ppm)

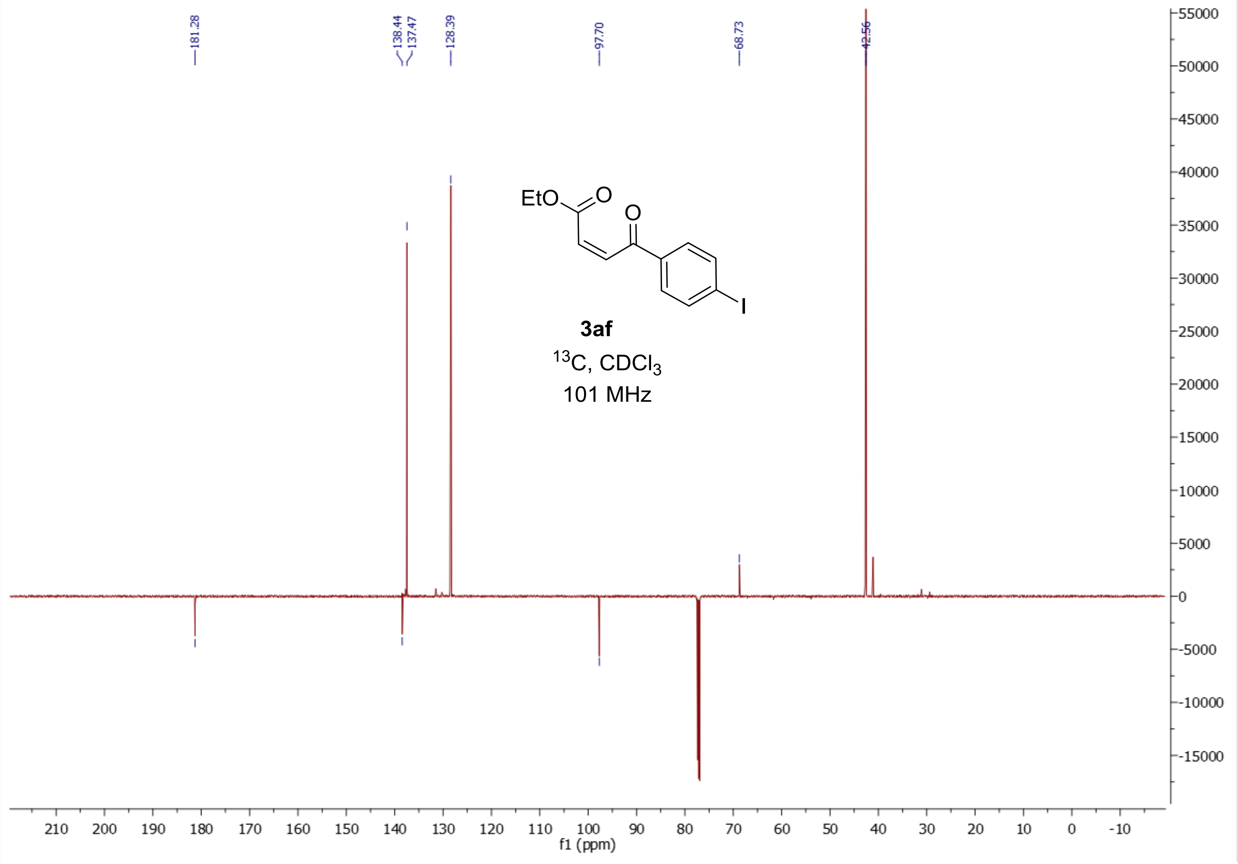
181.28
138.44
137.47
128.39
97.70
68.73
42.56
3af
^{13}C, $CDCl_3$
101 MHz
f1 (ppm)

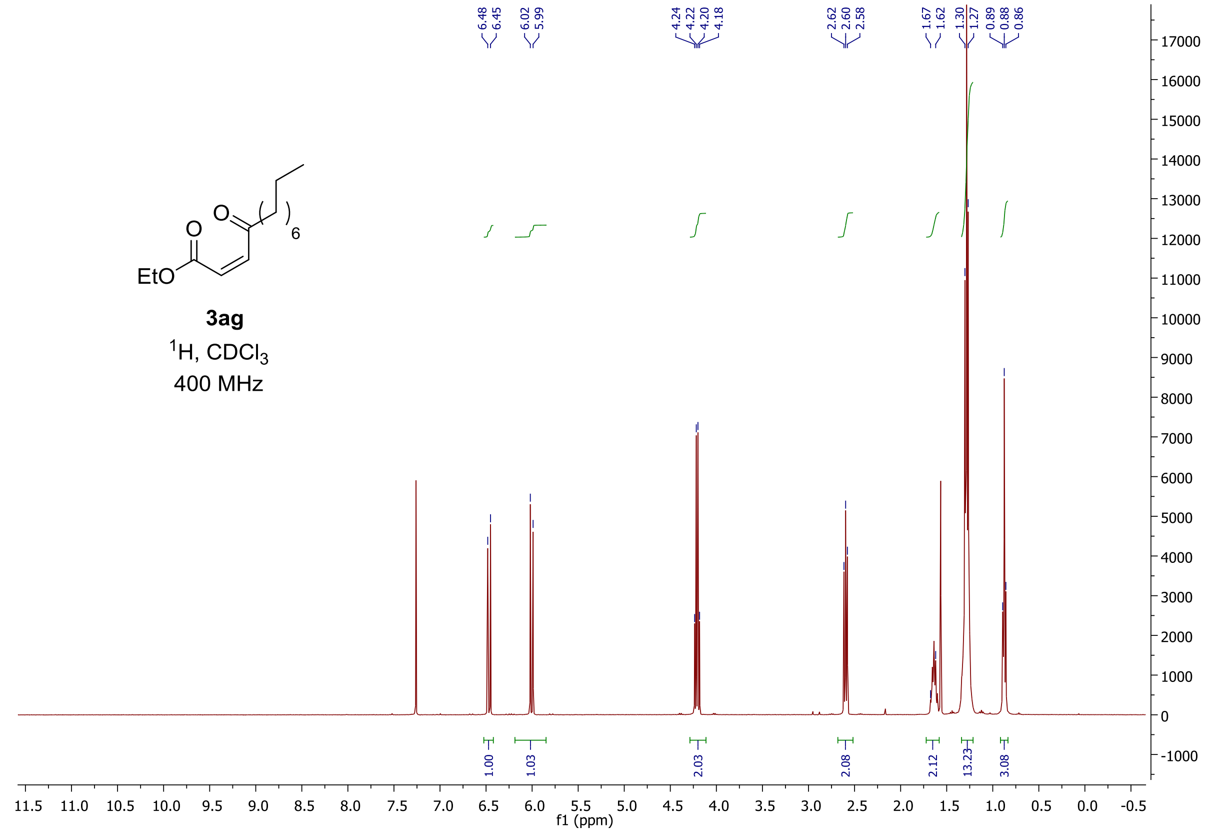
3ag
^{1}H, CDCl$_3$
400 MHz
6.48 6.45 6.02 5.99 4.24 4.22 4.20 4.18 2.62 2.60 2.58 1.67 1.62 1.30 1.27 0.89 0.88 0.86
1.00 1.03 2.03 2.08 2.12 13.23 3.08
f1 (ppm)

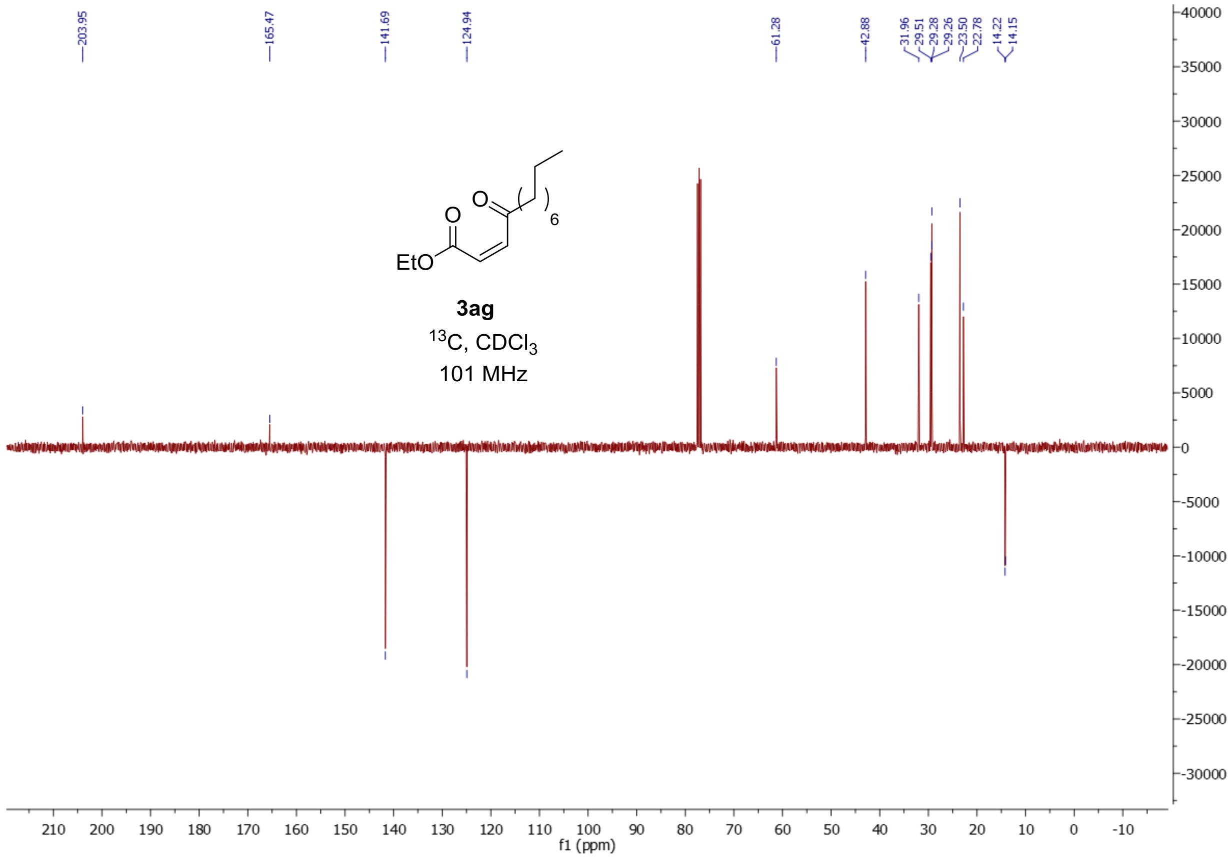
3ag
^{13}C, CDCl$_3$
101 MHz
203.95 165.47 141.69 124.94 61.28 42.88 31.96 29.51 29.28 29.26 23.50 22.78 14.22 14.15
f1 (ppm)

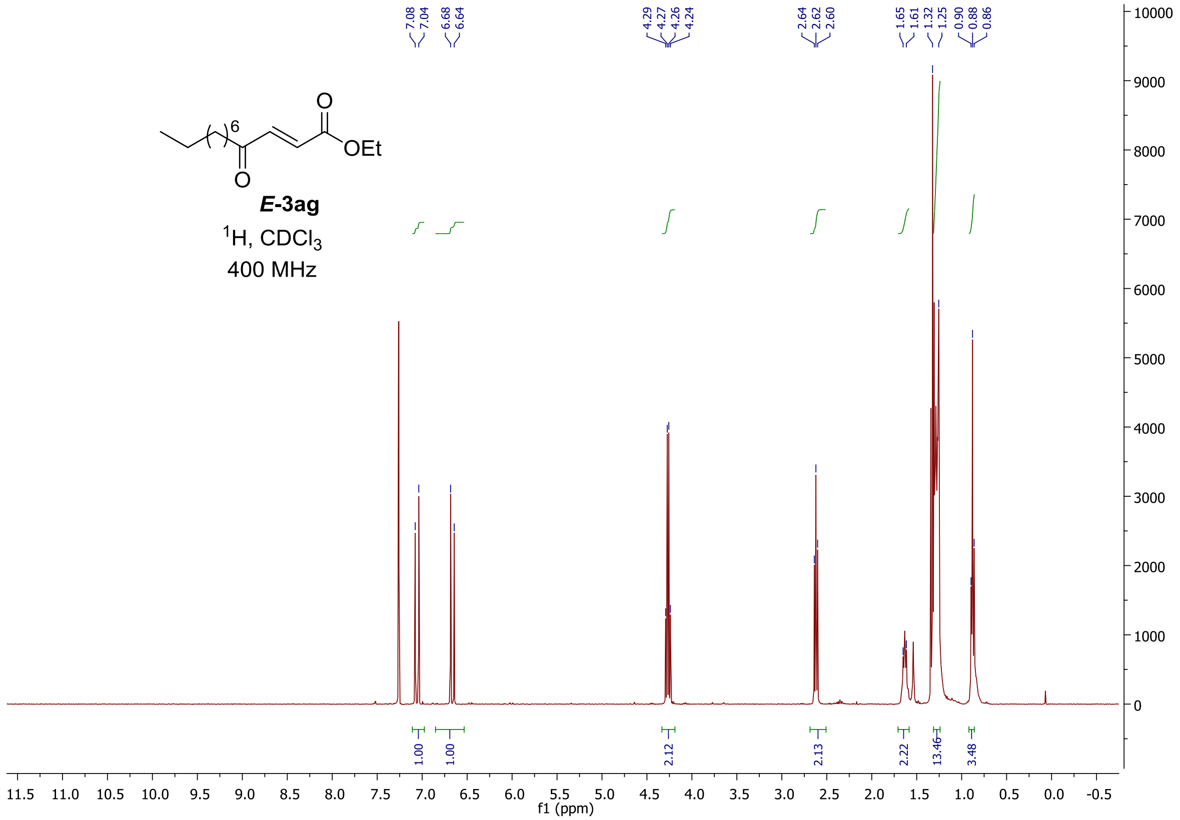

E-3ag
^{1}H, CDCl$_3$
400 MHz
7.08
7.04
6.68
6.64
4.29
4.27
4.26
4.24
2.64
2.62
2.60
1.65
1.61
1.32
1.25
0.90
0.88
0.86
1.00
1.00
2.12
2.13
2.22
13.46
3.48
f1 (ppm)

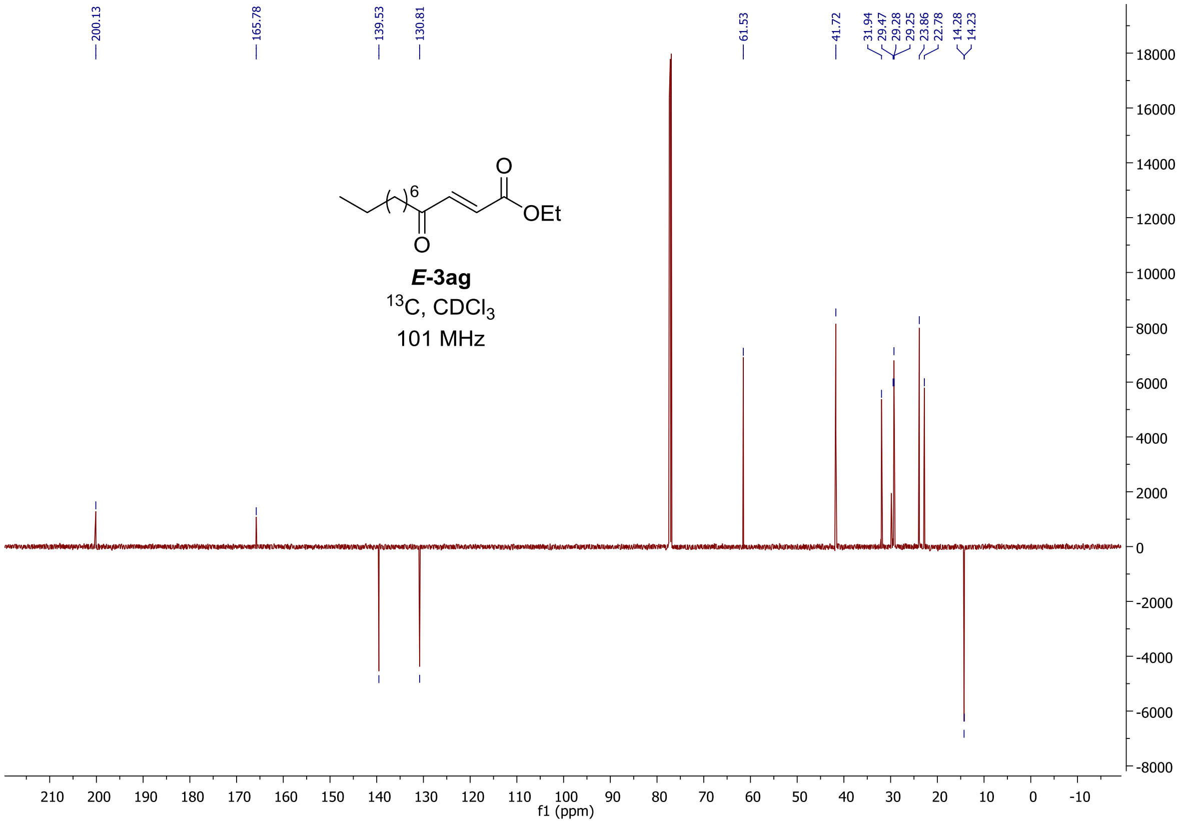

E-3ag
^{13}C, CDCl$_3$
101 MHz
200.13
165.78
139.53
130.81
61.53
41.72
31.94
29.47
29.28
29.25
23.86
22.78
14.28
14.23
f1 (ppm)

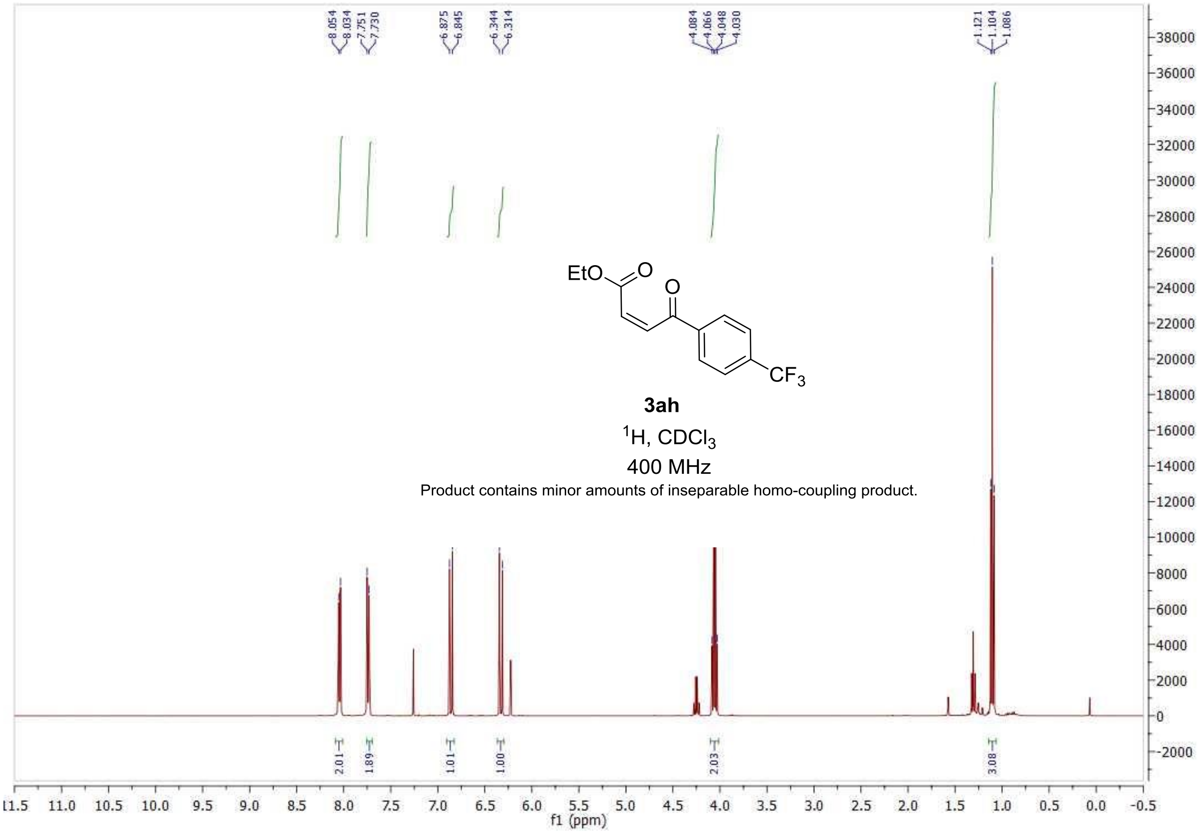
8.054
8.034
7.751
7.730
6.875
6.845
6.344
6.314
4.084
4.066
4.048
4.030
1.121
1.104
1.086
EtO
O
O
CF3
3ah
1H, CDCl3
400 MHz
Product contains minor amounts of inseparable homo-coupling product.
2.01
1.89
1.01
1.00
2.03
3.08
f1 (ppm)

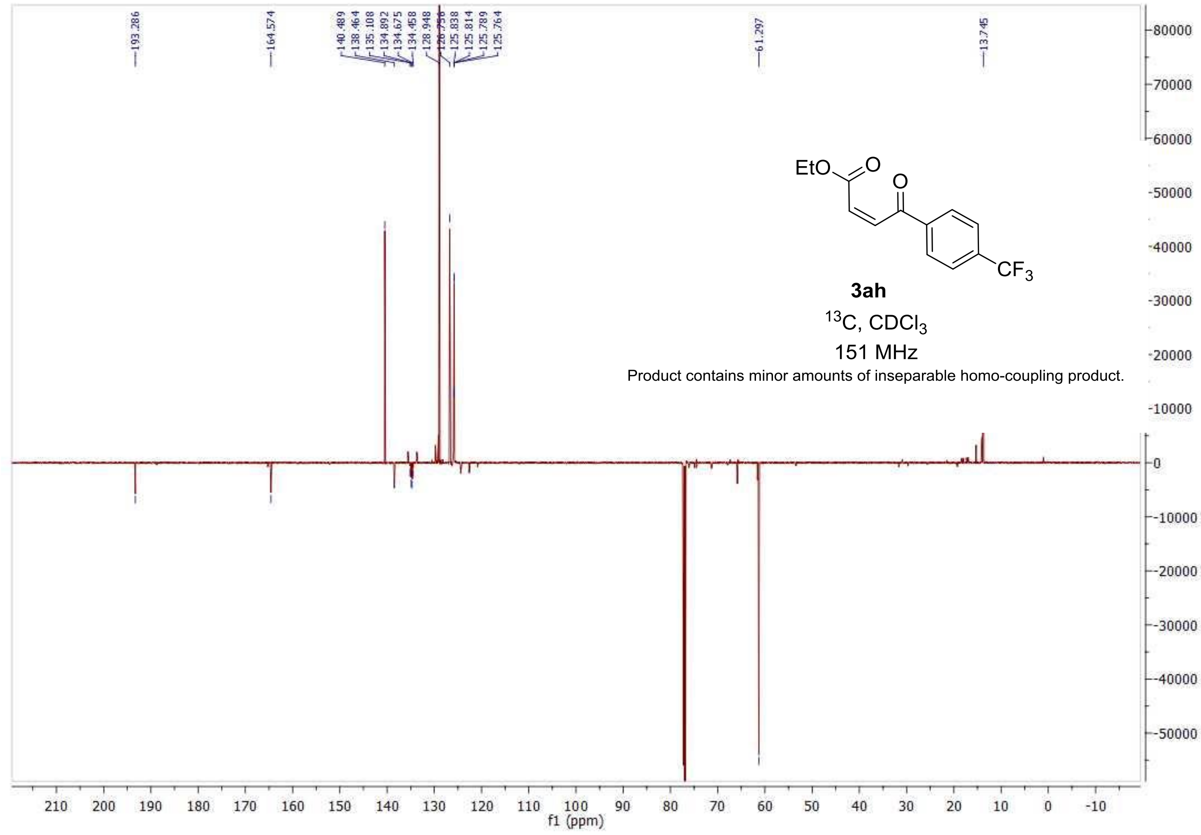
193.286
164.574
140.489
138.464
135.108
134.892
134.675
134.458
128.948
126.736
125.838
125.814
125.789
125.764
61.297
13.745
EtO
O
O
CF3
3ah
13C, CDCl3
151 MHz
Product contains minor amounts of inseparable homo-coupling product.
f1 (ppm)

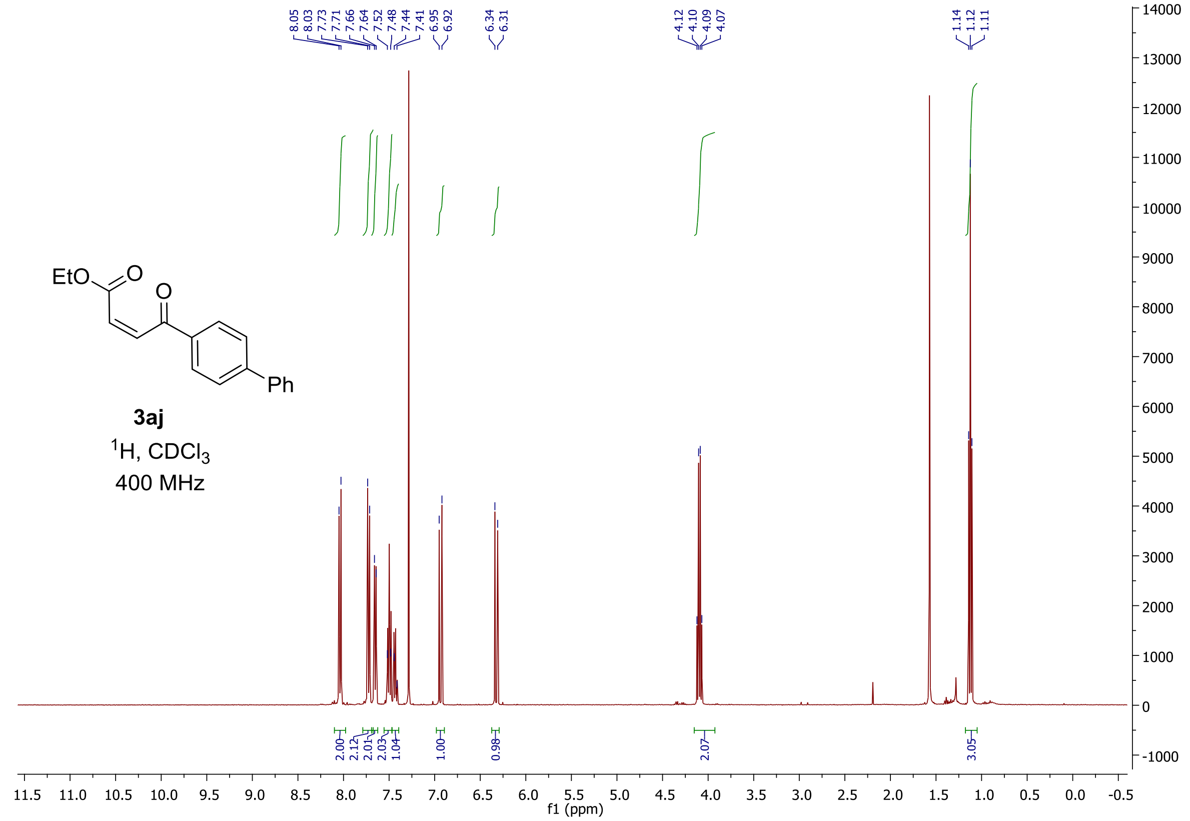
EtO
O
O
Ph
3aj
1H, CDCl3
400 MHz
f1 (ppm)

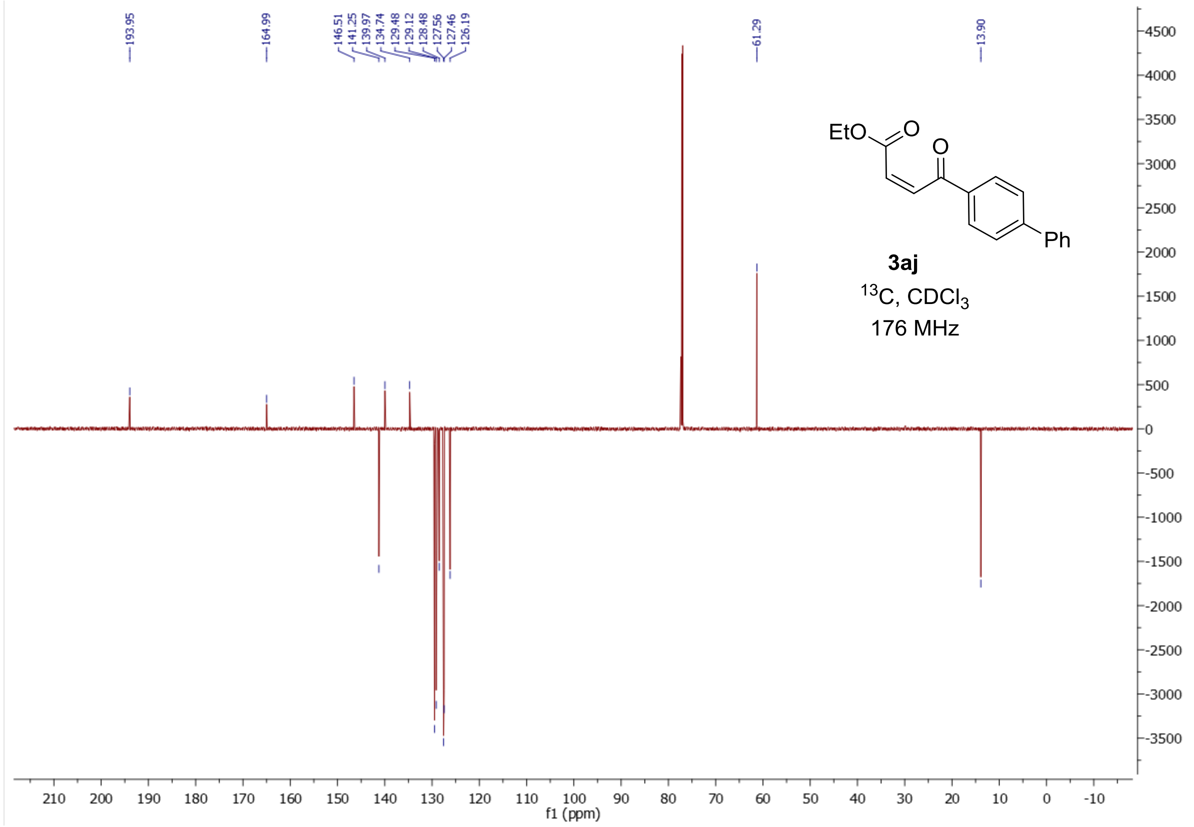
EtO
O
O
Ph
3aj
13C, CDCl3
176 MHz
f1 (ppm)

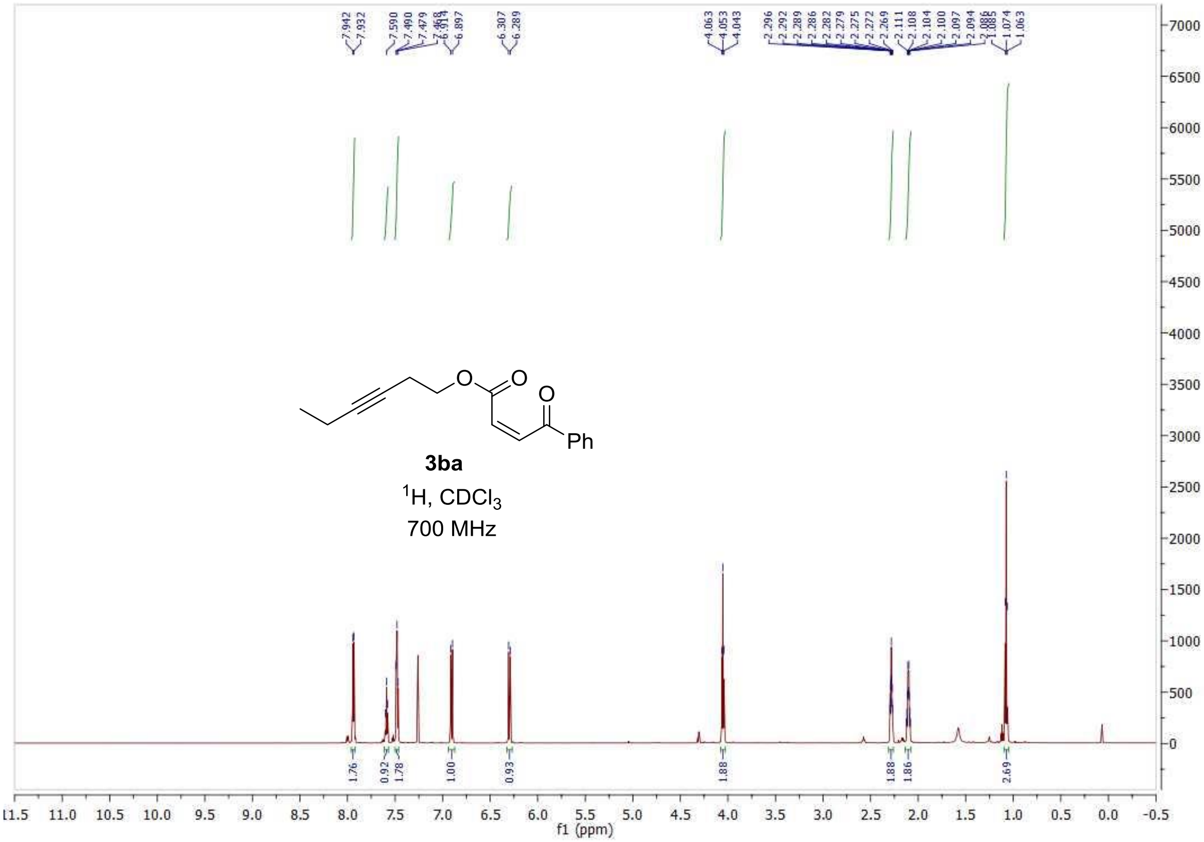
3ba
1H, CDCl3
700 MHz
f1 (ppm)

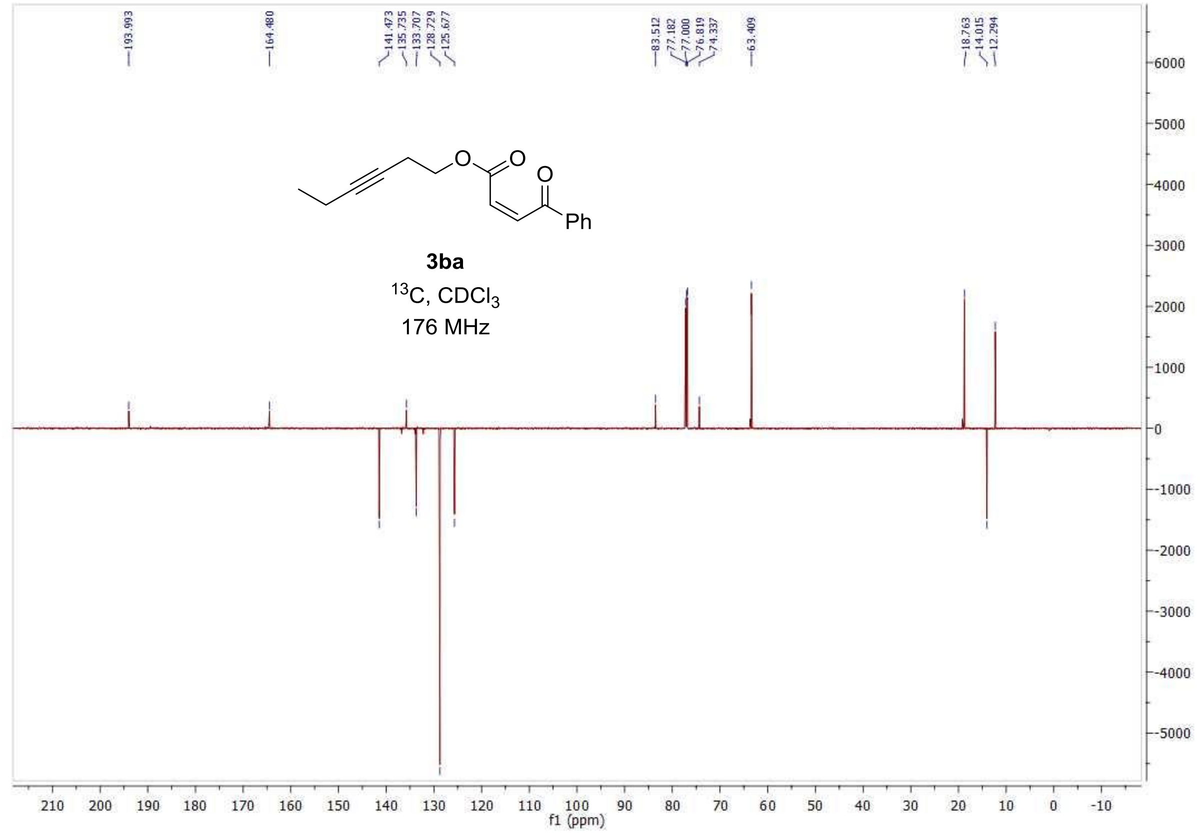
3ba
13C, CDCl3
176 MHz
f1 (ppm)

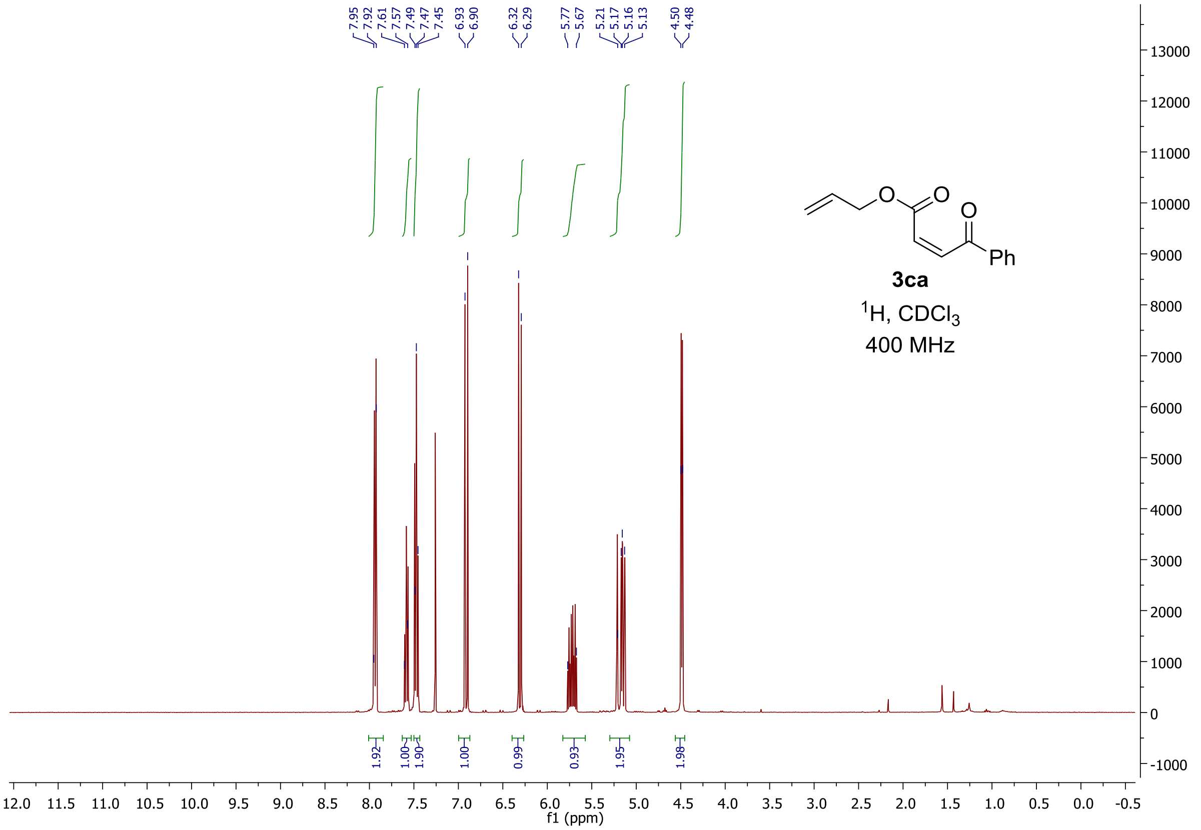
7.95
7.92
7.61
7.57
7.49
7.47
7.45
6.93
6.90
6.32
6.29
5.77
5.67
5.21
5.17
5.16
5.13
4.50
4.48
3ca
¹H, CDCl₃
400 MHz
Ph
1.92
1.00
1.90
1.00
0.99
0.93
1.95
1.98
f1 (ppm)

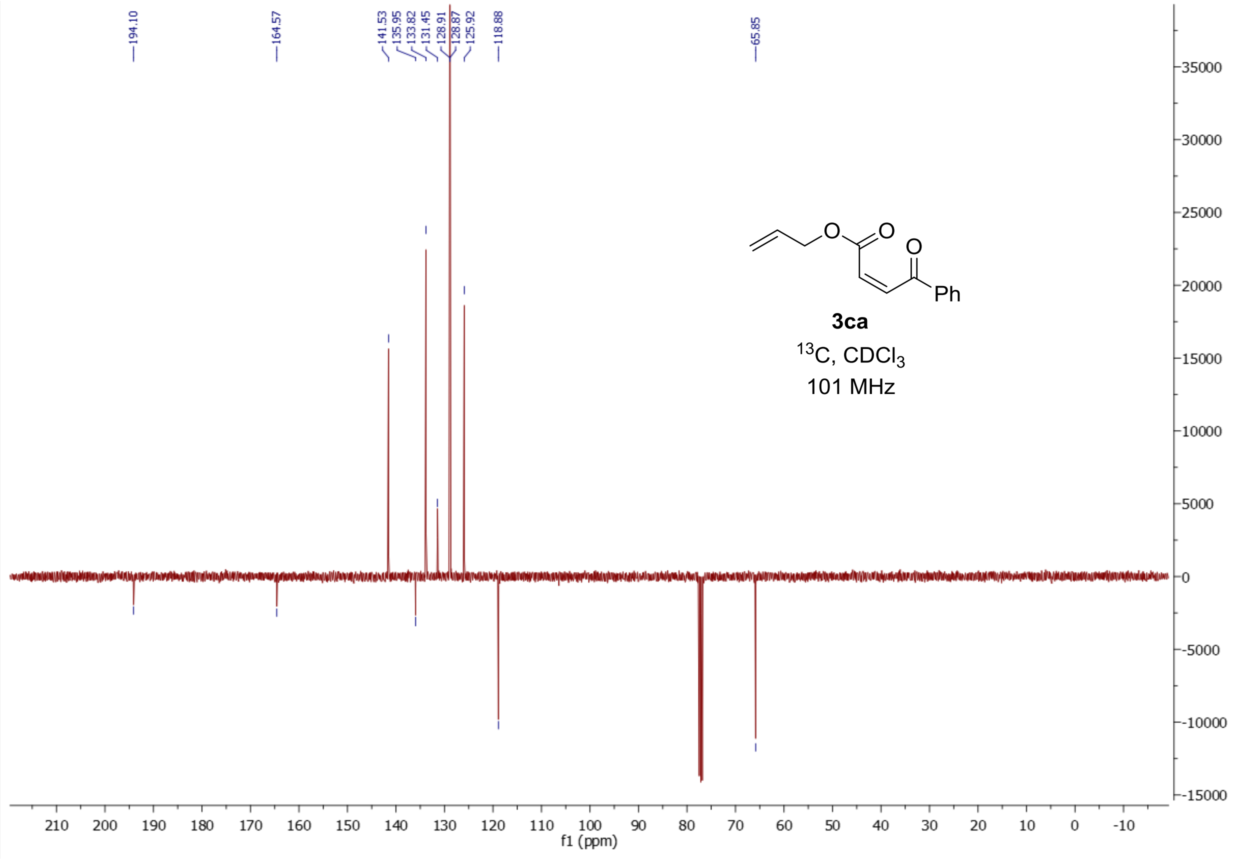
194.10
164.57
141.53
135.95
133.82
131.45
128.91
128.87
125.92
118.88
65.85
3ca
¹³C, CDCl₃
101 MHz
Ph
f1 (ppm)

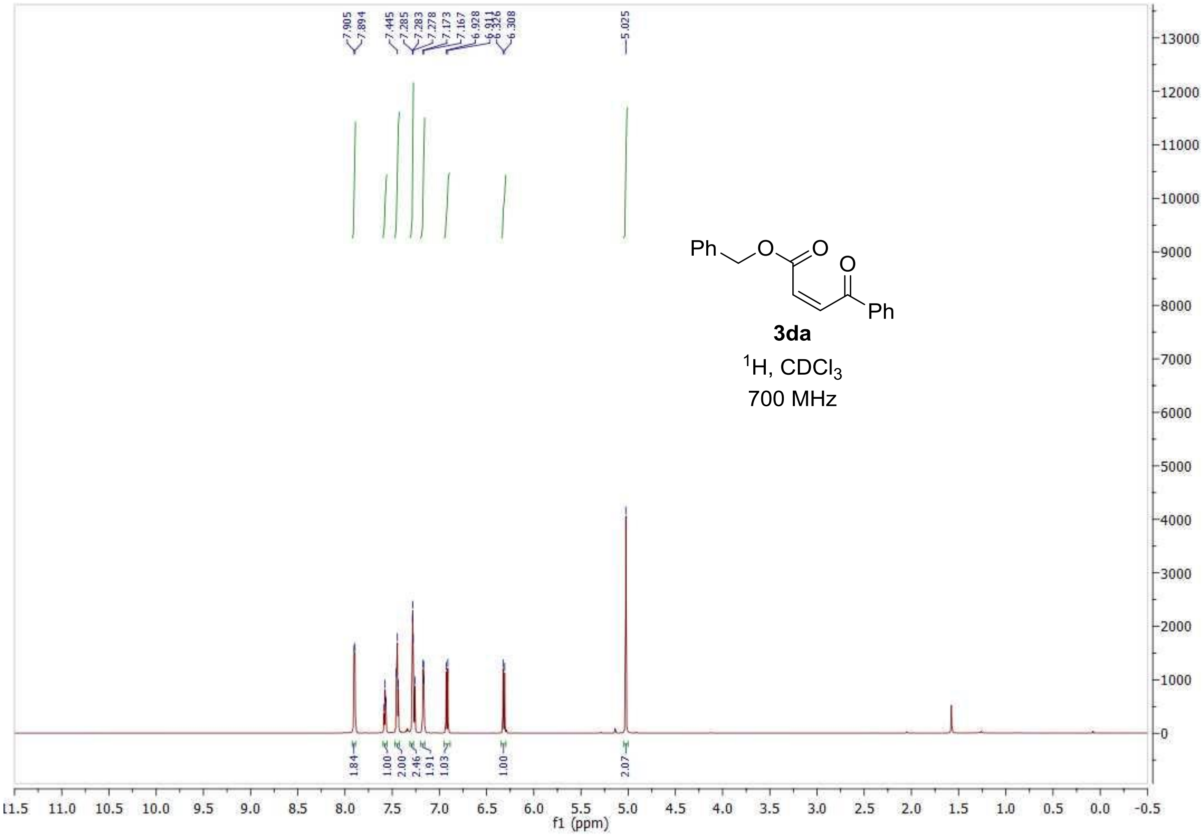
7.905
7.894
7.445
7.285
7.283
7.278
7.173
7.167
6.928
6.911
6.326
6.308
5.025
3da
^{1}H, $CDCl_3$
700 MHz
1.84
1.00
2.00
2.46
1.91
1.03
1.00
2.07
f1 (ppm)

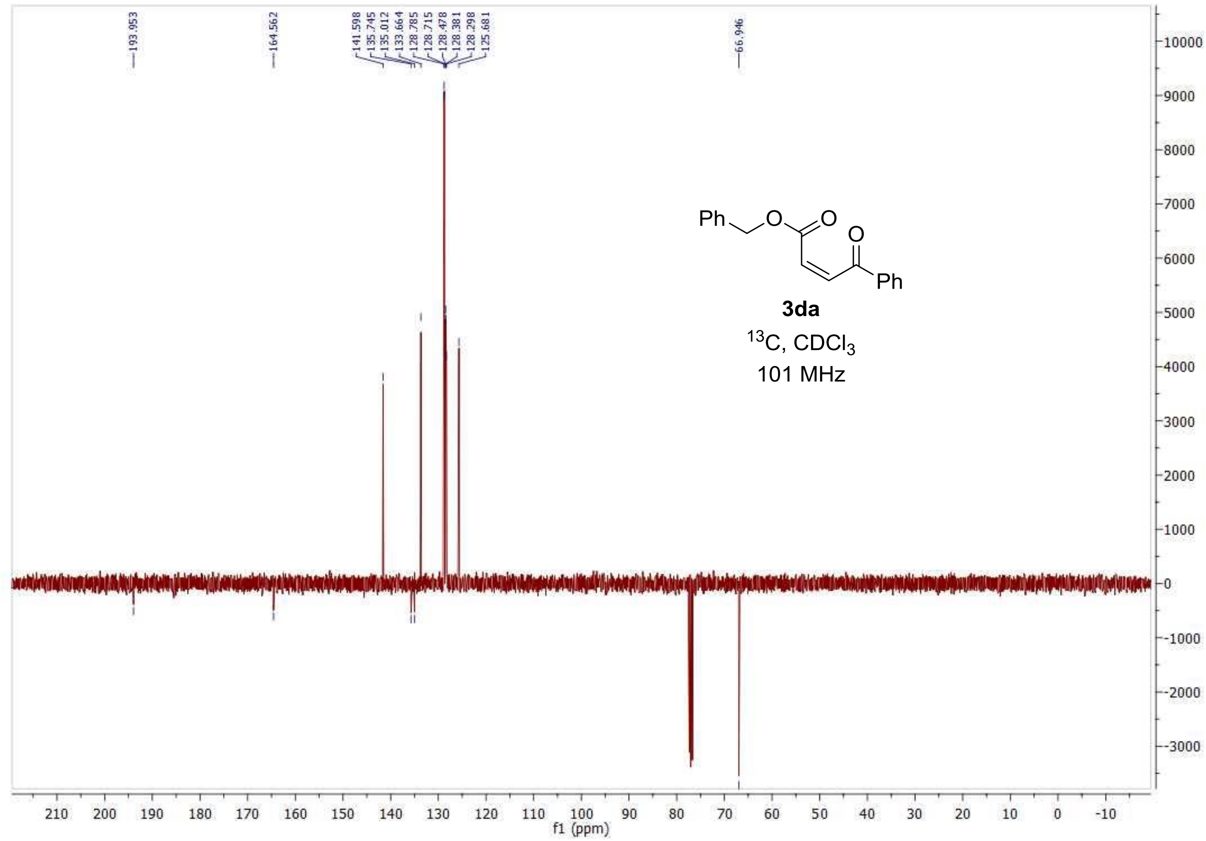
193.953
164.562
141.598
135.745
135.012
133.664
128.785
128.715
128.478
128.381
128.298
125.681
66.946
3da
^{13}C, $CDCl_3$
101 MHz
f1 (ppm)

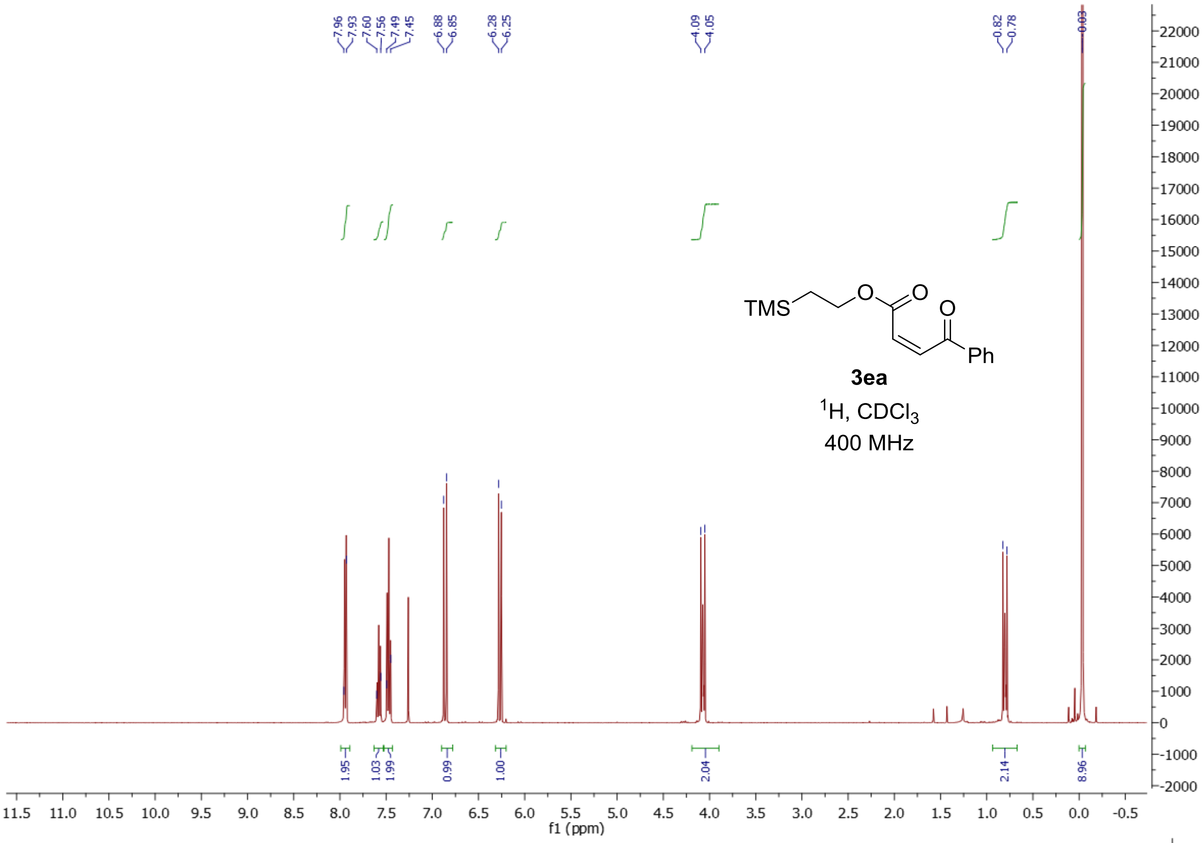
7.96
7.93
7.60
7.56
7.49
7.45
6.88
6.85
6.28
6.25
4.09
4.05
0.82
0.78
0.03
TMS
O
O
O
Ph
3ea
^{1}H, $CDCl_3$
400 MHz
1.95
1.03
1.99
0.99
1.00
2.04
2.14
8.96
f1 (ppm)

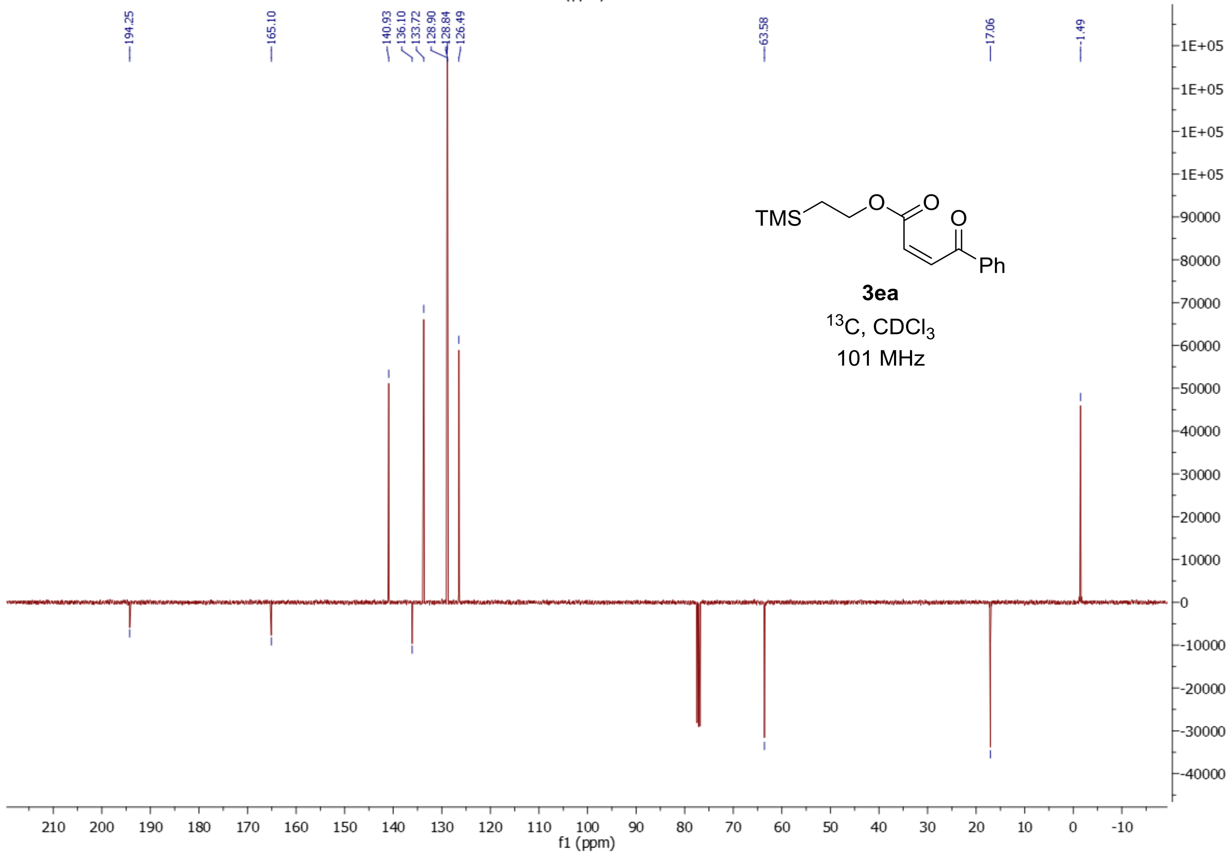
194.25
165.10
140.93
136.10
133.72
128.90
128.84
126.49
63.58
17.06
-1.49
TMS
O
O
O
Ph
3ea
^{13}C, $CDCl_3$
101 MHz
f1 (ppm)

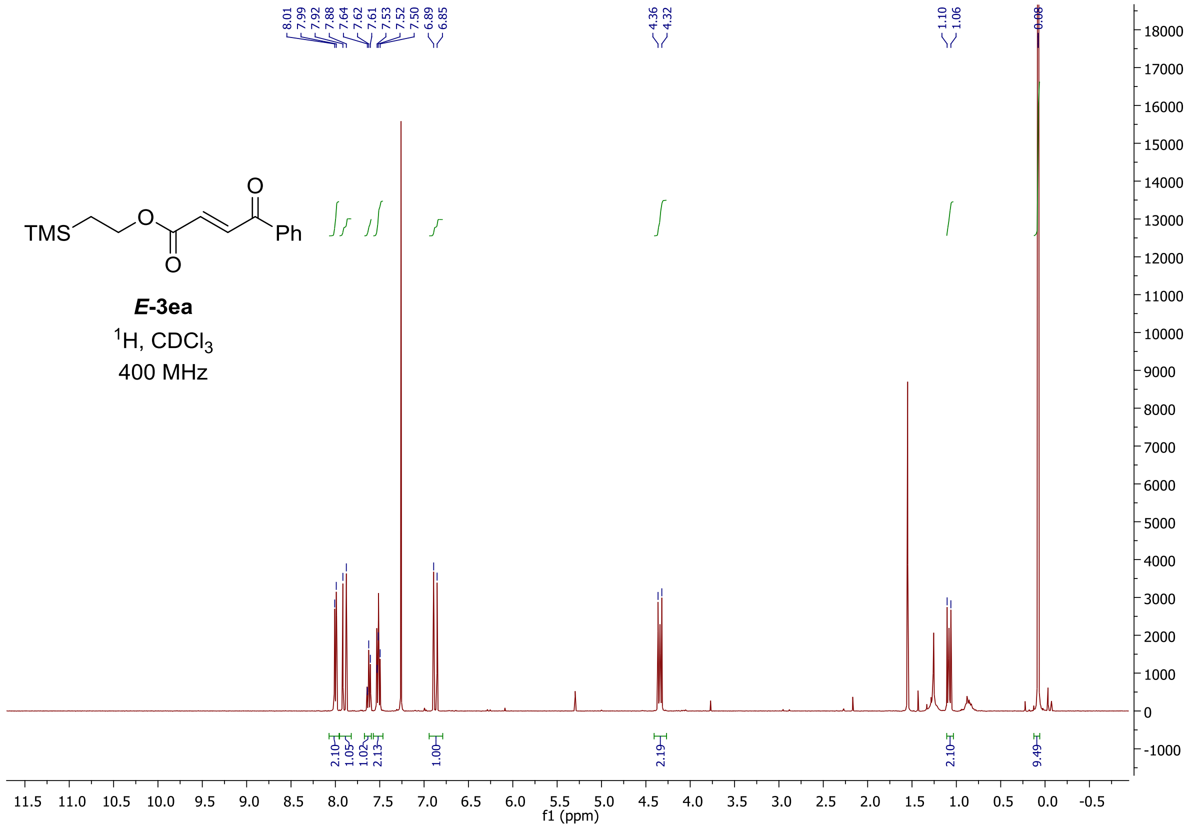
8.01
7.99
7.92
7.88
7.64
7.62
7.61
7.53
7.52
7.50
6.89
6.85
4.36
4.32
1.10
1.06
0.00
TMS
O
O
O
Ph
E-3ea
^{1}H, $CDCl_3$
400 MHz
2.10
1.05
1.02
2.13
1.00
2.19
2.10
9.49
f1 (ppm)

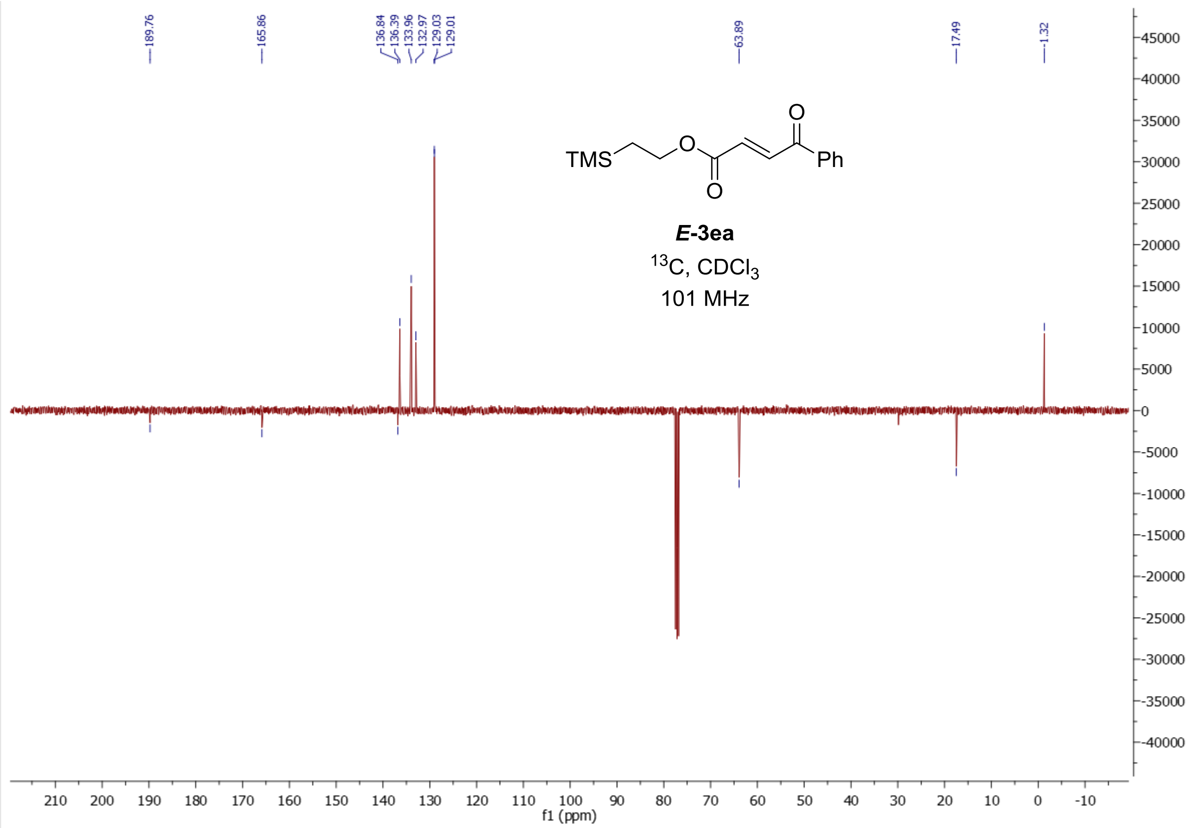
189.76
165.86
136.84
136.39
133.96
132.97
129.03
129.01
63.89
17.49
-1.32
TMS
O
O
O
Ph
E-3ea
^{13}C, $CDCl_3$
101 MHz
f1 (ppm)

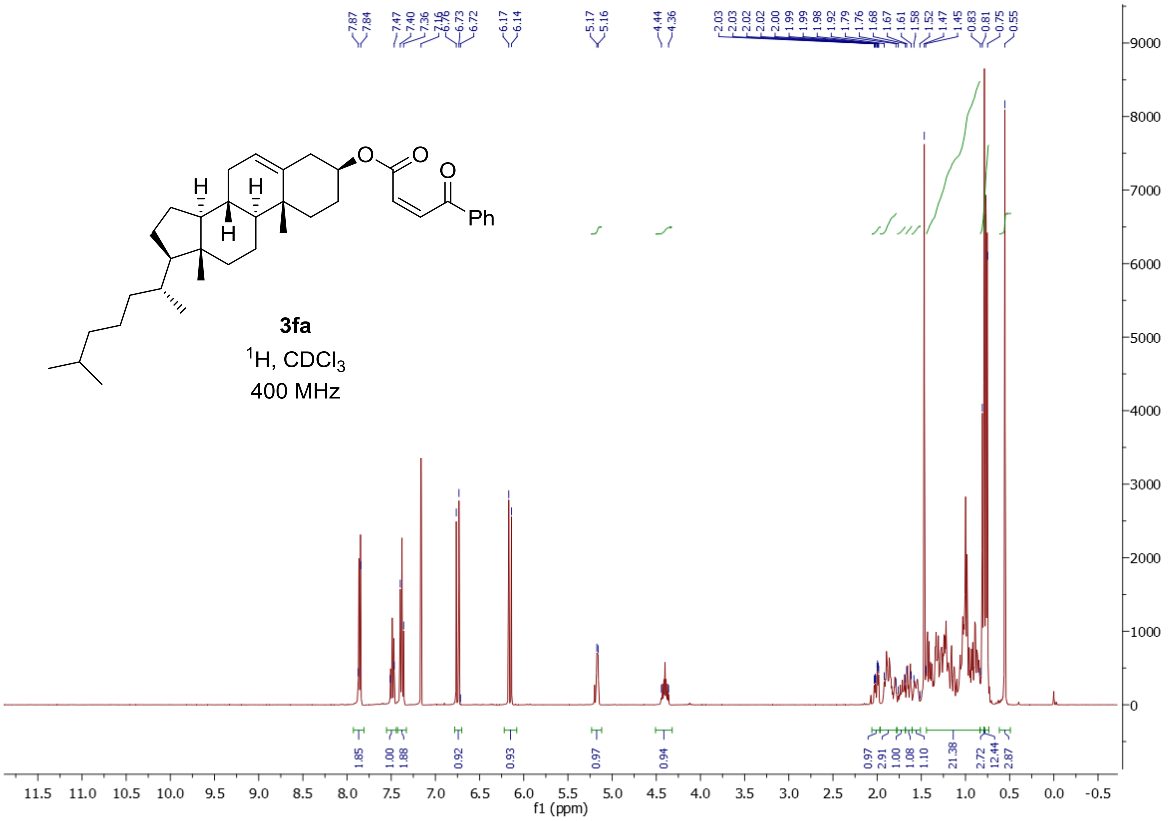
3fa
^{1}H, CDCl$_3$
400 MHz
f1 (ppm)

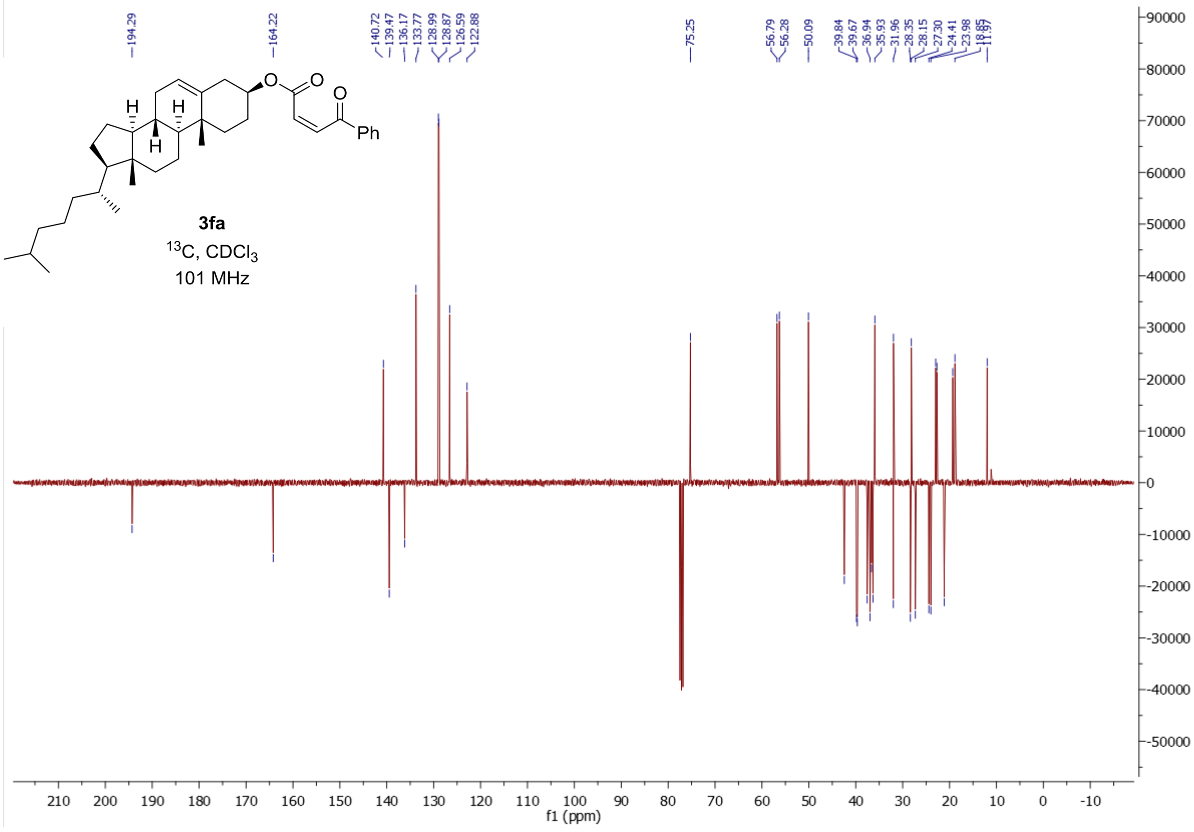
3fa
^{13}C, CDCl$_3$
101 MHz
f1 (ppm)

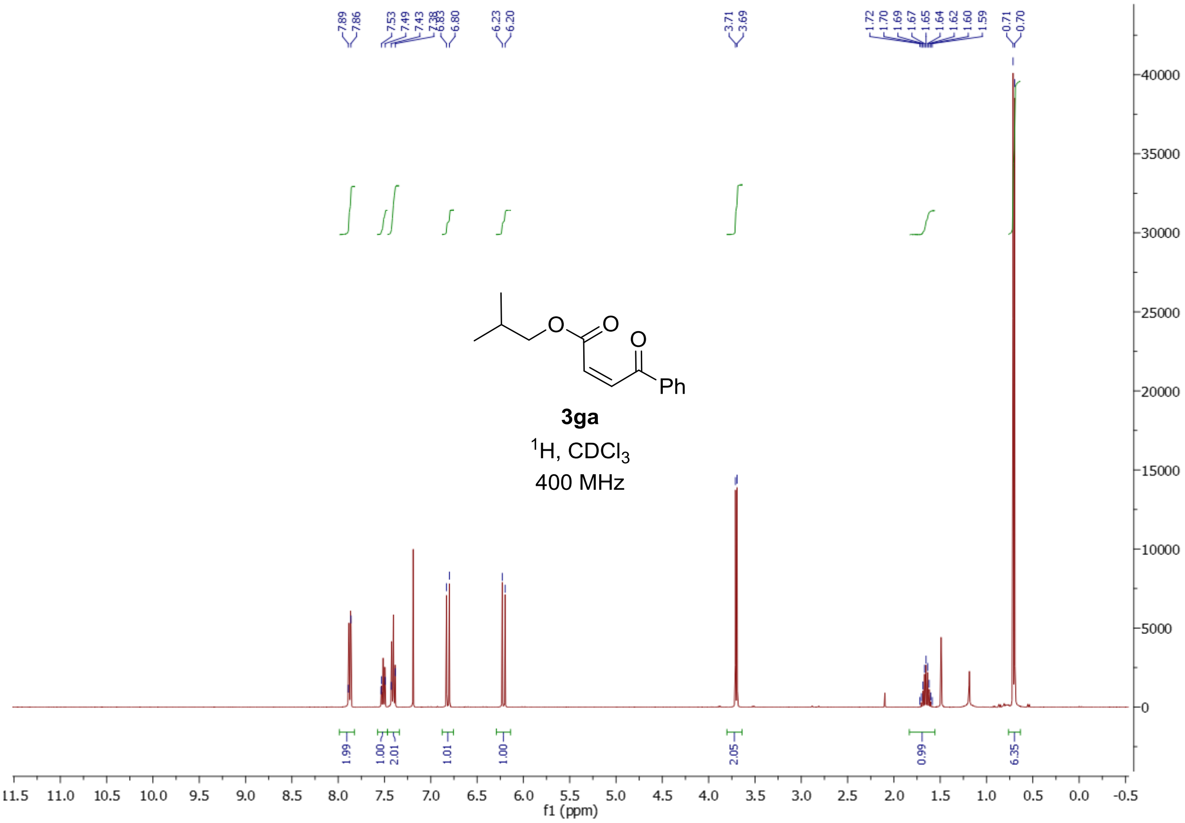
3ga
^{1}H, $CDCl_3$
400 MHz
f1 (ppm)

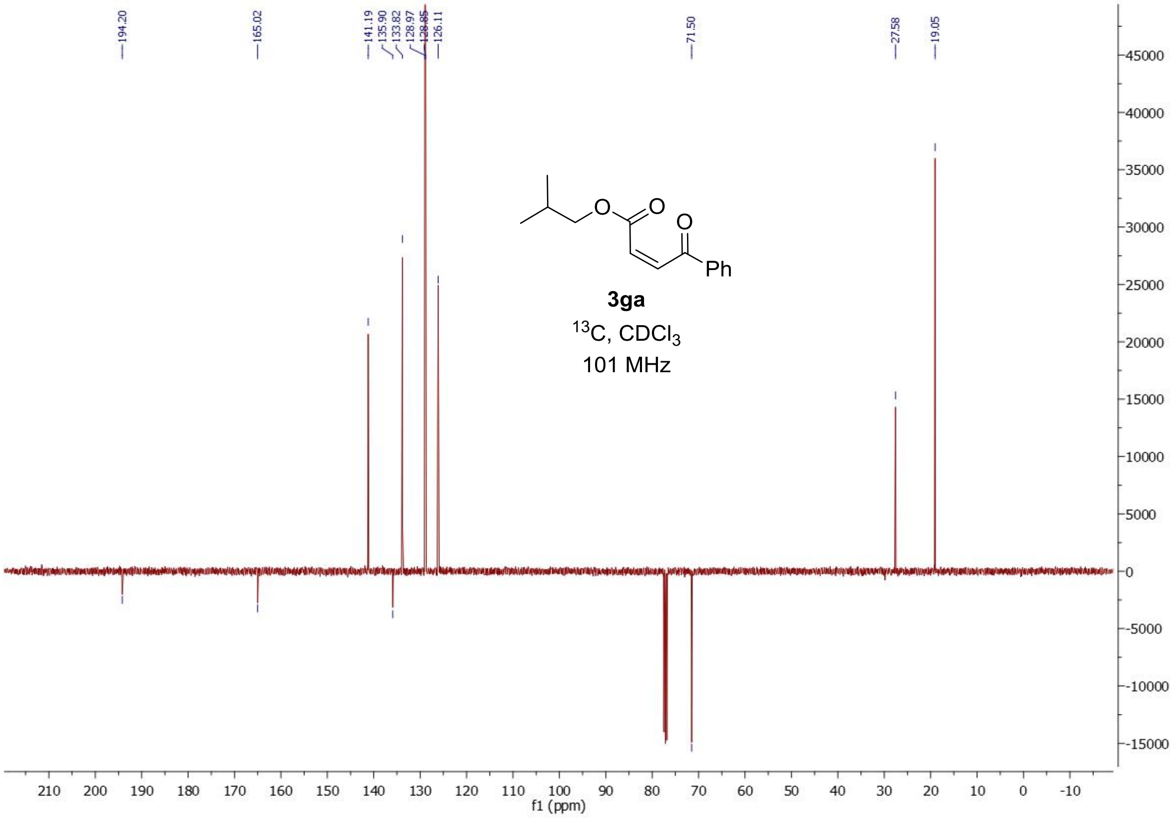
3ga
^{13}C, $CDCl_3$
101 MHz
f1 (ppm)

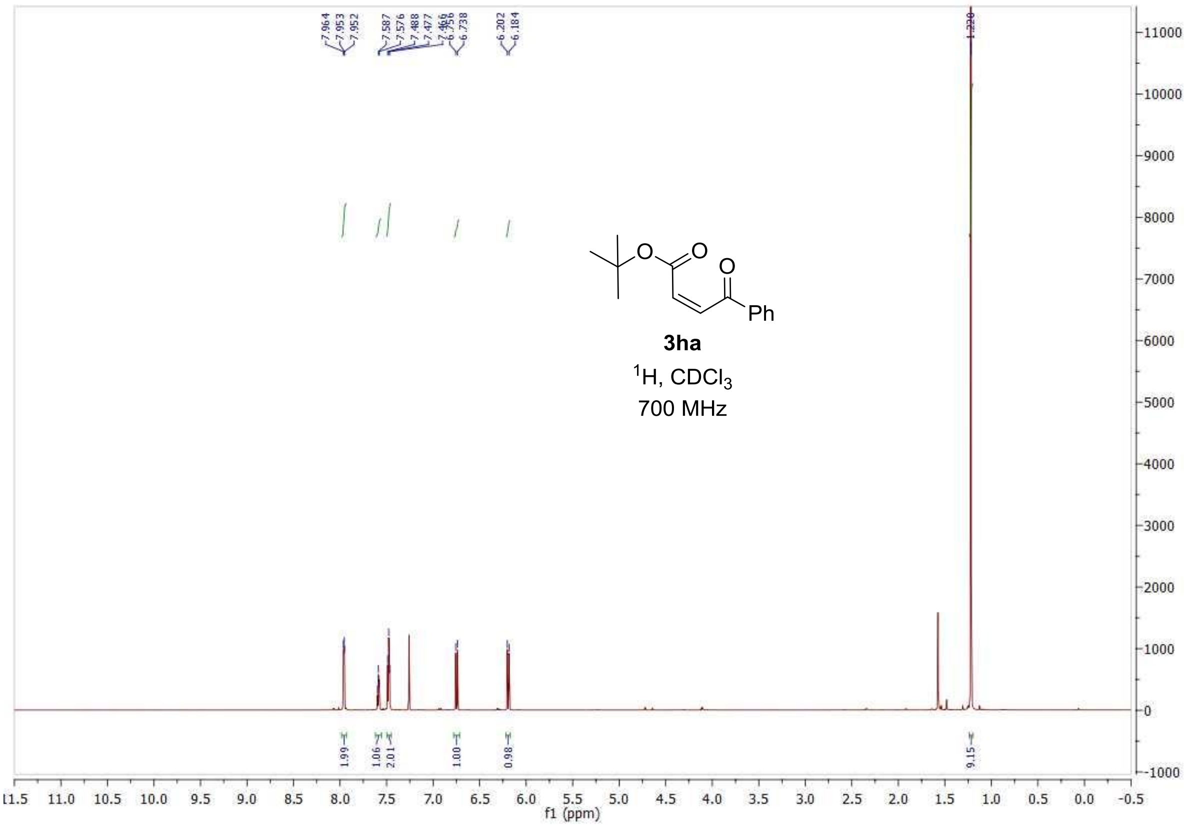
3ha
^{1}H, $CDCl_3$
700 MHz
f1 (ppm)

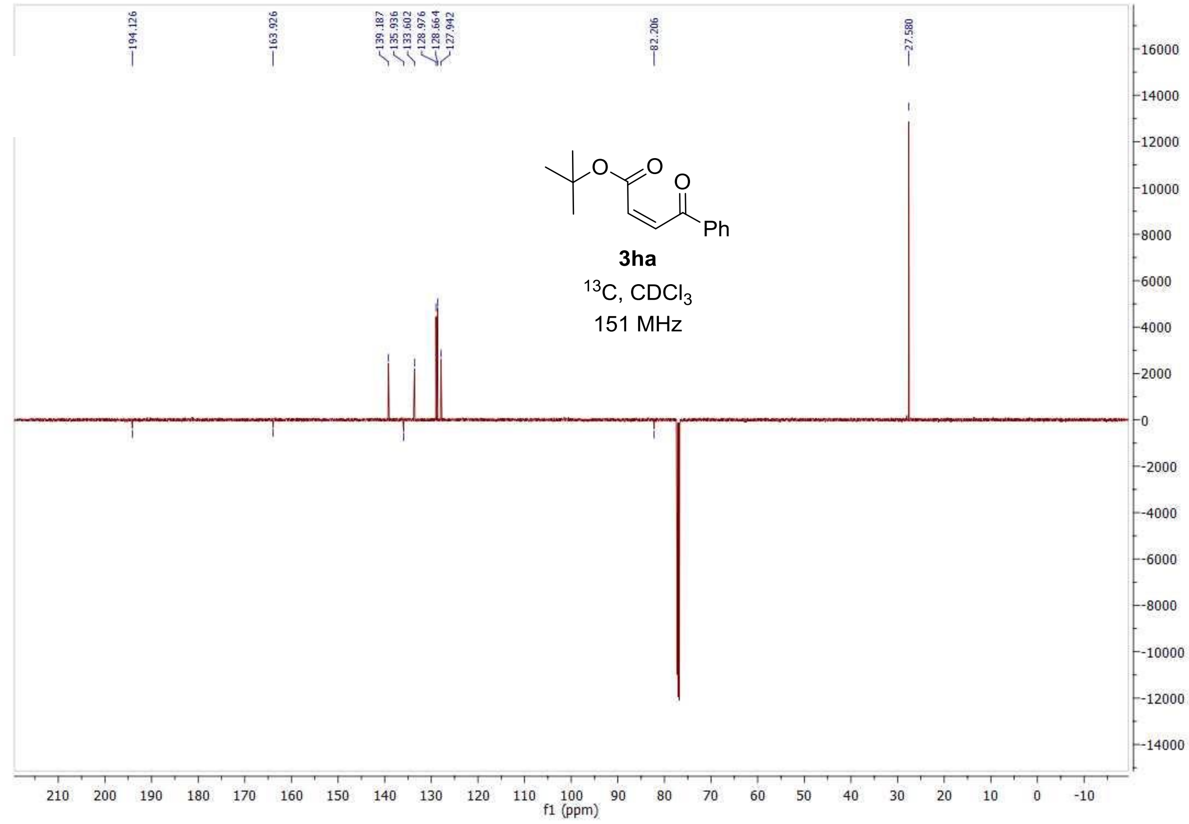
3ha
^{13}C, $CDCl_3$
151 MHz
f1 (ppm)

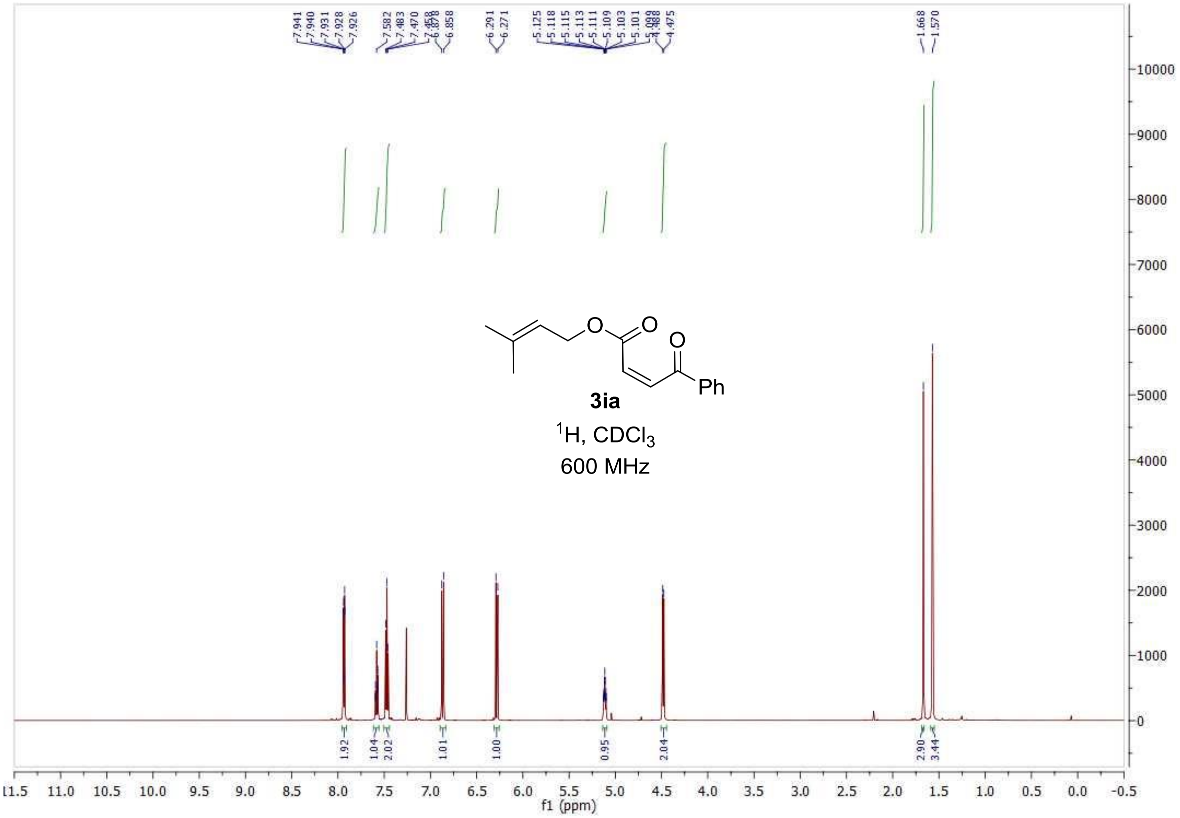
7.941
7.940
7.931
7.928
7.926
7.582
7.483
7.470
6.878
6.858
6.291
6.271
5.125
5.118
5.115
5.113
5.111
5.109
5.103
5.101
5.099
4.488
4.475
1.668
1.570
3ia
^{1}H, $CDCl_3$
600 MHz
1.92
1.04
2.02
1.01
1.00
0.95
2.04
2.90
3.44
f1 (ppm)

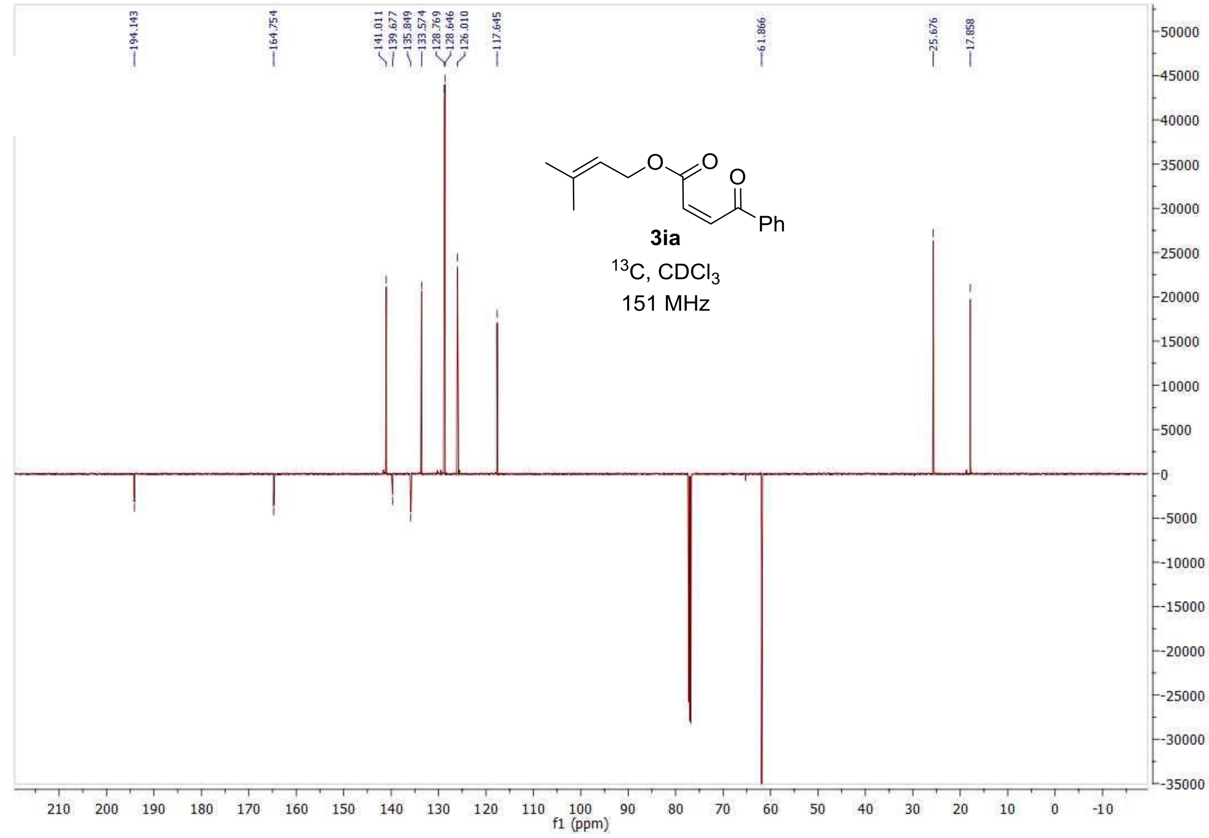
194.143
164.754
141.011
139.677
135.849
133.574
128.769
128.646
126.010
117.645
61.866
25.676
17.858
3ia
^{13}C, $CDCl_3$
151 MHz
f1 (ppm)

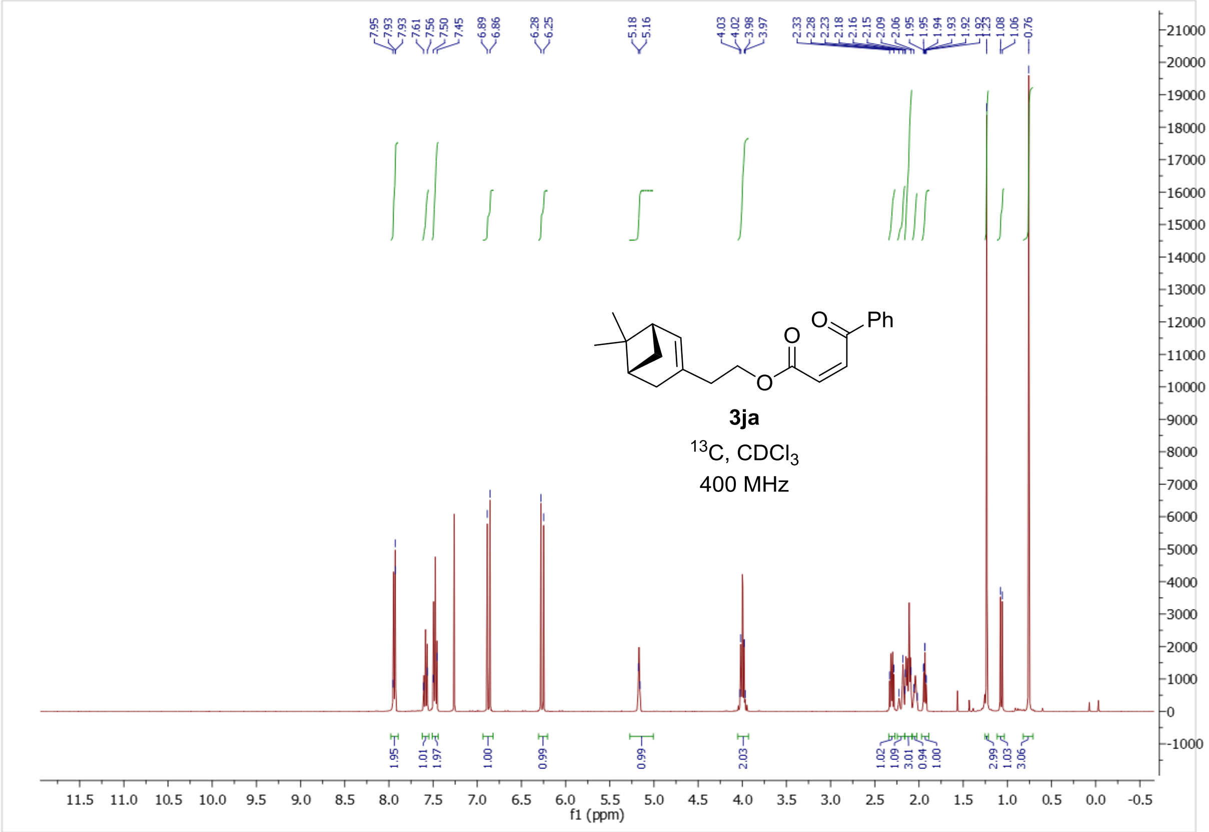
3ja
^{13}C, $CDCl_3$
400 MHz
f1 (ppm)

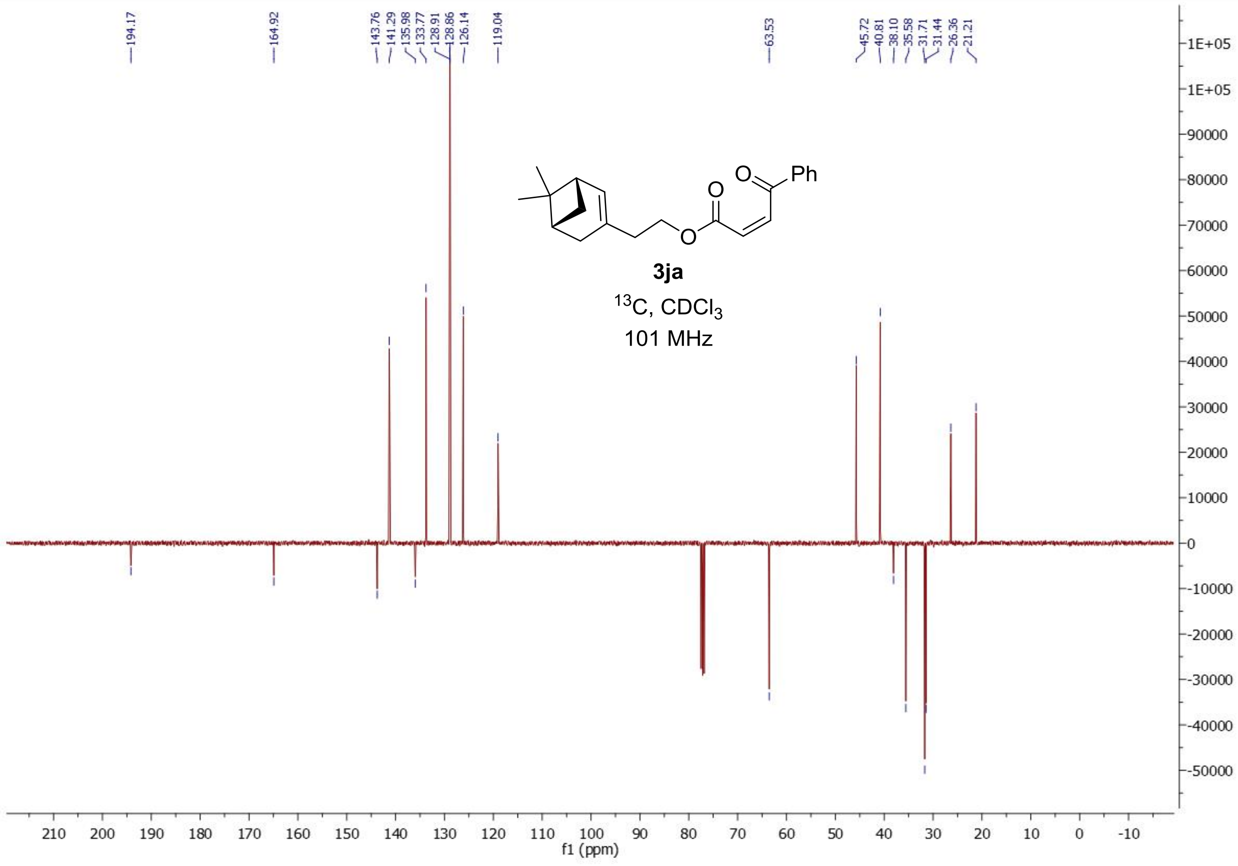
3ja
^{13}C, $CDCl_3$
101 MHz
f1 (ppm)

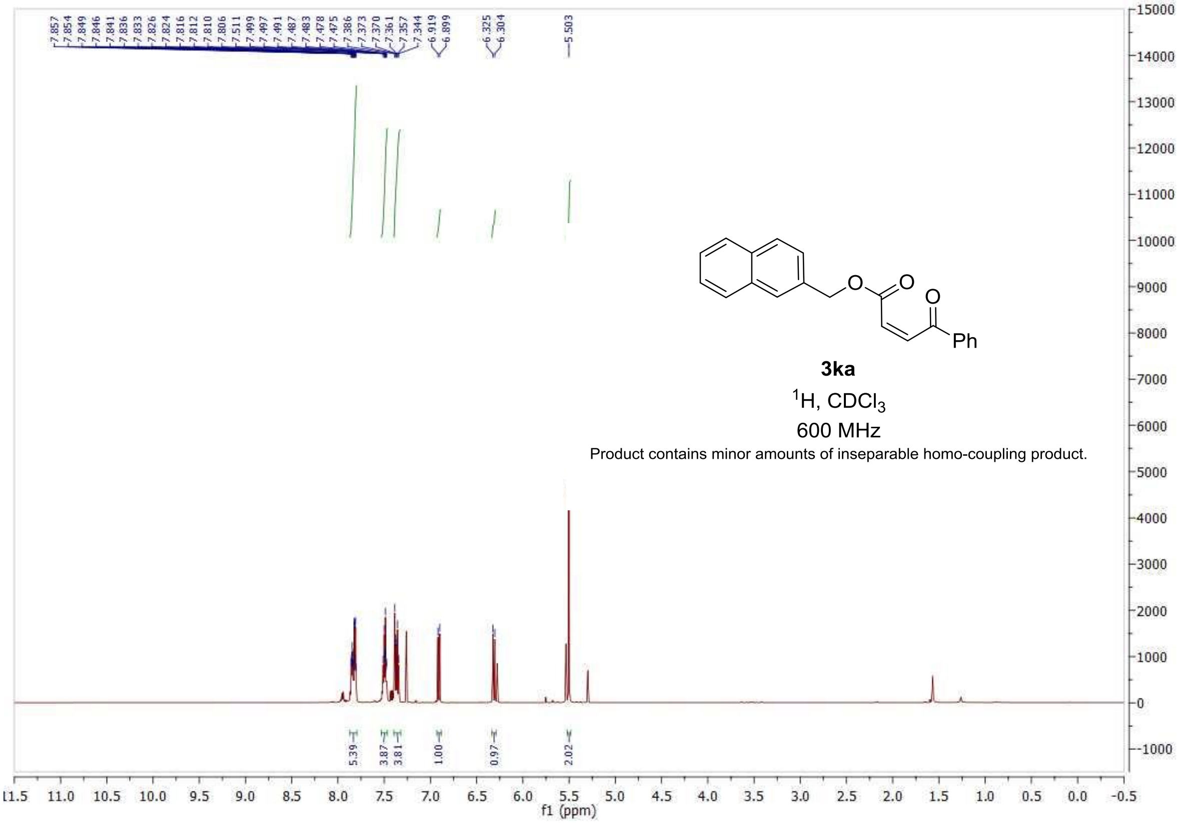
3ka
^{1}H, CDCl$_3$
600 MHz
Product contains minor amounts of inseparable homo-coupling product.
f1 (ppm)

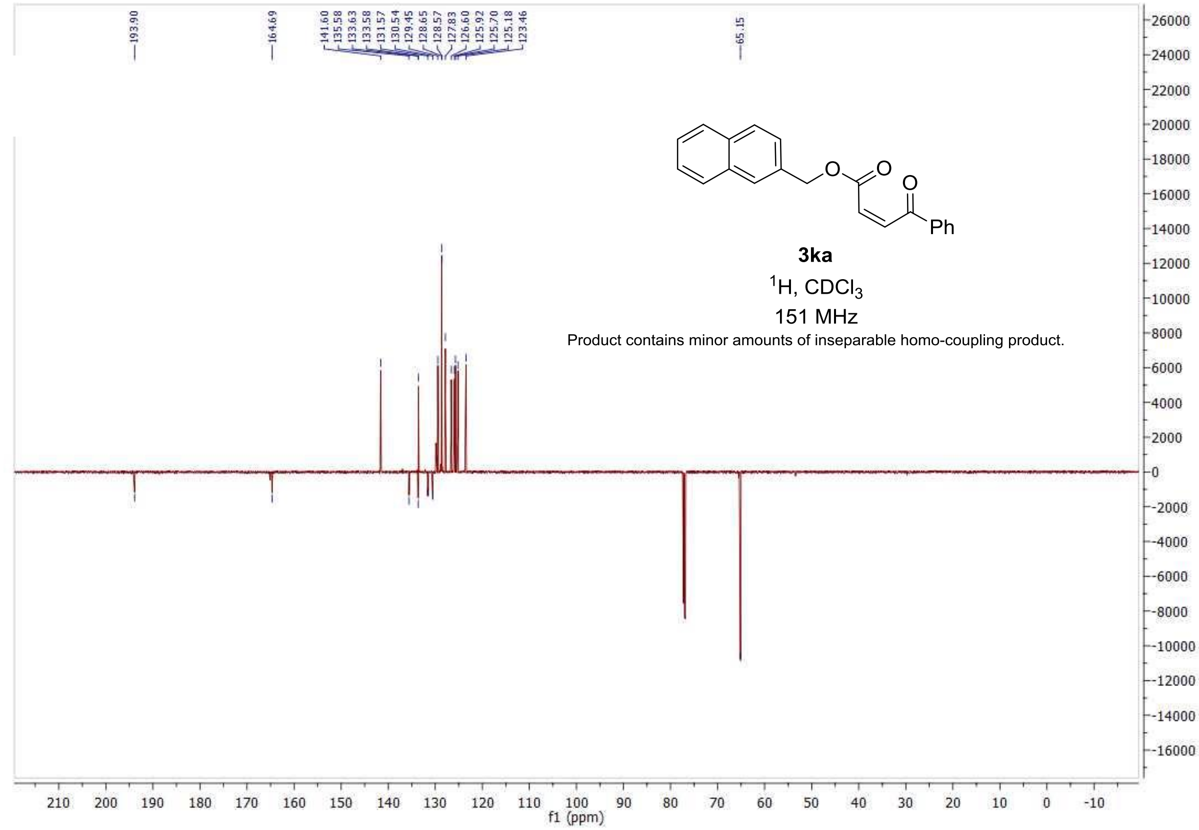
3ka
^{1}H, CDCl$_3$
151 MHz
Product contains minor amounts of inseparable homo-coupling product.
f1 (ppm)

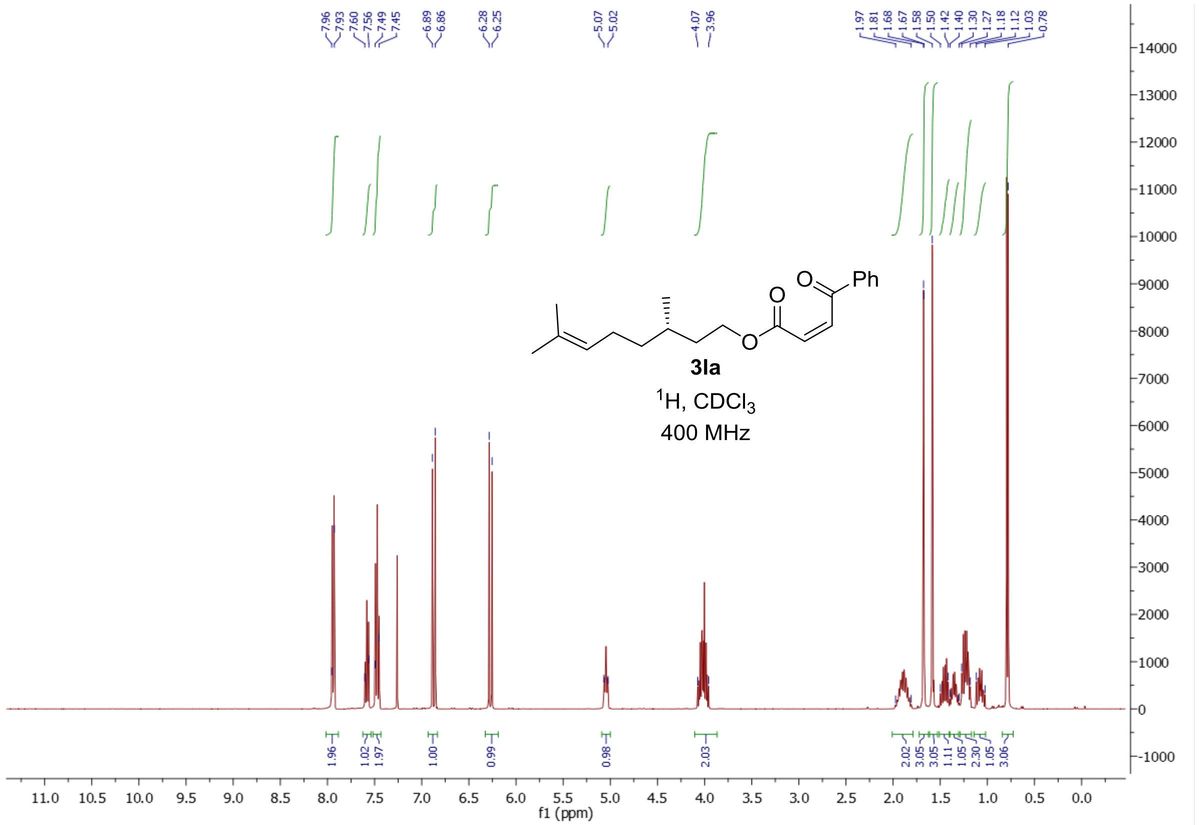
3la
^{1}H, $CDCl_3$
400 MHz
O
O
O
Ph
f1 (ppm)

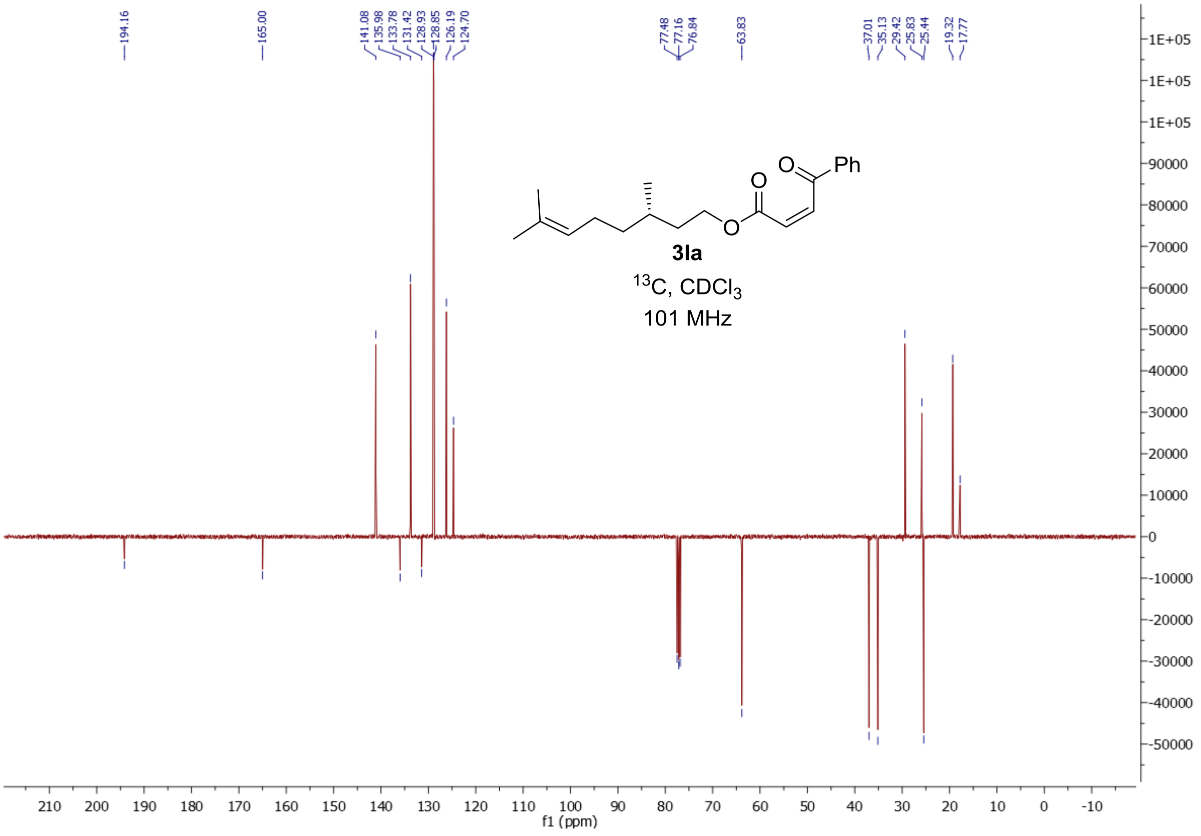
3la
^{13}C, $CDCl_3$
101 MHz
O
O
O
Ph
f1 (ppm)
